# Independent pseudogenizations and losses of *sox15* during amniote diversification following asymmetric ohnolog evolution

**DOI:** 10.1186/s12862-021-01864-z

**Published:** 2021-06-30

**Authors:** Yusaku Ogita, Kei Tamura, Shuuji Mawaribuchi, Nobuhiko Takamatsu, Michihiko Ito

**Affiliations:** 1 Department of Bioscience, School of Science, Kitasato University, 1-15-1 Kitasato, Minamiku Sagamihara, Kanagawa, 252-0373 Japan; 2 Cellular and Molecular Biotechnology Research Institute, National Institute of Advanced Industrial Science and Technology (AIST), Central 6, 1-1-1 Higashi, Tsukuba, Ibaraki 305-8566 Japan

**Keywords:** Ohnolog, Ortholog, 2R-WGD, Pseudogene, Gene loss, Neofunctionalization, Relax, *d*_N_/*d*_S_, Marsupial, Reptile

## Abstract

**Background:**

Four ohnologous genes (*sox1*, *sox2*, *sox3*, and *sox15*) were generated by two rounds of whole-genome duplication in a vertebrate ancestor. In eutherian mammals, *Sox1*, *Sox2*, and *Sox3* participate in central nervous system (CNS) development. *Sox15* has a function in skeletal muscle regeneration and has little functional overlap with the other three ohnologs. In contrast, the frog *Xenopus laevis* and zebrafish orthologs of *sox15* as well as *sox1-3* function in CNS development. We previously reported that *Sox15* is involved in mouse placental development as neofunctionalization, but is pseudogenized in the marsupial opossum. These findings suggest that *sox15* might have evolved with divergent gene fates during vertebrate evolution. However, knowledge concerning *sox15* in other vertebrate lineages than therian mammals, anuran amphibians, and teleost fish is scarce. Our purpose in this study was to clarify the fate and molecular evolution of *sox15* during vertebrate evolution.

**Results:**

We searched for *sox15* orthologs in all vertebrate classes from agnathans to mammals by significant sequence similarity and synteny analyses using vertebrate genome databases. Interestingly, *sox15* was independently pseudogenized at least twice during diversification of the marsupial mammals. Moreover, we observed independent gene loss of *sox15* at least twice during reptile evolution in squamates and crocodile-bird diversification. Codon-based phylogenetic tree and selective analyses revealed an increased* d*_N_/*d*_S_ ratio for *sox15* compared to the other three ohnologs during jawed vertebrate evolution.

**Conclusions:**

The findings revealed an asymmetric evolution of *sox15* among the four ohnologs during vertebrate evolution, which was supported by the increased *d*_N_/*d*_S_ values in cartilaginous fishes, anuran amphibians, and amniotes. The increased *d*_N_/*d*_S_ value of *sox15* may have been caused mainly by relaxed selection. Notably, independent pseudogenizations and losses of *sox15* were observed during marsupial and reptile evolution, respectively. Both might have been caused by strong relaxed selection. The drastic gene fates of *sox15,* including neofunctionalization and pseudogenizations/losses during amniote diversification, might be caused by a release from evolutionary constraints.

**Supplementary Information:**

The online version contains supplementary material available at 10.1186/s12862-021-01864-z.

## Background

In mammals, the *Sex-determining region Y* (*Sry*)-type high mobility group (HMG) box (*Sox*) family of genes includes approximately 20 members. The family is divided into eight groups (A–H) based on sequence identity in the DNA-binding domain HMG box [1, 2]. Interestingly, groups A and G contain only one member, *Sry* and *Sox15*, respectively. In contrast, most *Sox* groups are comprised of three closely related genes. Notably, *Sry* and *sox15* may have diverged independently from the *sox3* orthologs in common ancestors of therian mammals and vertebrates, respectively [3, 4]. *Sox3* shares higher sequence identity with *Sox1* or *Sox2* than *Sox15* in mammals, with group B1 comprising three members (*Sox1, Sox2,* and *Sox3*) [1, 5]. Vertebrate ohnologs are paralogs generated through the two rounds of whole-genome duplication (2R-WGD) in the common ancestor of vertebrates [6–8]. Synteny analysis data revealed ohnologous relationships for these four members. The two ancestral genes *sox1/2* and *sox3/15* emerged from *soxB1* in the common ancestor of vertebrates in the first round of WGD, followed by the generation of these four genes in the second round of WGD [4]. Mammalian *Sox15* has orthologous relationships with teleost *sox19a/b* and amphibian *soxD* (now termed *sox15*), although *sox19a/b* and *soxD/sox15* belong to groups B1 and I, respectively [4]. The pairwise sequence identities between *sox15*, *sox19a/b,* and *soxD/sox15* are not particularly high, which likely resulted in the different gene names conferred during an era of limited genome information.

Both zebrafish *sox19a/b* and the African clawed frog *Xenopus laevis soxD/sox15* have high expression levels and function in central nervous system (CNS) development [4, 9, 10]. There are, to our knowledge, no reports of mammalian *sox15* involvement in neurogenesis. In contrast, mammalian *Sox15* is expressed in embryonic stem cells and satellite cells involved in muscle regeneration [11, 12]. Moreover, we described the specific expression of *Sox15* in the placenta during mouse embryogenesis in placenta-derived trophoblast giant cells and placental stem cells, and demonstrated that *Sox15* became pseudogenized in the marsupial opossum [5, 13, 14]. Based on the findings of *Sox15* orthologs in mammals, actinopterygian (bony) fish and amphibians, we concluded that *Sox15* evolved through neofunctionalization for placental formation and/or myogenesis in eutherian diversification [5]. Therefore, there appears to be little functional overlap between *sox15* and *sox19a/b* or *soxD/sox15* orthologs.

The collective findings indicate that *sox15* orthologs appear to have evolved dramatically [5]. However, little is known about *sox15* in birds, reptiles, non-eutherian mammals, non-anuran amphibians, and chondrichthyan fishes. To clarify the molecular evolution of *sox15* orthologs, we retrieved orthologous sequences of *sox15* from genome databases of amniote vertebrates, urodele/apoda amphibians, and chondrichthyan fishes, and performed synteny analysis. Notably, we identified independent losses and pseudogenizations of *sox15* in reptiles, including birds and marsupial mammals, respectively. The fates of *sox15* with independent pseudogenization/loss and neofunctionalization represent a rare example of gene fate during vertebrate evolution, although *bmp16* was recently reported as an example of independent gene loss among the three ohnologs [15, 16]. Our evolutionary analyses revealed the highest *d*_N_/*d*_S_ value of *sox15* among the four ohnologs and its independent pseudogenizations/losses during amniote diversification.

## Results

### Independent pseudogenizations of *Sox15* during marsupial mammalian evolution

**Fig. 1:**
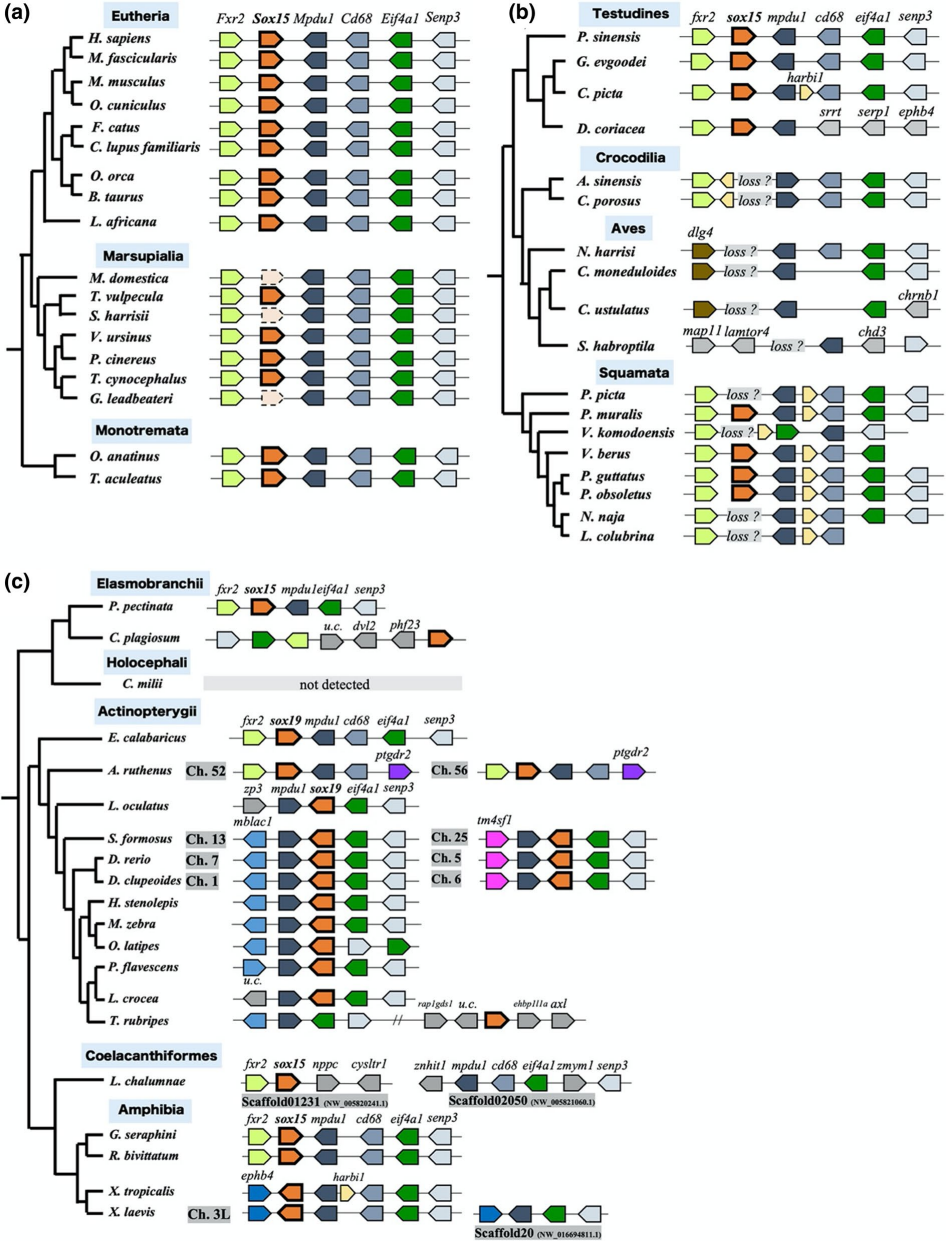
Incomplete retention of *sox15* during amniote evolution. Summary of synteny analysis of (**a**) mammalian, (**b**) reptilian, and (**c**) other gnathostomatan orthologs of *sox15* using 56 Gnathostomata species. *H. sapiens*, *Homo sapiens*; *M. fascicularis*, *Macaca fascicularis*; *M. musculus*, *Mus musculus*; *O. cuniculus*, *Oryctolagus cuniculus*; *F. catus*, *Felis catus*; *C. lupus familiaris*, *Canis lupus familiaris*; *O. orca*, *Orcinus orca*; *B. taurus*, *Bos taurus*; *L. africana*; *Loxodonta africana*; *M. domestica*, *Monodelphis domestica*; *T. vulpecula*, *Trichosurus vulpecula*; *S. harrisii*, *Sarcophilus harrisii*; *V. ursinus*, *Vombatus ursinus*; *P. cinereus*, *Phascolarctos cinereus*; *T. cynocephalus*, *Thylacinus cynocephalus*; *G. leadbeateri*, *Gymnobelideus leadbeateri*; *O. anatinus*, *Ornithorhynchus anatinus*; *T. aculeatus*, *Tachyglossus aculeatus*; *P. sinensis*, *Pelodiscus sinensis*; *G. evgoodei*, *Gopherus evgoodei*; *C. picta*, *Chrysemys picta*; *D. coriacea*, *Dermochelys coriacea*; *A. sinensis*, *Alligator sinensis*; *C. porosus*, *Crocodylus porosus*; *N. harrisi*, *Nannopterum harrisi*; *C. moneduloides*, *Corvus moneduloides*; *C. ustulatus*, *Catharus ustulatus*; *S. habroptila*, *Strigops habroptila*; *P. picta*, *Paroedura picta*; *P. muralis*, *Podarcis muralis*; *V. komodoensis*, *Varanus komodoensis*; *V. berus*, *Vipera berus*; *P. vitticeps*, *Pogona vitticeps*; *P. obsoletus*, *Pantherophis obsoletus*; *N. naja*, *Naja naja*; *L. colubrina*, *Laticauda colubrina*; *P. pectinata*, *Pristis pectinata*; *C. plagiosum*, *Chiloscyllium plagiosum*; *C. milii*, *Callorhinchus milii*; *E. calabaricus*, *Erpetoichthys calabaricus*; *A. ruthenus*, *Acipenser ruthenus*; *L. oculatus*, *Lepisosteus oculatus*; *S. formosus*, *Scleropages formosus*; *D. rerio*, *Danio rerio*; *D*. *clupeoides*, *Denticeps clupeoides*; *H. stenolepis*, *Hippoglossus stenolepis*; *M*. *zebra*, *Maylandia zebra*; *O*. *latipes*, *Oryzias latipes*; *P*. *flavescens*, *Perca flavescens*; *L*. *crocea*, *Larimichthys crocea*; *T*. *rubripes*, *Takifugu rubripes*; *L*. *chalumnae*, *Latimeria chalumnae*; *G*. *seraphini*, *Geotrypetes seraphini*; *R*. *bivittatum*, *Rhinatrema bivittatum*; *X*. *tropicalis*, *Xenopus tropicalis*; and *X*. *laevis*, *Xenopus laevis*

**Fig. 2:**
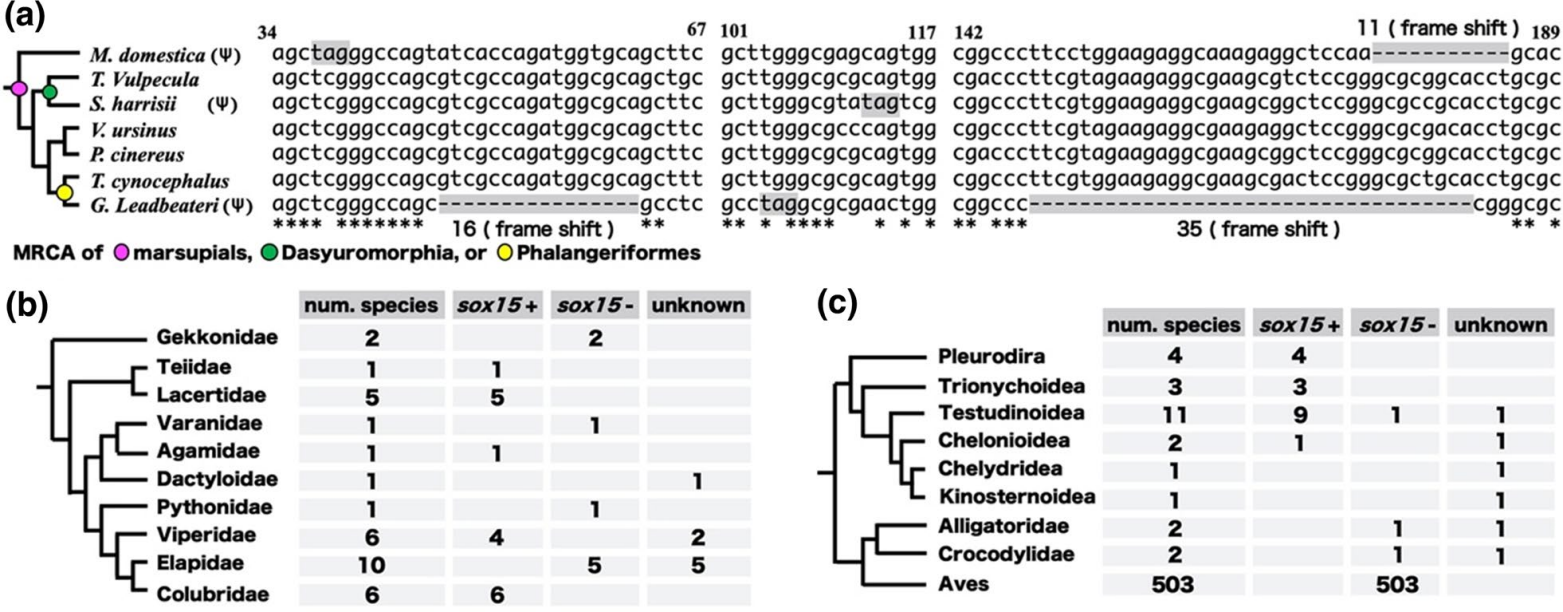
Independent pseudogenizations or losses of *sox15* during marsupial or reptilian speciation. **a** Independent pseudogenization of *sox15* during marsupial speciation. (ψ) indicates pseudogenization of *sox15*. Numbers from the first nucleotide in 225 nucleotide sequences encoding the HMG box are shown in the nucleotide alignment. In-frame stop codons and deletions with frame shift mutation are highlighted by gray boxes. Asterisks denote identical nucleotides among seven species following alignment. MRCA denotes most recent common ancestor. **b**, **c** Independent losses of *sox15* in two lineages during reptilian speciation: Squamata including lizards and snakes (**b**) and Archosauromorpha including Testudines, crocodilians, and birds (**c**). “num. species” denotes the number of species examined in each lineage. Presence or loss of *sox15* is shown as + or −. “unknown” indicates that the existence of *sox15* was not determined in this analysis

We previously reported that *Sox15* is a pseudogene in the marsupial opossum, although *Sox15* has a function in eutherian mammals [13, 14]. To examine whether pseudogenization of *Sox15* occurred in the common ancestor of marsupials, we searched for *Sox15* orthologs in seven other marsupial genomes using genome browser and tblastn analysis using the amino acid sequences encoded by eutherian *Sox15* (Additional file 1: Table S1) and performed synteny analysis using the *Fxr2* and *Mpdu1* genes adjacent to *Sox15*. Annotated orthologs of *Sox15* or *Sox15*-like sequences were identified between *Fxr2* and *Mpdu1* in the genomes of all seven species. The *Sox15*-like sequence of Tammar wallaby (*Macropus eugenii*) did not contain its HMG box-coding region, which was likely caused by sequencing gaps. In contrast, the *Sox15*-like sequences of both Tasmanian devil (*Sarcophilus harrisii*) and fairy possum (*Gymnobelideus leadbeateri*) contained pseudogenization signatures evident as in-frame stop codons and frame shift mutations in the HMG box-encoding regions (Figs. 1a, 2a, and Additional file 1: Fig. S1). However, the *Sox15*-like sequence of the other four species—common brushtail possum (*Trichosurus vulpecula*), thylacine (*Thylacinus cynocephalus*), koala (*Phascolarctos cinereus*), and wombat (*Vombatus ursinus*)—contained an intact 75 amino acid HMG box-encoding region (Figs. 1a, 2a, and Additional file 1: Fig. S1). Importantly, the following two observations indicated that the pseudogenizations of the *Sox15* orthologs must have arisen independently in the three distinct ancestors of Tasmanian devil, fairy possum, and opossum. First, the sequences adjacent to the in-frame stop codon and frameshift mutation sites differed from each other. Second, each order of marsupials, *Dasyuromorphia* (green in Fig. 2a) containing Tasmanian devil and thylacine and *Phalangeriformes* (yellow in Fig. 2a) containing the fairy possum and common brushtail possum, harbored both pseudogenized and non-pseudogenized *Sox15* genes (Fig. 2a). These results indicate the independent pseudogenizations of *Sox15* during marsupial divergence.

### Absence of pseudogenizations/losses of *Sox15* in both eutherian and monotreme mammals

We previously reported the potential neofunctionalization role of mouse *Sox15* in placental development [5, 13, 14]. In addition, Lee et al. [11] reported the involvement of *Sox15* in myogenesis. Based on our finding of *Sox15* pseudogenization during marsupial divergence, we searched for *Sox15* orthologs in NCBI gene databases from as many different eutherian mammalian genomes as possible. Annotation for *Sox15* was found in all the 148 genomes examined. Synteny of the *Sox15* orthologs in nine of 148 species is shown in Fig. 1a. All 148 *Sox15* sequences contained predicted open reading frames (ORFs). In addition, all the eutherian mammals, except polar bear (*Ursus maritimus*), harbored *Sox15* between *Mpdu1* and *Fxr2* (Fig. 1a). We discuss later whether the *Sox15* ortholog exists in *U. maritime.* To clarify the fate of *Sox15* in monotreme mammals, we also searched for *Sox15* in the platypus and echidna genomes and found *Sox15* annotations between *Mpdu1* and *Fxr2* (Fig. 1a).

### Independent gene losses of *sox15* during reptilian evolution

Independent pseudogenizations of *sox15* during marsupial evolution were identified (Fig. 1a). Next, we searched for *sox15* orthologs in reptilian lineages using the amino acid sequence encoded by *sox15* from Chinese softshell turtle (*Pelodiscus sinensis*) or the common wall lizard (*Podarcis muralis*). The search revealed the amino acid sequences of *sox15* and its adjacent *fxr2* and *mpdu1* genes as tblastn queries from 34 squamates (lizard and snake), 22 testudine (turtle and tortoise), four crocodilians, and 503 bird species (Additional file 1: Table S1).

In the 34 squamates genomes, the tblastn hits and synteny analysis identified *sox15* orthologs in 17 species from five different lineages, including Teiidae, Lacertidae, Agamidae, Viperidae, and Colubridae. No *sox15*-like sequences were found in the other 17 species (Fig. 2b and Additional file 1: Table S2). Blastn-based synteny analysis using Easyfig [17] indicated that *sox15* was lost in nine of the 17 species from four different lineages: Gekkonidae, Varanidae, Pythonidae, and Elapidae (Figs. 1b, 2b, Additional file 1: S3a–o and Table S2). We categorized the other eight as “unknown” because *fxr2* and/or *mpdu1* orthologs were not detected (Fig. 2b and Additional file 1: Table S2).

Among the 22 testudine species, *sox15* orthologs were identified in 17 species by the tblastn search and synteny analysis. There were no *sox15*-like sequences in the other five species (Fig. 2c and Additional file 1: Table S2). Synteny analysis with Easyfig indicated that the nine of the 11 species belonging to the Testudinoidea superfamily had *sox15*-like sequences between *fxr2* and *mpdu1* orthologs, whereas the Pinta Island giant tortoise (*Chelonoidis abingdonii*) had no *sox15*-like sequence between *fxr2* and *mpdu1* (Fig. 2c, Additional file 1: Fig. S3r–y, and Table S2). In the other four species, *sox15* was categorized as “unknown” because *fxr2* and/or *mpdu1* orthologs were not detected (Fig. 2c and Additional file 1: Table S2). In the four crocodilian genomes, no *sox15*-like sequences were found in the tblastn search (Fig. 2c, Additional file 1: Fig. S3p, q, and Table S2).

Synteny analysis with Easyfig indicated that *sox15* was lost in *Alligator sinensis* and *Crocodylus porosus* belonging to the Alligatoridae and Crocodylidae families, respectively (Fig. 1, Fig. 2c and Additional file 1: Fig. S3p, q). The other two species were categorized as “unknown”, because *fxr2* and/or *mpdu1* orthologs were not detected (Fig. 2c). Neither *sox15* nor *fxr2* orthologs were found in 503 bird genomes in the expected chromosomal regions (Fig. 2c and Additional file 1: Table S2), indicating that the sequences including the two genes must have been deleted in the ancestor of birds.

Collectively, these findings revealed independent gene losses of *sox15* at least twice during species diversity of squamates and crocodile-birds on reptile evolution.

### Absence of gene losses/pseudogenizations of *sox15* in the examined amphibian and actinopterygian fish genomes

Orthologous relationships have been described among mammalian *sox15*, teleost fish *sox19a/b*, and frog *soxD/sox15* [4]. Thus, we examined the fate of *sox15* orthologs in four amphibian species and 12 actinopterygian fish species. In amphibians, there were *sox15* orthologs between *fxr2* and *mpdu1* in the two caecilian species, *Geotrypetes seraphini* and* Rhinatrema bivittatum*, and between *ephb4* and *mpdu1* in the two frog species, *X. tropicalis* and *X. laevis* (Fig. 1c). In actinopterygian fishes, nine out of ten teleost species had *sox15* orthologs between *mpdu1* and *eif4a1*, and *Takifugu rubripes* ortholog of *sox15* was located on the same chromosome as *mpdu1* and *eif4a1* were (Fig. 1c). We also found *sox15* orthologs between *fxr2* and *mpdu1* in two nonteleost fish species, *Acipenser ruthenus* and *Erpetoichthys calabaricus*.All 16 genomes harbored *sox15* orthologs containing the predicted ORF (Fig. 1c).

### Absence of introns in cartilaginous fish *sox15*

Next, we examined the fate of *sox15/sox19* in cartilaginous fish (Chondrichthyes) and searched for *sox15* orthologs in eight elasmobranch and two holocephalan genomes (Additional file 1: Table S1) using the amino acid sequence of ropefish *Erpetoichthys calabaricus* SOX19 as a query. We found *sox15*-like sequences in three elasmobranch species (*Pristis pectinata*, *Chiloscyllium punctatum*, and *C. plagiosum*), but not in the two holocephalan species. The genome information of the other five elasmobranch species contained no *sox15-*like or adjacent *mpdu1*-like sequences. Synteny analysis of the elasmobranch *sox15*-like sequences based on FGNENESH gene annotation and phylogenetic tree reconstruction of *soxB1/G* ohnologs indicated that the *P. pectinata* and *C. plagiosum* sequences should correspond to *sox15* orthologs (Fig. 1c and Additional file 1: Fig. S4). In addition, the *C. punctatum* transcriptome database (https://transcriptome.riken.jp/squalomix/blast/) revealed that the *sox15* gene could be transcribed (Additional file 1: Fig. S5).

Notably, a sequence comparison between the *sox15* transcript and its genomic region in *C. punctatum* indicated that the *sox15* gene consists of one exon, although almost all the *sox15* orthologs in mammals, reptiles, amphibians, and actinopterygian fish examined in this study consisted of two exons with the same splicing sites. In contrast, no jawless fish orthologs of *sox15* were identified in the three species, *Petromyzon marinus*, *Lethenteron camtschaticum*, and *Eptatretus burgeri*.

### *sox15* has the highest *d*_N_/*d*_S_ value among the four ohnologs

**Fig. 3:**
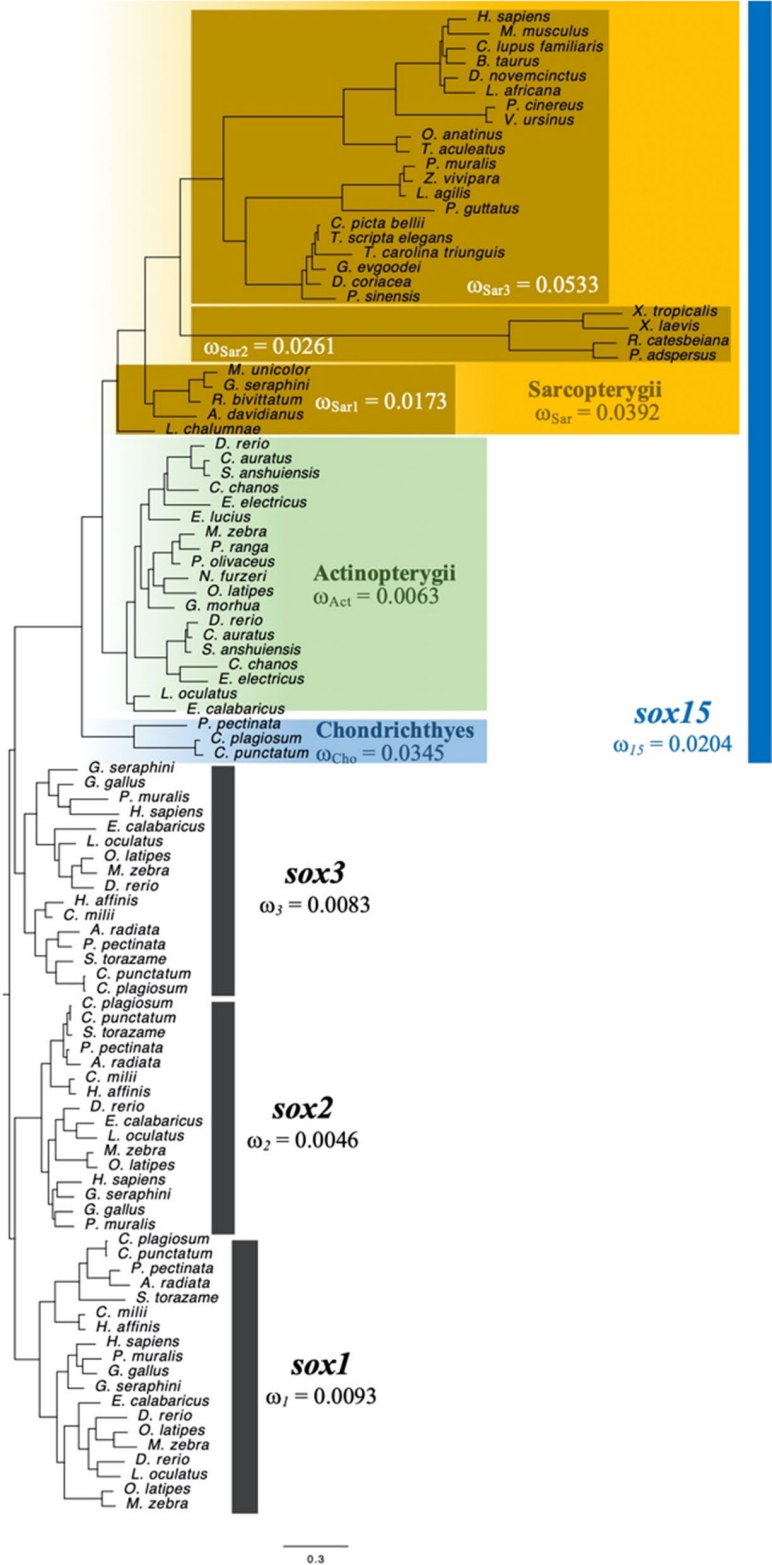
*d*_N_/*d*_S_ (ω) values of four *soxB1*/*G* ohnologs (*sox15* and *sox1-3 s*) during vertebrate evolution. A total of 102 gap-containing 930 nucleotide sequences corresponding to 310 codons were used for this tree inference. The GTR + F + R5 model was selected as the best-fit model in this dataset and used for the inference. *d*_N_/*d*_S_ (ω) values were calculated using 234 nucleotide sites with gaps deleted on the same nucleotide alignment as the tree inference. The scale bar indicates nucleotide substitutions per site

Independent pseudogenizations and losses of *sox15* were evident only in amniote divergence and not in anamniotes (Figs. 1, 2, and Additional file 1: Table S2). Additionally, *Sox15* could acquire new roles in skeletal muscle and placental development through neofunctionalization in eutherian diversification [4]. Why did drastic gene fates, including gene losses/pseudogenizations and neofunctionalization, occur in *sox15* during amniote divergence? To answer this question, we constructed phylogenetic trees of four *soxB1/G* ohnologous proteins—SOX1, SOX2, SOX3, and SOX15—in jawed vertebrates using maximum likelihood and Bayesian methods (Additional file 1: Fig. S4). Each tree showed a relatively longer branch length in the SOX15 clade than in SOX1, SOX2, or SOX3. Interestingly, longer branch lengths of SOX15 (SOX19) in amniotes and amphibians were observed compared to the lengths in actinopterygian fish and those of SOX1, SOX2, and SOX3 in jawed vertebrates. These findings suggest that there might be different selective pressures on the molecular history of *sox15* by evolutionary lineages in vertebrates, or in contrast to the other three ohnologs. To explore this idea we reconstructed a codon-based phylogenetic tree of jawed vertebrate *soxB1/G* ohnologs and calculated the ratio of nonsynonymous and synonymous substitution (*d*_N_/*d*_S_; ω) values for several vertebrate lineages (Fig. 3 and Additional file 1: Fig. S6). The ω values of *sox1*, *sox2*, *sox3*, and *sox15* in jawed vertebrates were 0.0093, 0.0046, 0.0083, and 0.0204, respectively (Fig. 3). Likelihood ratio tests revealed that the ω value of *sox15* significantly deviated from those of the other three *soxB1/G* ohnologs (*p* = 7.9 × 10^–4^, 1.7 × 10^–5^, and 4.9 × 10^–4^ for *sox1*, *sox2*, and *sox3*, respectively; Additional file 1: Table S4). We next divided jawed vertebrates into three classes—chondrichthyes (cartilaginous fishes), actinopterygii (bony fishes), and sarcopterygii (lobe-finned fish and tetrapod species), and calculated the ω value of *sox15*. Importantly, actinopterygian *sox15* had a lower ω value (0.0063) than that of chondrichthyes (0.0345) and sarcopterygii (0.0392), which was statistically supported by χ^2^ tests (*p* = 9.3 × 10^–15^ and 3.0 × 10^–15^, respectively; Additional file 1: Table S4 and Fig. 3). Because the sarcopterygian class had different evolutionary distances of *sox15* in the codon-based phylogenetic tree, we further divided the class into three groups—coelacanth and non-anuran amphibians (Sar1), anuran amphibians (Sar2), and amniotes (Sar3)—and examined each ω value of *sox15* from a node of sarcopterygian common ancestors. These results and χ^2^ test results revealed that the ω value of Sar3 was statistically significantly higher (0.0533) than those of Sar1 (0.0173) and Sar2 (0.0261) (*p* = 6.5 × 10^–4^ and 0.018, respectively; Additional file 1: Table S4 and Fig. 3). These findings indicate that the higher ω value of *sox15* than *sox1*, *sox2*, and *sox3* could be mainly affected by those of *sox15* in cartilaginous fishes and amniotes.

### Relaxed selection has participated in an asymmetric evolution of *sox15* among the four ohnologous *soxB1/G* members and is involved in pseudogenizations/losses of *sox15*

To elucidate whether the molecular evolution of *sox15* is under relaxed or intensified selection, we performed RELAX tests [18] of the gene in three classes of jawed vertebrates: chondrichthyes, actinopterygii, and sarcopterygii. *sox3* was used as a reference branch to calculate the relaxation or intensification parameter (k) of *sox15* in each class. The RELAX test data (Table 1) revealed that *sox15* might have evolved under relaxed selection in chondrichthyes and sarcopterygii (k = 0.25 [*p* = 3.1 × 10^–4^] and 0.40 [2.0 × 10^–4^], respectively), and even in all jawed vertebrates (k = 0.72, *p* = 8.0 × 10^–4^). Although the k value of actinopterygian *sox15* was also lower than 1 (k = 0.75), the χ^2^ test did not dismiss the null hypothesis of ‘k = 1’, suggesting that *sox15* and *sox3* have similarly evolved under purifying selection in bony fishes. These results indicate that the relaxed selection might have contributed to the higher ω value of *sox15* than that of *sox3* during cartilaginous fish and sarcopterygian evolution, and induced an asymmetric evolution of *sox15* among the four ohnologous *soxB1/G* members during jawed vertebrate evolution.

**Table 1:** RELAX tests for *sox15* in several lineages of jawed vertebrates

Lineage	Reference	Test	LRT	p value*	k**
Gnathostomata	*sox3*	*sox15*	11.25	8.0 × 10^-4^	0.72
Chondrichthyes	*sox3*	*sox15*	13.04	3.1 × 10^-4^	0.25
Actinopterygii	*sox3*	*sox15*	3.35	0.068	0.75
Sarcopterygii	*sox3*	*sox15*	13.91	2.0 × 10^-4^	0.40

*p values were calculated using χ^2^_1_ tests for each LRT. **k means a relaxation (k < 1) or intensification (k > 1) parameter. LRT, likelihood ratio test

## Discussion

**Fig. 4:**
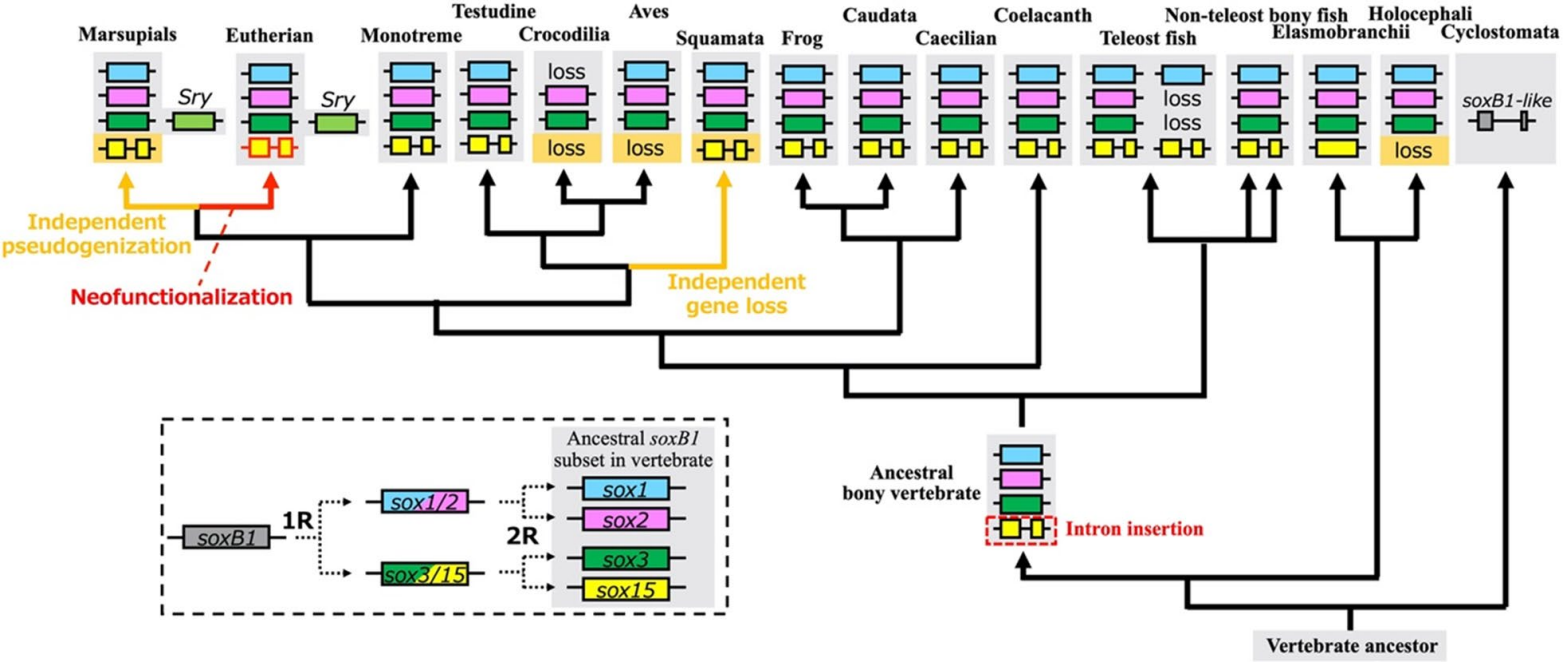
Proposed model for molecular evolution of *sox15* and its ohnologous members *sox1-3* s during vertebrate evolution

The three *soxB1* subfamily genes (*sox1, sox2,* and *sox3*) and *sox15* share ohnologous relationships in vertebrates, but only *sox15* orthologs do not belong to the *soxB1* subfamily [5]. In this study, we examined the fate of the unique ohnologous member *sox15* during vertebrate evolution. Figure 4 summarizes this study and represents proposed model for molecular evolution of *sox15* and its ohnologous members *sox1-3* s during vertebrate evolution. Although we found *sox15-*like sequences in the three jawless vertebrate genomes, we could not conclude that those are *sox15* orthologs by synteny and phylogenetic analyses. In contrast, within the cartilaginous fish, we identified *sox15* orthologs in three elasmobranchs, *P. pectinata*, *C. punctatum*, and *C. plagiosum*, but could not find orthologs to *sox15* or any of its flanking genes in two holocephalans, *Callorhinchus milii* and *Hydrolagus affinis.* It is possible that *sox15* and its adjacent genes were deleted in the ancestor of holocephalan fishes during cartilaginous fish evolution. Interestingly, we found an intron-free *sox15* in *C. punctatum* genomes (Figs. 1c,  4, and Additional file 1: Table S1). All other orthologs of *sox15* examined in this study consisted of two exons. Because vertebrate *sox1, sox2,* and *sox3* are single exon genes, Okuda et al. [4] reported that *sox15* should have acquired an intron during vertebrate evolution after or during the second round of WGD. Our results suggest that *sox15* acquired an intron in the ancestor of bony (Osteichthyes) fish after the divergence between cartilaginous and bony fish (Fig. 4). It will be interesting to clarify whether the spread of the intron-containing *sox15* within the ancestral population of bony fish might be neutrally or positively selected.

We previously reported the neofunctionalization and pseudogenization of *Sox15* in eutherian mice and marsupial opossums, respectively [5, 13, 14]. Our synteny analysis revealed that all the eutherian mammals examined except for the polar bear (*U. maritimus*), have *Sox15* between *Mpdu1* and *Fxr2* (Fig. 1a). We found only the exon 2 sequence of *Sox15* between *Mpdu1* and *Fxr2* in the *U. maritimus* genome database; there was a 1018 bp gap upstream of exon 2. It is conceivable that this gap could hide exon 1 of *Sox15.* These findings suggest the presence of *Sox15* for neofunctionalization in the common ancestor of eutherian mammals, resulting in no or almost no pseudogenizations/losses of *Sox15* during eutherian evolution (Fig. 4). Interestingly, *sox15* was independently pseudogenized during marsupial evolution and lost at least twice during reptile evolution (Figs. 1, 2, and 4). Our search revealed that the marsupial koala and wombat orthologs of *Sox15* were annotated among *Mpdu1* and *Fxr2*. These predicted amino acid sequences from the two orthologs comprising two exons and one intron shared high sequence identities with those of eutherian mice (Additional file 1: Fig. S2). In addition, their mRNA expression in both koala and wombat was confirmed by RNA-seq alignments, including 4 and 13 samples, respectively (https://www.ncbi.nlm.nih.gov/nuccore/XM_020966486.1; https://www.ncbi.nlm.nih.gov/nuccore/XM_027857251.1). Importantly, the koala and wombat *sox15* mRNAs have ORFs, encoding putative 228 and 216 amino acid sequences, respectively, which could function as SOX15. In contrast, there have been no reports of losses/pseudogenizations of *sox1, sox2,* or *sox3*. Why did only *sox15* orthologs have drastic gene fates during amniote divergence among the four ohnologs? The selection analysis revealed the highest *d*_N_/*d*_S_ value of *sox15* among the four ohnologs during jawed vertebrate evolution. In addition, the amniote *d*_N_/*d*_S_ value of *sox15* was the highest among the five classes (chondrichthyes, actinopterygii and Sar1-3) in jawed vertebrates (Fig. 3). These findings suggest that the relatively high *d*_N_/*d*_S_ value could be connected to the divergent gene fates of *sox15* during amniote divergence.

What is the difference in genetic backgrounds between independent pseudogenization and gene losses of *sox15* in marsupials and reptiles? Some inversions and deletions appeared to happen on the chromosomal regions around *sox15* during reptile-bird evolution (Fig. 1b, Additional file 1: Fig. S2), while there were almost no chromosomal inversions around *sox15* in mammals, including marsupials (Fig. 1a). It is possible that the deletions or inversions followed by deletions on the chromosomal region around *sox15* happened to result in independent losses of *sox15* during reptile diversification.

We could not identify any jawless fish orthologs of *sox15,* but found a *soxB1*-like gene in the NCBI gene database of sea lamprey (*P. marinus*) (Gene ID: 116956344). In addition, there was no synteny between this gene and any *soxB1/G* member, and the predicted amino acid sequence from the gene did not belong to any clade of SOX1, SOX2, SOX3, or SOX15 in jawed vertebrates (Additional file 1: Fig. S4). Because the common ancestor of agnathans underwent genome duplication [19], it would be interesting to investigate why only one *soxB1* gene remained in sea lamprey and how the gene evolved.

In general, ohnologs exhibit shared expression and functional redundancy prior to genome duplication, even in allotetraploidization by hybridization between two related species, as suggested by findings from the frog *Xenopus laevis* [20]. An additional WGD (3R-WGD) also occurred in the teleost ancestor [21]. Seebrafish *sox19a* and *sox19b*, which are co-orthologous to *sox15*, are presumptually derived from 3R-WGD [4]. We confirmed two *sox19* genes in three teleost species including zebrafish (Fig. 1c). However, we could not find one of two presumptive* sox19* genes or any of its flanking genes in other five teleost fish. Then why did two copies of *sox19* remain in the three teleost fish? *sox1, sox2*, *sox3,* and *sox15* were predominantly expressed in the developing CNS in non-amniotes [4, 9, 21–23]. Zebrafish *sox19a* and *sox19b* also showed specific expression in the developing CNS [4, 10]. It is possible that expression redundancy in the CNS has been involved in the retention of the *soxB1/G* members including teleost *sox19a* and *sox19b*.

It is believed that cis-element evolution resulted in the differentiation of expression patterns between ohnologs [24]. Mutations in the cis-regulatory elements for the CNS expression of *sox15* might cause the gene to escape from the spatio-temporal expression regime the other three ohnologs have been subjected to. Then molecular evolution of *sox15* under slightly relaxed purifying selection during amniote diversification might be involved in the divergent gene fates of *sox15*, neofunctionalization in the ancestor of eutherian mammals, pseudogenization during marsupial diversification, and losses during reptile diversification. It has been proposed that the fate of duplicated genes could be affected by the intrinsic properties of genomic regions harboring them as well as functional constraints on their roles [25]. Then another possibility is that the instability of the genomic region during amniote evolution has caused relatively high mutation rates of both the cis-regulatory elements and the coding sequence of *sox15*, resulting in the drastic gene fates.

## Conclusions

The evolutionary analyses revealed independent pseudogenizations and losses of *sox15* during marsupial mammalian and reptile evolution, respectively, although no pseudogenizations/losses of *sox15* were observed in the other classes of jawed vertebrates. *sox15* showed the highest *d*_N_/*d*_S_ value among the four ohnologous *soxB1/G* members, and higher *d*_N_/*d*_S_ values in marsupials and reptiles than in eutherians. Moreover, we found that relaxed selection has been involved in asymmetric evolution of *sox15* among the four ohnologs, which might have been one of the factors for the *sox15*’s drastic gene fates including pseudogenizations, losses, and neofuctionalization during amniote diversification. We propose that *sox15* might have been released from some evolutionary constraints including expression and/or functional redundancy in the CNS, unlike *sox1, sox2*, and *sox3* in the common ancestor of amniotes.

## Methods

### Identification of *sox15* orthologs from genome databases

Genome sequences from 571 vertebrate species were downloaded in FASTA format from NCBI (https://www.ncbi.nlm.nih.gov/). Local databases were created using BLAST (v.2.9.0 + ; https://blast.ncbi.nlm.nih.gov/Blast.cgi?CMD=Web&PAGE_TYPE=BlastD ocs&DOC_TYPE = Download). Gene searches using tblastn (https://blast.ncbi.nlm.nih.gov/Blast.cgi?PROGRAM=tblastn&PAGE_TYPE=BlastSearch&LINK_LOC=blasthome) [26] were performed using the default option (task = tblastn; evalue = 10; db_gencode = 1; max_intron_length = 0; matrix = BLOSUM62; comp_based_stats = 2). For marsupials, XP_020822145.1 (*Phascolarctos cinereus* SOX15), XP_020822143.1 (*Phascolarctos cinereus* MPDU1), and XP_020822141.1 (*Phascolarctos cinereus* FXR2) were used for query amino acid sequences. For squamates, XP_028559641.1 (*Podarcis muralis* SOX15), XP_028558914.1 (*Podarcis muralis* MPDU1), and XP_028557780.1 (*Podarcis muralis* FXR2) were used for query amino acid sequences. For Testudines, XP_014432453.1 (*Pelodiscus sinensis* SOX15), XP_025043921.1 (*Pelodiscus sinensis* MPDU1), and XP_006112154.1 (*Pelodiscus sinensis* FXR2) were used for query amino acid sequences. For crocodilians, XP_014432453.1 (*Pelodiscus sinensis* SOX15), XP_025049549.1 (*Alligator sinensis* MPDU1), and XP_025049532.1 (*Alligator sinensis* FXR2) were used for query amino acid sequences. For birds, XP_014432453.1 (*Pelodiscus sinensis* SOX15), XP_032940188.1 (*Catharus ustulatus* MPDU1), and XP_025049532.1 (*Alligator sinensis* FXR2) were used for query amino acid sequences. For Chondrichthyes, XP_028654266.1 (*Erpetoichthys calabaricus* SOX19), XP_028654265.1 (*Erpetoichthys calabaricus* MPDU1), and XP_028654267.1 (*Erpetoichthys calabaricus* FXR2) were used for query amino acid sequences. Sequence similarity of the top hit sequence to the SOX15 sequence in the database of the most similar species was analyzed using megablast (https://blast.ncbi.nlm.nih.gov/Blast.cgi?PROGRAM=blastn&PAGE_TYPE=BlastSearch&LINK_LOC=blasthome) using BLAST, followed by manual synteny analysis. Gene annotation of *sox15* orthologs was performed with Softberry-FGENESH (http://www.softberry.com/berry.phtml?topic=case_study_animal&no_menu=on) [27] based on a species-specific gene-finding parameter [28, 29].

### Molecular phylogenetic analysis

The coding sequences of *sox1, sox2, sox3*, and *sox15* in various vertebrate species were translated to amino acid sequences. Multiple alignment of results was performed using MAFFT version 7.427 (https://mafft.cbrc.jp/alignment/software/) [30]. Multiple alignments of nucleotide sequences were deduced using Pal2nal version 14 (http://www.bork.embl.de/pal2nal) [31] with the amino acid alignments. Ambiguous sites of the alignments were removed using trimAl version 1.2 (http://trimal.cgenomics.org/) [32] with option-gappyout. Maximum likelihood trees were inferred by IQ-TREE (http://www.iqtree.org/) [33], where the most fitting amino acid and nucleotide substitution rate was estimated by ModelFinder in part of analysis by IQ-TREE [34]. A Bayesian phylogenetic tree was inferred by Mrbayes 3.2.7a (https://nbisweden.github.io/MrBayes/download.html) [35]. Two MCMC chains were run 300,000 times and sampled 100 times to analyze the convergence of the statistics by Tracer version 1.7.1 (http://beast.community/tracer). *d*_N_/*d*_S_ was estimated by codeml in Paml4.8 (http://abacus.gene.ucl.ac.uk/software/paml.html) [36]. A branch model was used to calculate the *d*_N_/*d*_S_ ratio for each group. RELAX [37] in HYPHY version 2.5.8 (https://github.com/veg/hyphy) was used to detect the relaxed or intensified selection for the *sox15* ortholog in each lineage.

### Statistical analysis

χ^2^ tests were performed using Microsoft Excel for Mac version 16.42 for LRT value in codeml and RELAX analysis.

## Supplementary Information

**Additional file 1: Table S1.** Genome IDs used in the study. **Table S2.** The presence ( +) and absence (-) of *sox15*, *mpdu1*, and *fxr2* on reptile genomes used in Fig. 2a, b. **Table S3.** Genbank numbers and range of the genes used in the analyses. **Table S4.** Likelihood ratio test for ω values in Fig. 4. **Figure S1.** Multiple alignment of the HMG box-encoding nucleotide sequences of marsupial pseudogenized and non-pseudogenized. **Figure S2.** Predicted amino acid sequences of marsupial SOX15 from koala (*Phascolarctos cinereus*) and wombat (*Vombatus ursinus*). **Figure S3.** Easyfig analysis for the locus encoding *fxr2* and *mpdu1* genes in reptiles. **Figure S4.** Phylogenetic relationships of vertebrate *soxB1*/*G* ohnologous proteins. (a) Maximum likelihood and (b) Bayesian phylogenetic trees were shown. A total of 108 aa sequences containing 273 sites were used for this tree inference. The JTT + F + I + Γ4 model was selected as the best-fit model in this dataset and used for the inference. The invertebrate SOXB1 clade was rooted. Values of (a) the 1000 times ultrafast bootstrap test and (b) the Bayesian posterior probability are shown at each node. Only the 90 ≦ bootstrap values and 0.90 ≦ posterior probabilities were shown. The scale bars indicate aa substitutions per site. **Figure S5.** An intron-less structure of *Chiloscyllium punctatum* ortholog of *sox15* by blastn hit.

## Data Availability

All data generated or analyzed during this study are included in the main manuscript, figures, tables, and supplementary information file. Raw data of phylogenetic tree inference and analyses of codeml and RELAX are available in the figshare repository, https://doi.org/10.6084/m9.figshare.14400617.v1.

## Abbreviations

CNS: Central nervous system
Sry: Sex-determining region Y
HMG: High mobility group
*Sox*: *Sex-determining region Y* (*Sry*)-Type high mobility group (HMG) box
2R-WGD: Two rounds of whole-genome duplication
*d*_N_/*d*_S_: Ratio of nonsynonymous substitution sites per synonymous substitution site
LRTs: Likelihood ratio tests
