# Supplementary material for: Independent pseudogenizations and losses of sox15 during amniote diversification following asymmetric ohnolog evolution
Source: BMC Ecol Evol. 2021 Jun 30;21:134. doi: 10.1186/s12862-021-01864-z (PMC8244163; doi:10.1186/s12862-021-01864-z)
Supplement: Supplementary file 1 — Additional file 1: Table S1. Genome IDs used in the study. Table S2. The presence ( +) and absence (-) of sox15, mpdu1, and fxr2 on reptile genomes used in Fig. 2a, b. Table S3. Genbank numbers and range of the genes used in the analyses. Table S4. Likelihood ratio test for ω values in Fig. 4. Figure S1. Multiple alignment of the HMG box-encoding nucleotide sequences of marsupial pseudogenized and non-pseudogenized. Figure S2. Predicted amino acid sequences of marsupial SOX15 from koala (Phascolarctos cinereus) and wombat (Vombatus ursinus). Figure S3. Easyfig analysis for the locus encoding fxr2 and mpdu1 genes in reptiles. Figure S4. Phylogenetic relationships of vertebrate soxB1/G ohnologous proteins. (a) Maximum likelihood and (b) Bayesian phylogenetic trees were shown. A total of 108 aa sequences containing 273 sites were used for this tree inference. The JTT + F + I + Γ4 model was selected as the best-fit model in this dataset and used for the inference. The invertebrate SOXB1 clade was rooted. Values of (a) the 1000 times ultrafast bootstrap test and (b) the Bayesian posterior probability are shown at each node. Only the 90 ≦ bootstrap values and 0.90 ≦ posterior probabilities were shown. The scale bars indicate aa substitutions per site. Figure S5. An intron-less structure of Chiloscyllium punctatum ortholog of sox15 by blastn hit. [file 12862_2021_1864_MOESM1_ESM.docx]

**Table S1. Genome IDs used in the study**

| Phylogeny | Species | Assembly | locus located by sox15 orthologs and/or their flanking genes |
| --- | --- | --- | --- |
| Anura | *Leptobrachium leishanense* | GCA_009667805.1 | Contig00012:105880-105656 (*sox15*) |
| Anura | *Limnodynastes dumerilii* | GCA_011038615.1 | Not detected |
| Anura | *Lithobates catesbeianus (Rana catesbeiana)* | GCA_002284835.2 | scaffold Rc-01r160223s0013348:1174-4302 (*sox15*) |
| Anura | *Nanorana parkeri* | GCF_000935625.1 | scaffold3440:47895-47671 (*sox15*) |
| Anura | *Oophaga pumilio* | GCA_009801035.1 | Not detected |
| Anura | *Pyxicephalus adspersus* | GCA_004786255.1 | chr2:1996676-1999089 (*sox15*) |
| Anura | *Rana temporaria* | GCA_009802015.1 | chr3:485190019-485190243 (*sox15*) |
| Anura | *Rhinella marina* | GCA_900303285.1 | Not detected |
| Anura | *Scaphiopus couchii* | GCA_009364435.1 | Not detected |
| Anura | *Scaphiopus holbrookii* | GCA_009364455.1 | Not detected |
| Anura | *Spea bombifrons* | GCA_009364475.1 | scaffold 5525487:369-145 (*sox15*) |
| Anura | *Spea multiplicata* | GCA_009364415.1 | scaff00039177_ctg7180000218279:4286-4062 (*sox15*) |
| Anura | *Xenopus laevis* | GCF_001663975.1 | chr3L:142714941-142717953 (*sox15*) |
| Anura | *Xenopus tropicalis* | GCF_000004195.4 | chr3:152467110-152500459 (*sox15*) |
| Aves | *Gallus gallus* | GCF_000002315.6 | Not detected |
| Aves | *Meleagris gallopavo* | GCF_000146605.3 | Not detected |
| Aves | *Coturnix japonica* | GCF_001577835.2 | Not detected |
| Aves | *Eudromia elegans* | GCA_013398775.1 | Not detected |
| Aves | *Apteryx haastii* | GCA_003342985.1 | Not detected |
| Aves | *Pterocnemia pennata* | GCA_003342835.1 | Not detected |
| Aves | *Arenaria interpres* | GCA_013399435.1 | Not detected |
| Aves | *Struthio camelus* | GCF_000698965.1 | Not detected |
| Aves | *Dromaius novaehollandiae* | GCF_003342905.1 | Not detected |
| Aves | *Anomalopteryx didiformis* | GCA_006937325.1 | Not detected |
| Aves | *Casuarius casuarius* | GCA_013396415.1 | Not detected |
| Aves | *Ciconia boyciana* | GCA_002002965.1 | scaffold Cibo_231016: 328-402 (*mpdu1*) |
| Aves | *Vidua chalybeata* | GCA_013398495.1 | Not detected |
| Aves | *Falco peregrinus* | GCF_000337955.1 | Not detected |
| Aves | *Rhea americana* | GCA_003343005.1 | Not detected |
| Aves | *Taeniopygia guttata* | GCF_008822105.2 | Not detected |
| Aves | *Anas platyrhynchos* | GCF_003850225.1 | Not detected |
| Aves | *Eudyptula minor* | GCA_010080355.1 | Not detected |
| Aves | *Anseranas semipalmata* | GCA_013399115.1 | Not detected |
| Aves | *Branta canadensis* | GCA_006130075.1 | Not detected |
| Aves | *Gavia stellata* | GCF_000690875.1 | Not detected |
| Aves | *Alectura lathami* | GCA_013399715.1 | Not detected |
| Aves | *Nisaetus nipalensis* | GCA_012487455.1 | Not detected |
| Aves | *Cathartes aura* | GCA_000699945.1 | Not detected |
| Aves | *Menura novaehollandiae* | GCA_013396355.1 | Not detected |
| Aves | *Eudyptes chrysocome* | GCA_010085355.1 | Not detected |
| Aves | *Nipponia nippon* | GCF_000708225.1 | Not detected |
| Aves | *Podiceps cristatus* | GCA_000699545.1 | Not detected |
| Aves | *Pandion haliaetus* | GCA_013401275.1 | Not detected |
| Aves | *Rhynochetos jubatus* | GCA_013398095.1 | Not detected |
| Aves | *Sylvia atricapilla* | GCA_009819655.1 | Not detected |
| Aves | *Cairina moschata* | GCA_009194515.1 | Not detected |
| Aves | *Falco tinnunculus* | GCA_010332995.1 | Not detected |
| Aves | *Agapornis roseicollis* | GCA_002631895.1 | Not detected |
| Aves | *Geococcyx californianus* | GCA_013389885.1 | Not detected |
| Aves | *Bambusicola thoracicus* | GCA_002909625.1 | Not detected |
| Aves | *Accipiter gentilis* | GCA_012487025.1 | Not detected |
| Aves | *Gallirallus okinawae* | GCA_002003005.1 | Not detected |
| Aves | *Cygnus atratus* | GCF_013377495.1 | Not detected |
| Aves | *Apteryx owenii* | GCA_003342965.1 | Not detected |
| Aves | *Nymphicus hollandicus* | GCA_009823435.1 | Not detected |
| Aves | *Phasianus colchicus* | GCF_004143745.1 | scaffold26768_cov28:1172-1282 (*mpdu1*) |
| Aves | *Columba livia* | GCF_000337935.1 | Not detected |
| Aves | *Chrysolophus pictus* | GCA_003413605.1 | Not detected |
| Aves | *Melopsittacus undulatus* | GCF_012275295.1 | chr25:212365-212484 (*mpdu1*) |
| Aves | *Leiothrix lutea* | GCA_013400445.1 | Not detected |
| Aves | *Serinus canaria* | GCF_007115625.1 | Not detected |
| Aves | *Ficedula albicollis* | GCF_000247815.1 | Not detected |
| Aves | *Anhinga rufa* | GCA_013400875.1 | Not detected |
| Aves | *Paradoxornis webbianus* | GCA_013397395.1 | Not detected |
| Aves | *Colinus virginianus* | GCA_008692595.1 | Not detected |
| Aves | *Parus major* | GCF_001522545.3 | Not detected |
| Aves | *Aptenodytes patagonicus* | GCA_010087175.1 | Not detected |
| Aves | *Geospiza fortis* | GCF_000277835.1 | scaffold1133:21141-23286(*mpdu1*) |
| Aves | *Junco hyemalis* | GCA_003829775.1 | Not detected |
| Aves | *Numida meleagris* | GCF_002078875.1 | Not detected |
| Aves | *Falco cherrug* | GCF_000337975.1 | Not detected |
| Aves | *Phylloscopus trochilus* | GCA_002305835.1 | Not detected |
| Aves | *Pseudopodoces humilis* | GCF_000331425.1 | scaffold scaffold184:3623-7919 (*mpdu1*) |
| Aves | *Amazona vittata* | GCA_000332375.2 | Not detected |
| Aves | *Agelaius phoeniceus* | GCA_013398535.1 | Not detected |
| Aves | *Eopsaltria australis* | GCA_003426825.1 | Not detected |
| Aves | *Alauda arvensis* | GCA_902810485.1 | Not detected |
| Aves | *Grus nigricollis* | GCA_004360235.1 | Not detected |
| Aves | *Grus monacha* | GCA_012487855.1 | Not detected |
| Aves | *Grus japonensis* | GCA_002002985.1 | Not detected |
| Aves | *Tachycineta bicolor* | GCA_007636935.1 | Not detected |
| Aves | *Grus americana* | GCA_013390085.1 | Not detected |
| Aves | *Balearica regulorum* | GCF_000709895.1 | Not detected |
| Aves | *Zosterops borbonicus* | GCA_007252995.1 | Not detected |
| Aves | *Zonotrichia albicollis* | GCF_000385455.1 | Not detected |
| Aves | *Pygoscelis adeliae* | GCF_000699105.1 | Not detected |
| Aves | *Passer domesticus* | GCA_001700915.1 | Not detected |
| Aves | *Ara macao* | GCA_000400695.1 | Not detected |
| Aves | *Pygoscelis antarcticus* | GCA_010078415.1 | Not detected |
| Aves | *Corvus cornix* | GCF_000738735.2 | Not detected |
| Aves | *Spheniscus demersus* | GCA_010077935.1 | Not detected |
| Aves | *Sagittarius serpentarius* | GCA_013399415.1 | Not detected |
| Aves | *Lyrurus tetrix* | GCA_000586395.1 | Not detected |
| Aves | *Anser cygnoides* | GCF_000971095.1 | Not detected |
| Aves | *Egretta garzetta* | GCF_000687185.1 | Not detected |
| Aves | *Jacana jacana* | GCA_013400555.1 | Not detected |
| Aves | *Phoenicopterus ruber* | GCA_009819775.1 | Not detected |
| Aves | *Phaethon lepturus* | GCF_000687285.1 | Not detected |
| Aves | *Pelecanus crispus* | GCF_000687375.1 | Not detected |
| Aves | *Cariama cristata* | GCF_000690535.1 | Not detected |
| Aves | *Colius striatus* | GCF_000690715.1 | Not detected |
| Aves | *Eurypyga helias* | GCF_000690775.1 | Not detected |
| Aves | *Fulmarus glacialis* | GCF_000690835.1 | Not detected |
| Aves | *Haliaeetus albicilla* | GCF_000691405.1 | Not detected |
| Aves | *Merops nubicus* | GCF_000691845.1 | Not detected |
| Aves | *Manacus vitellinus* | GCF_001715985.3 | Not detected |
| Aves | *Opisthocomus hoazin* | GCF_000692075.1 | Not detected |
| Aves | *Leptosomus discolor* | GCF_000691785.1 | Not detected |
| Aves | *Acanthisitta chloris* | GCF_000695815.1 | Not detected |
| Aves | *Aquila chrysaetos* | GCF_900496995.1 | Not detected |
| Aves | *Mesitornis unicolor* | GCF_000695765.1 | Not detected |
| Aves | *Corvus brachyrhynchos* | GCF_000691975.1 | Not detected |
| Aves | *Chlamydotis undulata* | GCA_003400225.1 | Not detected |
| Aves | *Picoides pubescens* | GCF_000699005.1 | Not detected |
| Aves | *Calypte anna* | GCF_003957555.1 | Not detected |
| Aves | *Aptenodytes forsteri* | GCF_000699145.1 | Not detected |
| Aves | *Pterocles gutturalis* | GCF_000699245.1 | Not detected |
| Aves | *Antrostomus carolinensis* | GCF_000700745.1 | Not detected |
| Aves | *Charadrius vociferus* | GCF_000708025.1 | Not detected |
| Aves | *Apaloderma vittatum* | GCF_000703405.1 | Not detected |
| Aves | *Cuculus canorus* | GCF_000709325.1 | Not detected |
| Aves | *Phalacrocorax carbo* | GCF_000708925.1 | Not detected |
| Aves | *Tauraco erythrolophus* | GCF_000709365.1 | Not detected |
| Aves | *Tinamus guttatus* | GCF_000705375.1 | Not detected |
| Aves | *Buceros rhinoceros* | GCF_000710305.1 | Not detected |
| Aves | *Haliaeetus leucocephalus* | GCF_000737465.1 | Not detected |
| Aves | *Nestor notabilis* | GCF_000696875.1 | scaffold656:4860-4979 (*mpdu1*) |
| Aves | *Chaetura pelagica* | GCF_000747805.1 | Not detected |
| Aves | *Bubo bubo* | GCA_010303855.1 | Not detected |
| Aves | *Pavo cristatus* | GCA_005519975.1 | Not detected |
| Aves | *Aythya fuligula* | GCF_009819795.1 | Not detected |
| Aves | *Passer montanus* | GCA_014805655.1 | scaffold18929:135-201 (*mpdu1*) |
| Aves | *Poecile atricapillus* | GCA_011421415.1 | Not detected |
| Aves | *Chlamydotis macqueenii* | GCF_000695195.1 | Not detected |
| Aves | *Fringilla coelebs* | GCA_015532645.1 | Not detected |
| Aves | *Cardinalis cardinalis* | GCA_014549065.1 | Not detected |
| Aves | *Fulica atra* | GCA_013372525.1 | Not detected |
| Aves | *Anser indicus* | GCA_006229135.1 | Not detected |
| Aves | *Loxia curvirostra* | GCA_013398455.1 | Not detected |
| Aves | *Heliornis fulica* | GCA_013399135.1 | Not detected |
| Aves | *Accipiter nisus* | GCA_004320145.1 | Not detected |
| Aves | *Ara glaucogularis* | GCA_013433245.1 | Not detected |
| Aves | *Aratinga solstitialis* | GCA_902168055.1 | Not detected |
| Aves | *Guaruba guarouba* | GCA_011800095.1 | Not detected |
| Aves | *Phoebastria albatrus* | GCA_014363385.1 | Not detected |
| Aves | *Corvus hawaiiensis* | GCA_003402825.1 | Not detected |
| Aves | *Cygnus cygnus* | GCA_014362685.1 | Not detected |
| Aves | *Cygnus olor* | GCA_009769625.1 | Not detected |
| Aves | *Corvus macrorhynchos* | GCA_014363025.1 | Not detected |
| Aves | *Recurvirostra avosetta* | GCA_004023745.1 | Not detected |
| Aves | *Oreotrochilus melanogaster* | GCA_013400995.1 | Not detected |
| Aves | *Calidris pygmaea* | GCA_003697955.1 | Not detected |
| Aves | *Zosterops lateralis* | GCA_001281735.1 | Not detected |
| Aves | *Psittacus erithacus* | GCA_009867235.1 | Not detected |
| Aves | *Ara militaris* | GCA_010015965.1 | Not detected |
| Aves | Myiopsitta monachus | GCA_013036005.1 | Not detected |
| Aves | *Eclectus roratus* | GCA_011763535.1 | Not detected |
| Aves | *Amazona aestiva* | GCA_001420675.1 | Not detected |
| Aves | *Upupa epops* | GCA_013397515.1 | Not detected |
| Aves | *Turdus rufiventris* | GCA_013186435.1 | Not detected |
| Aves | *Calidris pugnax* | GCF_001431845.1 | Not detected |
| Aves | *Sturnus vulgaris* | GCF_001447265.1 | Not detected |
| Aves | *Motacilla alba* | GCF_902150015.1 | Not detected |
| Aves | *Notiomystis cincta* | GCA_013398075.1 | Not detected |
| Aves | *Lonchura striata* | GCF_005870125.1 | scaffold ScxkALA_1814:405598-405711 (*mpdu1*) |
| Aves | *Ara ararauna* | GCA_010014805.1 | Not detected |
| Aves | *Callipepla squamata* | GCA_002218305.1 | Not detected |
| Aves | *Falco rusticolus* | GCF_015220075.1 | Not detected |
| Aves | *Pomatorhinus ruficollis* | GCA_013401195.1 | Not detected |
| Aves | *Nannopterum brasilianus* | GCA_002174335.1 | Not detected |
| Aves | *Lepidothrix coronata* | GCF_001604755.1 | Not detected |
| Aves | *Phylloscopus trochiloides* | GCA_001655095.1 | Not detected |
| Aves | *Phylloscopus plumbeitarsus* | GCA_001655115.1 | Not detected |
| Aves | *Cyanoderma ruficeps* | GCA_008694505.1 | Not detected |
| Aves | *Setophaga coronata* | GCA_001746935.2 | Not detected |
| Aves | *Tympanuchus cupido* | GCA_001870855.1 | Not detected |
| Aves | *Caloenas nicobarica* | GCA_013398195.1 | Not detected |
| Aves | *Emberiza fucata* | GCA_013398835.1 | Not detected |
| Aves | *Lagopus muta* | GCA_004320205.1 | Not detected |
| Aves | *Patagioenas fasciata* | GCA_002029285.1 | Not detected |
| Aves | *Sporophila hypoxantha* | GCA_002167245.1 | Not detected |
| Aves | *Nannopterum harrisi* | GCA_002173475.1 | Not detected |
| Aves | *Nannopterum auritus* | GCA_002173455.1 | Not detected |
| Aves | *Himantopus himantopus* | GCA_003993805.1 | Not detected |
| Aves | *Anas zonorhyncha* | GCA_002224875.1 | Not detected |
| Aves | *Prunella fulvescens* | GCA_013400715.1 | scaffold34560:656-778 (*mpdu1*) |
| Aves | *Uria lomvia* | GCA_002289315.1 | Not detected |
| Aves | *Strix occidentalis* | GCA_002372975.1 | Not detected |
| Aves | *Saxicola maurus* | GCA_900205225.1 | contig xfSc0000225:56096-56173 (*mpdu1*) |
| Aves | *Anser brachyrhynchus* | GCA_002592135.1 | Not detected |
| Aves | *Spheniscus magellanicus* | GCA_010076225.1 | Not detected |
| Aves | *Spheniscus humboldti* | GCA_010076325.1 | Not detected |
| Aves | *Spheniscus mendiculus* | GCA_003264655.1 | Not detected |
| Aves | *Sterna hirundo* | GCA_009819605.1 | Not detected |
| Aves | *Limosa lapponica* | GCA_002844005.1 | Not detected |
| Aves | *Acridotheres javanicus* | GCA_002849675.1 | Not detected |
| Aves | *Psittacula krameri* | GCA_002870145.1 | Not detected |
| Aves | *Cyanistes caeruleus* | GCF_002901205.1 | scaffold14519_part1:201-341 (*mpdu1*) |
| Aves | *Empidonax traillii* | GCF_003031625.1 | Not detected |
| Aves | *Athene cunicularia* | GCF_003259725.1 | Not detected |
| Aves | *Pygoscelis papua* | GCA_003264615.1 | Not detected |
| Aves | *Hemignathus virens* | GCA_003286495.1 | Not detected |
| Aves | *Nothoprocta perdicaria* | GCF_003342845.1 | Not detected |
| Aves | *Apteryx rowi* | GCF_003343035.1 | Not detected |
| Aves | *Crypturellus cinnamomeus* | GCA_003342915.1 | Not detected |
| Aves | *Syrmaticus mikado* | GCA_003435085.1 | Not detected |
| Aves | *Mixornis gularis* | GCA_003546035.1 | Not detected |
| Aves | *Erythrura gouldiae* | GCA_003676055.1 | Not detected |
| Aves | *Hirundo rustica* | GCA_015227805.1 | Not detected |
| Aves | *Paradisaea rubra* | GCA_003713215.1 | Not detected |
| Aves | *Diphyllodes magnificus* | GCA_003713285.1 | Not detected |
| Aves | *Parotia lawesii* | GCA_003713295.1 | Not detected |
| Aves | *Cicinnurus regius* | GCA_003713305.1 | Not detected |
| Aves | *Paradisaea raggiana* | GCA_003713265.1 | Not detected |
| Aves | *Pipra filicauda* | GCF_003945595.1 | Contig386_pilon:3661-7104 (*mpdu1*) |
| Aves | *Corapipo altera* | GCF_003945725.1 | Not detected |
| Aves | *Amazona collaria* | GCA_003947215.1 | Not detected |
| Aves | *Neopelma chrysocephalum* | GCF_003984885.1 | Not detected |
| Aves | *Strigops habroptila* | GCF_004027225.2 | scaffold NW_022045622.1_ctg1:31017-35092 (*mpdu1*) |
| Aves | *Scolopax mira* | GCA_004320125.1 | scaffold Scmi_19511:109-174 (*mpdu1*) |
| Aves | *Dendrocopos noguchii* | GCA_004320165.1 | Not detected |
| Aves | *Bubo blakistoni* | GCA_004320225.1 | Not detected |
| Aves | *Indicator maculatus* | GCA_013399975.1 | scaffold13737:9-161(*mpdu1*) |
| Aves | *Atlantisia rogersi* | GCA_013401215.1 | Not detected |
| Aves | *Eolophus roseicapillus* | GCA_013397615.1 | Not detected |
| Aves | *Rhagologus leucostigma* | GCA_013397725.1 | Not detected |
| Aves | *Charadrius alexandrinus* | GCA_008711295.1 | Not detected |
| Aves | *Otus sunia* | GCA_010365825.1 | Not detected |
| Aves | *Streptopelia turtur* | GCA_901699155.1 | Not detected |
| Aves | *Centrocercus minimus* | GCA_005890655.1 | Not detected |
| Aves | *Zosterops pallidus* | GCA_007556475.1 | Not detected |
| Aves | *Pseudorectes ferrugineus* | GCA_008033365.1 | Not detected |
| Aves | *Camarhynchus parvulus* | GCF_901933205.1 | scaffold STF_HiC:95380-101309 (*mpdu1*) |
| Aves | *Lichenostomus cassidix* | GCA_008360975.1 | Not detected |
| Aves | *Alca torda* | GCA_008658365.1 | Not detected |
| Aves | *Burhinus oedicnemus* | GCA_008921705.1 | Not detected |
| Aves | *Anthochaera phrygia* | GCA_009430485.1 | Not detected |
| Aves | *Corvus moneduloides* | GCF_009650955.1 | scaffold_104_arrow_ctg1:224549..228939 (*mpdu1*) |
| Aves | *Hemignathus wilsoni* | GCA_009690805.1 | Not detected |
| Aves | *Malurus cyaneus* | GCA_009741485.1 | Not detected |
| Aves | *Geothlypis trichas* | GCA_009764595.1 | Not detected |
| Aves | *Bucorvus abyssinicus* | GCA_009769605.1 | Not detected |
| Aves | *Eremophila alpestris* | GCA_009792885.1 | Not detected |
| Aves | *Tetrax tetrax* | GCA_009806455.1 | Not detected |
| Aves | *Sialia sialis* | GCA_009812075.1 | Not detected |
| Aves | *Catharus ustulatus* | GCF_009819885.1 | chr37:1098931-1109096 (*mpdu1*) |
| Aves | *Chiroxiphia lanceolata* | GCF_009829145.1 | Not detected |
| Aves | *Apteryx mantelli* | GCF_001039765.1 | Not detected |
| Aves | *Aratinga weddellii* | GCA_009867205.1 | Not detected |
| Aves | *Psittacus timneh* | GCA_009867315.1 | Not detected |
| Aves | *Anodorhynchus hyacinthinus* | GCA_009936445.1 | Not detected |
| Aves | *Pyrrhura frontalis* | GCA_010014865.1 | Not detected |
| Aves | *Psitteuteles goldiei* | GCA_010014875.1 | Not detected |
| Aves | *Lorius garrulus* | GCA_010014925.1 | Not detected |
| Aves | *Pyrrhura griseipectus* | GCA_010014965.1 | Not detected |
| Aves | *Cacatua leadbeateri* | GCA_010015045.1 | Not detected |
| Aves | *Ara chloropterus* | GCA_010014725.1 | Not detected |
| Aves | *Eudyptes filholi* | GCA_010085365.1 | Not detected |
| Aves | *Megadyptes antipodes* | GCA_010078485.1 | Not detected |
| Aves | *Eudyptula novaehollandiae* | GCA_010078495.1 | Not detected |
| Aves | *Eudyptes schlegeli* | GCA_010080425.1 | Not detected |
| Aves | *Eudyptula albosignata* | GCA_010080465.1 | Not detected |
| Aves | *Eudyptes moseleyi* | GCA_010082375.1 | Not detected |
| Aves | *Eudyptes chrysolophus* | GCA_010084205.1 | Not detected |
| Aves | *Eudyptes robustus* | GCA_010085315.1 | Not detected |
| Aves | *Eudyptes pachyrhynchus* | GCA_010085335.1 | Not detected |
| Aves | *Eudyptes sclateri* | GCA_010078445.1 | Not detected |
| Aves | *Buteo japonicus* | GCA_010312235.1 | Not detected |
| Aves | *Pterocles orientalis* | GCA_011057875.1 | Not detected |
| Aves | *Melospiza melodia* | GCA_013398205.1 | Not detected |
| Aves | *Antigone vipio* | GCA_012488435.1 | Not detected |
| Aves | *Oxyura jamaicensis* | GCF_011077185.1 | Not detected |
| Aves | *Heteronetta atricapilla* | GCA_011075105.1 | Not detected |
| Aves | *Nettapus auritus* | GCA_011076525.1 | Not detected |
| Aves | *Stictonetta naevosa* | GCA_011074415.1 | Not detected |
| Aves | *Eupsittula pertinax* | GCA_011317185.1 | Not detected |
| Aves | *Melanerpes aurifrons* | GCA_011125475.1 | Not detected |
| Aves | *Alaudala cheleensis* | GCA_013398995.1 | Not detected |
| Aves | *Pionus senilis* | GCA_011762725.1 | Not detected |
| Aves | *Pyrrhura molinae* | GCA_011763355.1 | Not detected |
| Aves | *Pyrrhura perlata* | GCA_011763455.1 | Not detected |
| Aves | *Phalacrocorax pelagicus* | GCA_002173435.1 | Not detected |
| Aves | *Ardeotis arabs* | GCA_011801015.1 | Not detected |
| Aves | *Molothrus ater* | GCF_012460135.1 | Not detected |
| Aves | *Nyctibius grandis* | GCA_013368605.1 | Not detected |
| Aves | *Erithacus rubecula* | GCA_903797595.1 | Not detected |
| Aves | *Crypturellus undulatus* | GCA_013389825.1 | Not detected |
| Aves | *Crypturellus soui* | GCA_013389845.1 | Not detected |
| Aves | *Piaya cayana* | GCA_013389865.1 | Not detected |
| Aves | *Sula dactylatra* | GCA_013389905.1 | Not detected |
| Aves | *Aegithalos caudatus* | GCA_013389925.1 | Not detected |
| Aves | *Fregata magnificens* | GCA_013389945.1 | Not detected |
| Aves | *Todus mexicanus* | GCA_013389965.1 | Not detected |
| Aves | *Dryoscopus gambensis* | GCA_013389985.1 | Not detected |
| Aves | *Calyptomena viridis* | GCA_013390005.1 | Not detected |
| Aves | *Pitta sordida* | GCA_013390025.1 | Not detected |
| Aves | *Chloropsis hardwickii* | GCA_013390065.1 | Not detected |
| Aves | *Picathartes gymnocephalus* | GCA_013390045.1 | Not detected |
| Aves | *Tichodroma muraria* | GCA_013390105.1 | Not detected |
| Aves | *Cochlearius cochlearius* | GCA_013396365.1 | Not detected |
| Aves | *Chunga burmeisteri* | GCA_013396505.1 | Not detected |
| Aves | *Lophotis ruficrista* | GCA_013396455.1 | Not detected |
| Aves | *Tachuris rubrigastra* | GCA_013396555.1 | Not detected |
| Aves | *Crotophaga sulcirostris* | GCA_013397455.1 | Not detected |
| Aves | *Mionectes macconnelli* | GCA_013397495.1 | Not detected |
| Aves | *Halcyon senegalensis* | GCA_013397595.1 | Not detected |
| Aves | *Centropus unirufus* | GCA_013397695.1 | Not detected |
| Aves | *Onychorhynchus coronatus* | GCA_013397825.1 | Not detected |
| Aves | *Nothoprocta ornata* | GCA_013398335.1 | Not detected |
| Aves | *Aphelocoma coerulescens* | GCA_013398375.1 | Not detected |
| Aves | *Rhegmatorhina hoffmannsi* | GCA_013398505.1 | Not detected |
| Aves | *Brachypodius atriceps* | GCA_013398615.1 | Not detected |
| Aves | *Copsychus sechellarum* | GCA_013398635.1 | Not detected |
| Aves | *Nothocercus julius* | GCA_013398735.1 | Not detected |
| Aves | *Spizaetus tyrannus* | GCA_013399215.1 | Not detected |
| Aves | *Piprites chloris* | GCA_013399295.1 | Not detected |
| Aves | *Rostratula benghalensis* | GCA_013399305.1 | Not detected |
| Aves | *Herpetotheres cachinnans* | GCA_013399355.1 | Not detected |
| Aves | *Sylvietta virens* | GCA_013399515.1 | Not detected |
| Aves | *Circaetus pectoralis* | GCA_013399555.1 | Not detected |
| Aves | *Glaucidium brasilianum* | GCA_013399595.1 | Not detected |
| Aves | *Cephalopterus ornatus* | GCA_013396775.1 | Not detected |
| Aves | *Vireo altiloquus* | GCA_013396875.1 | Not detected |
| Aves | *Regulus satrapa* | GCA_013396955.1 | Not detected |
| Aves | *Pachyramphus minor* | GCA_013397135.1 | Not detected |
| Aves | *Dyaphorophyia castanea* | GCA_013397175.1 | Not detected |
| Aves | *Hylia prasina* | GCA_013397195.1 | Not detected |
| Aves | *Lanius ludovicianus* | GCA_013397235.1 | Not detected |
| Aves | *Burhinus bistriatus* | GCA_013397275.1 | Not detected |
| Aves | *Polioptila caerulea* | GCA_013397295.1 | Not detected |
| Aves | *Toxostoma redivivum* | GCA_013397375.1 | Not detected |
| Aves | *Erythrocercus mccallii* | GCA_013397435.1 | Not detected |
| Aves | *Furnarius figulus* | GCA_013397465.1 | Not detected |
| Aves | *Neopipo cinnamomea* | GCA_013397415.1 | Not detected |
| Aves | *Pterocles burchelli* | GCA_013397535.1 | Not detected |
| Aves | *Urocynchramus pylzowi* | GCA_013397555.1 | Not detected |
| Aves | *Ptilonorhynchus violaceus* | GCA_013397565.1 | Not detected |
| Aves | *Columbina picui* | GCA_013397635.1 | Not detected |
| Aves | *Chroicocephalus maculipennis* | GCA_013397655.1 | Not detected |
| Aves | *Probosciger aterrimus* | GCA_013397665.1 | Not detected |
| Aves | *Origma solitaria* | GCA_013397895.1 | Not detected |
| Aves | *Daphoenositta chrysoptera* | GCA_013397915.1 | Not detected |
| Aves | *Dasyornis broadbenti* | GCA_013397935.1 | Not detected |
| Aves | *Grantiella picta* | GCA_013397955.1 | Not detected |
| Aves | *Machaerirhynchus nigripectus* | GCA_013397975.1 | Not detected |
| Aves | *Malurus elegans* | GCA_013397995.1 | Not detected |
| Aves | *Oreocharis arfaki* | GCA_013398015.1 | Not detected |
| Aves | *Panurus biarmicus* | GCA_013398035.1 | Not detected |
| Aves | *Ifrita kowaldi* | GCA_013398055.1 | Not detected |
| Aves | *Falcunculus frontatus* | GCA_013398115.1 | Not detected |
| Aves | *Pedionomus torquatus* | GCA_013398155.1 | Not detected |
| Aves | *Nesospiza acunhae* | GCA_013398715.1 | Not detected |
| Aves | *Sapayoa aenigma* | GCA_013398745.1 | Not detected |
| Aves | *Ibidorhyncha struthersii* | GCA_013398815.1 | Not detected |
| Aves | *Mohoua ochrocephala* | GCA_013398855.1 | Not detected |
| Aves | *Prunella himalayana* | GCA_013398875.1 | Not detected |
| Aves | *Dromas ardeola* | GCA_013398915.1 | Not detected |
| Aves | *Ceuthmochares aereus* | GCA_013398935.1 | Not detected |
| Aves | *Callaeas wilsoni* | GCA_013398955.1 | Not detected |
| Aves | *Catharus fuscescens* | GCA_013398975.1 | Not detected |
| Aves | *Galbula dea* | GCA_013399015.1 | Not detected |
| Aves | *Quiscalus mexicanus* | GCA_013399035.1 | Not detected |
| Aves | *Ramphastos sulfuratus* | GCA_013399055.1 | Not detected |
| Aves | *Chloroceryle aenea* | GCA_013399075.1 | Not detected |
| Aves | *Psophia crepitans* | GCA_013399095.1 | Not detected |
| Aves | *Odontophorus gujanensis* | GCA_013399175.1 | Not detected |
| Aves | *Dicrurus megarhynchus* | GCA_013399195.1 | Not detected |
| Aves | *Rhipidura dahli* | GCA_013399225.1 | Not detected |
| Aves | *Ciconia maguari* | GCA_013399255.1 | Not detected |
| Aves | *Trogon melanurus* | GCA_013399275.1 | Not detected |
| Aves | *Fregetta grallaria* | GCA_013399335.1 | Not detected |
| Aves | *Alopecoenas beccarii* | GCA_013399365.1 | Not detected |
| Aves | *Nyctiprogne leucopyga* | GCA_013399395.1 | Not detected |
| Aves | *Anthoscopus minutus* | GCA_013399455.1 | Not detected |
| Aves | *Chauna torquata* | GCA_013399475.1 | Not detected |
| Aves | *Corythaixoides concolor* | GCA_013399495.1 | Not detected |
| Aves | *Phaetusa simplex* | GCA_013399535.1 | Not detected |
| Aves | *Podilymbus podiceps* | GCA_013399565.1 | Not detected |
| Aves | *Amazona guildingii* | GCA_013399615.1 | Not detected |
| Aves | *Chordeiles acutipennis* | GCA_013399635.1 | Not detected |
| Aves | *Setophaga kirtlandii* | GCA_013399655.1 | Not detected |
| Aves | *Formicarius rufipectus* | GCA_013399695.1 | Not detected |
| Aves | *Mesembrinibis cayennensis* | GCA_013399675.1 | Not detected |
| Aves | *Tyrannus savana* | GCA_013399735.1 | Not detected |
| Aves | *Podargus strigoides* | GCA_013399755.1 | Not detected |
| Aves | *Zosterops hypoxanthus* | GCA_013399795.1 | scaffold34452:7-144 (*mpdu1*) |
| Aves | *Leptocoma aspasia* | GCA_013399835.1 | Not detected |
| Aves | *Semnornis frantzii* | GCA_013399775.1 | scaffold105828:19-126 (*mpdu1*) |
| Aves | *Gymnorhina tibicen* | GCA_013399875.1 | Not detected |
| Aves | *Oxyruncus cristatus* | GCA_013399855.1 | Not detected |
| Aves | *Serilophus lunatus* | GCA_013399895.1 | Not detected |
| Aves | *Illadopsis cleaveri* | GCA_013399915.1 | Not detected |
| Aves | *Cinclus mexicanus* | GCA_013399935.1 | scaffold20779:344-457 (*mpdu1*) |
| Aves | *Ploceus nigricollis* | GCA_013399945.1 | scaffold C12703417:111-185 (*mpdu1*) |
| Aves | *Oenanthe oenanthe* | GCA_013399995.1 | scaffold14799:3774-3994 (*mpdu1*) |
| Aves | *Sylvia borin* | GCA_014839755.1 | Not detected |
| Aves | *Rynchops niger* | GCA_013400035.1 | Not detected |
| Aves | *Nycticryphes semicollaris* | GCA_013400055.1 | Not detected |
| Aves | *Hippolais icterina* | GCA_013400075.1 | scaffold17498:334-447 (*mpdu1*) |
| Aves | *Rhinopomastus cyanomelas* | GCA_013400115.1 | Not detected |
| Aves | *Locustella ochotensis* | GCA_013400155.1 | Not detected |
| Aves | *Rhinoptilus africanus* | GCA_013400135.1 | Not detected |
| Aves | *Phainopepla nitens* | GCA_013400175.1 | scaffold17930:229-363(*mpdu1*) |
| Aves | *Aramus guarauna* | GCA_013400195.1 | Not detected |
| Aves | *Cisticola juncidis* | GCA_013400215.1 | Not detected |
| Aves | *Oriolus oriolus* | GCA_013400235.1 | Not detected |
| Aves | *Sitta europaea* | GCA_013400255.1 | Not detected |
| Aves | *Rhadina sibilatrix* | GCA_013400275.1 | Not detected |
| Aves | *Bombycilla garrulus* | GCA_013400315.1 | scaffold15731:293-424 (*mpdu1*) |
| Aves | *Larus smithsonianus* | GCA_013400295.1 | Not detected |
| Aves | *Certhia brachydactyla* | GCA_013400355.1 | Not detected |
| Aves | *Eurystomus gularis* | GCA_013400335.1 | Not detected |
| Aves | *Thinocorus orbignyianus* | GCA_013400375.1 | Not detected |
| Aves | *Vidua macroura* | GCA_013400395.1 | scaffold13499:2223-2342(*mpdu1*) |
| Aves | *Scytalopus superciliaris* | GCA_013400415.1 | Not detected |
| Aves | *Pycnonotus jocosus* | GCA_013400435.1 | Not detected |
| Aves | *Nicator chloris* | GCA_013400495.1 | scaffold12986:384-503 (*mpdu1*) |
| Aves | *Tricholaema leucomelas* | GCA_013400475.1 | scaffold14290:4-87 (*mpdu1*) |
| Aves | *Erpornis zantholeuca* | GCA_013400515.1 | scaffold6249:1208-1321 (*mpdu1*) |
| Aves | *Chloropsis cyanopogon* | GCA_013400585.1 | scaffold2045:2450-2524 (*mpdu1*) |
| Aves | *Scopus umbretta* | GCA_013400535.1 | Not detected |
| Aves | *Peucedramus taeniatus* | GCA_013400575.1 | scaffold25097:125-410 (*mpdu1*) |
| Aves | *Hypocryptadius cinnamomeus* | GCA_013400635.1 | scaffold7659:329-460 (*mpdu1*) |
| Aves | *Syrrhaptes paradoxus* | GCA_013400615.1 | Not detected |
| Aves | *Mystacornis crossleyi* | GCA_013400655.1 | Not detected |
| Aves | *Neodrepanis coruscans* | GCA_013400695.1 | Not detected |
| Aves | *Oxylabes madagascariensis* | GCA_013400675.1 | Not detected |
| Aves | *Pomatostomus ruficeps* | GCA_013400735.1 | Not detected |
| Aves | *Pelecanoides urinatrix* | GCA_013400755.1 | Not detected |
| Aves | *Chaetops frenatus* | GCA_013400775.1 | scaffold19576:368-481 (*mpdu1*) |
| Aves | *Centropus bengalensis* | GCA_013400815.1 | Not detected |
| Aves | *Pluvianellus socialis* | GCA_013400795.1 | Not detected |
| Aves | *Zapornia atra* | GCA_013400835.1 | Not detected |
| Aves | *Buphagus erythrorhynchus* | GCA_013400855.1 | Not detected |
| Aves | *Thalassarche chlororhynchos* | GCA_013400895.1 | Not detected |
| Aves | *Pardalotus punctatus* | GCA_013400915.1 | Not detected |
| Aves | *Turnix velox* | GCA_013400935.1 | Not detected |
| Aves | *Drymodes brunneopygia* | GCA_013400955.1 | scaffold21049:157-270 (*mpdu1*) |
| Aves | *Atrichornis clamosus* | GCA_013400975.1 | scaffold C26399584:118-240 (*mpdu1*) |
| Aves | *Rissa tridactyla* | GCA_013401015.1 | Not detected |
| Aves | *Uria aalge* | GCA_013401055.1 | Not detected |
| Aves | *Horornis vulcanius* | GCA_013401035.1 | Not detected |
| Aves | *Cepphus grylle* | GCA_013401065.1 | Not detected |
| Aves | *Passerina amoena* | GCA_013401095.1 | Not detected |
| Aves | *Calonectris borealis* | GCA_013401115.1 | scaffold1780:1014-1109 (*mpdu1*) |
| Aves | *Ceyx cyanopectus* | GCA_013401355.1 | scaffold scaffold97612:79-189 (*mpdu1*) |
| Aves | *Cettia cetti* | GCA_013401135.1 | scaffold NODE_32634_length_1756_cov_3.44669_ID_65267:838-951 (*mpdu1*) |
| Aves | *Xiphorhynchus elegans* | GCA_013401175.1 | Not detected |
| Aves | *Pteruthius melanotis* | GCA_013401235.1 | Not detected |
| Aves | *Urocolius indicus* | GCA_013401255.1 | Not detected |
| Aves | *Brachypteracias leptosomus* | GCA_013401335.1 | Not detected |
| Aves | *Glareola pratincola* | GCA_013401295.1 | Not detected |
| Aves | *Balaeniceps rex* | GCA_013401315.1 | Not detected |
| Aves | *Alcedo cyanopectus* | GCA_013401355.1 | Not detected |
| Aves | *Spizella passerina* | GCA_013401375.1 | scaffold81442:9-122 (*mpdu1*) |
| Aves | *Ardeotis kori* | GCA_013396375.1 | Not detected |
| Aves | *Anhinga anhinga* | GCA_013396435.1 | Not detected |
| Aves | *Smithornis capensis* | GCA_013396465.1 | Not detected |
| Aves | *Chaetorhynchus papuensis* | GCA_013396525.1 | Not detected |
| Aves | *Ptilorrhoa leucosticta* | GCA_013396485.1 | Not detected |
| Aves | *Rhodinocichla rosea* | GCA_013396575.1 | Not detected |
| Aves | *Nyctibius bracteatus* | GCA_013396595.1 | Not detected |
| Aves | *Oceanites oceanicus* | GCA_013396615.1 | Not detected |
| Aves | *Penelope pileata* | GCA_013396635.1 | Not detected |
| Aves | *Campylorhamphus procurvoides* | GCA_013396655.1 | Not detected |
| Aves | *Eubucco bourcierii* | GCA_013396675.1 | Not detected |
| Aves | *Sakesphorus luctuosus* | GCA_013396695.1 | Not detected |
| Aves | *Grallaria varia* | GCA_013396735.1 | Not detected |
| Aves | *Sclerurus mexicanus* | GCA_013396755.1 | Not detected |
| Aves | *Ciccaba nigrolineata* | GCA_013396715.1 | Not detected |
| Aves | *Corythaeola cristata* | GCA_013396815.1 | Not detected |
| Aves | *Psilopogon haemacephalus* | GCA_013396835.1 | Not detected |
| Aves | *Hemiprocne comata* | GCA_013396855.1 | Not detected |
| Aves | *Loxia leucoptera* | GCA_013396895.1 | Not detected |
| Aves | *Stercorarius parasiticus* | GCA_013396915.1 | Not detected |
| Aves | *Bucco capensis* | GCA_013396975.1 | Not detected |
| Aves | *Baryphthengus martii* | GCA_013396935.1 | Not detected |
| Aves | *Dicaeum eximium* | GCA_013396995.1 | Not detected |
| Aves | *Myiagra hebetior* | GCA_013397015.1 | Not detected |
| Aves | *Hydrobates tethys* | GCA_013397025.1 | Not detected |
| Aves | *Pachycephala philippinensis* | GCA_013397055.1 | Not detected |
| Aves | *Rhabdornis inornatus* | GCA_013397075.1 | Not detected |
| Aves | *Sterrhoptilus dennistouni* | GCA_013397095.1 | Not detected |
| Aves | *Edolisoma coerulescens* | GCA_013397115.1 | Not detected |
| Aves | *Irena cyanogastra* | GCA_013397155.1 | Not detected |
| Aves | *Thryothorus ludovicianus* | GCA_013397245.1 | Not detected |
| Aves | *Leucopsar rothschildi* | GCA_013397325.1 | Not detected |
| Aves | *Donacobius atricapilla* | GCA_013397315.1 | Not detected |
| Aves | *Calcarius ornatus* | GCA_013397715.1 | Not detected |
| Aves | *Cnemophilus loriae* | GCA_013397755.1 | Not detected |
| Aves | *Eulacestoma nigropectus* | GCA_013397775.1 | Not detected |
| Aves | *Orthonyx spaldingii* | GCA_013397795.1 | Not detected |
| Aves | *Aleadryas rufinucha* | GCA_013397815.1 | Not detected |
| Aves | *Melanocharis versteri* | GCA_013397845.1 | Not detected |
| Aves | *Struthidea cinerea* | GCA_013397865.1 | Not detected |
| Aves | *Aegotheles bennettii* | GCA_013398125.1 | Not detected |
| Aves | *Climacteris rufus* | GCA_013398175.1 | Not detected |
| Aves | *Steatornis caripensis* | GCA_013398225.1 | Not detected |
| Aves | *Promerops cafer* | GCA_013398275.1 | Not detected |
| Aves | *Chionis minor* | GCA_013398295.1 | Not detected |
| Aves | *Nothoprocta pentlandii* | GCA_013398315.1 | Not detected |
| Aves | *Nothocercus nigrocapillus* | GCA_013398345.1 | Not detected |
| Aves | *Pheucticus melanocephalus* | GCA_013398405.1 | Not detected |
| Aves | *Asarcornis scutulata* | GCA_013398475.1 | Not detected |
| Aves | *Certhia familiaris* | GCA_013398575.1 | Not detected |
| Aves | *Cercotrichas coryphoeus* | GCA_013398595.1 | Not detected |
| Aves | *Acrocephalus arundinaceus* | GCA_013398685.1 | Not detected |
| Aves | *Spelaeornis formosus* | GCA_013398795.1 | Not detected |
| Aves | *Corvus monedula* | GCA_013407035.1 | Not detected |
| Aves | *Columba janthina* | GCA_014362705.1 | Not detected |
| Aves | *Fratercula cirrhata* | GCA_014363165.1 | Not detected |
| Aves | *Haliaeetus pelagicus* | GCA_014363185.1 | scaffold Hape_6205:8946-9056 (*mpdu1*) |
| Aves | *Lycocorax pyrrhopterus* | GCA_014706015.1 | Not detected |
| Aves | *Pyrrhura lepida* | GCA_015163975.1 | Not detected |
| Aves | *Pogoniulus pusillus* | GCA_015220805.1 | scaffold_102_arrow_ctg1:34551-34646 (*mpdu1*) |
| Aves | *Lamprotornis superbus* | GCA_015883425.1 | Not detected |
| Elasmobranchii | *Amblyraja radiata* | GCF_010909765.1 | Not detected |
| Elasmobranchii | *Carcharodon carcharias* | GCA_902204185.2 | Not detected |
| Elasmobranchii | *Chiloscyllium plagiosum* | GCA_004010195.1 | chr48:899095-901719 (*sox15*) |
| Elasmobranchii | *Chiloscyllium punctatum* | GCA_003427335.1 | scf_chipu00008499:3-830 (*sox15*) |
| Elasmobranchii | *Leucoraja erinacea* | GCA_000238235.1 | Not detected |
| Elasmobranchii | *Pristis pectinata* | GCA_009764485.1 | chr37:13017081-13019705 (*sox15*) |
| Elasmobranchii | *Rhincodon typus* | GCF_001642345.1 | Not detected |
| Elasmobranchii | *Scyliorhinus torazame* | GCA_003427355.1 | Not detected |
| Holocephali | *Callorhinchus milii* | GCA_000165045.2 | Not detected |
| Holocephali | *Hydrolagus affinis* | GCA_012026655.1 | Not detected |
| Marsupials | *Gymnobelideus leadbeateri* | GCA_011680675.1 | contig_4989:60634-60813 (*sox15*) |
| Marsupials | *Monodelphis domestica* | GCF_000002295.2 | chr2:255024566-255027848 (*sox15*) |
| Marsupials | *Notamacropus eugenii* | GCA_000004035.1 | Scaffold_49011:49333-49593 (*sox15*) |
| Marsupials | *Phascolarctos cinereus* | GCF_002099425.1 | scaf00168:5655310-5657745 (*sox15*) |
| Marsupials | *Sarcophilus harrisii* | GCF_902635505.1 | chr4:289670298..289672062 (*sox15*) |
| Marsupials | *Thylacinus cynocephalus* | GCA_007646695.1 | Tcyn_scaff_497:1891437-1891661 (*sox15*) |
| Marsupials | *Trichosurus vulpecula* | GCA_011100635.1 | chr7:260860768-260860992 (*sox15*) |
| Marsupials | *Vombatus ursinus* | GCF_900497805.2 | scaffold UNPS02011916.1:1268801-1272512 (*sox15*) |
| Squamata | *Anolis carolinensis* | GCF_000090745.1 | scaffold chrUn4585:2155-6179 (*mpdu1*)  scaffold chrUn1025:2888-46342 (*fxr2*) |
| Squamata | *Crotalus horridus* | GCA_001625485.1 | scaffold Sequence_2573_46555:46517-46293 (*sox15*) |
| Squamata | *Crotalus pyrrhus* | GCA_000737285.1 | CMI_contig_390494:2217-2002 (*sox15*) |
| Squamata | *Crotalus viridis viridis* | GCA_003400415.2 | chr12:15321120-15327424 (*fxr2*) |
| Squamata | *Emydocephalus ijimae* | GCA_004319985.1 | scaffold C22860292:281-412 (*mpdu1*)  scaffold186636:204-6088 (*fxr2*) |
| Squamata | *Gekko japonicus* | GCF_001447785.1 | scaffold336:965065-1065024 (*fxr2* and *mpdu1*) |
| Squamata | *Hydrophis cyanocinctus* | GCA_004023725.1 | scaffold433609:4333-5902 (*mpdu1*)  scaffold509787:2920-14224 (*fxr2*) |
| Squamata | *Hydrophis hardwickii* | GCA_004023765.1 | scaffold209275:206-337 (*mpdu1*)  scaffold143923:8977-29049 (*fxr2*) |
| Squamata | *Hydrophis melanocephalus* | GCA_004320005.1 | scaffold3384131:251-382 (*mpdu1*)  scaffold481365:21757-41686 (*fxr2*) |
| Squamata | *Lacerta agilis* | GCF_009819535.1 | chr14:2425436-2431814 (*sox15*) |
| Squamata | *Lacerta bilineata* | GCA_900245895.1 | contig: Lbil_405:156086-155862 (*sox15*) |
| Squamata | *Lacerta viridis* | GCA_900245905.1 | contig: Lvir_428:88450-88226 (*sox15*) |
| Squamata | *Laticauda colubrina* | GCA_004320045.1 | scaffold1584:1-76475 (*fxr2* and *mpdu1*) |
| Squamata | *Laticauda laticaudata* | GCA_004320025.1 | Contig23525:568-1326 (*mpdu1*)  Contig23524:2828-8508 (*fxr2*) |
| Squamata | *Naja naja* | GCA_009733165.1 | chrZ:152755000-152840000 (*fxr2* and *mpdu1*) |
| Squamata | *Notechis scutatus* | GCF_900518725.1 | scaffold TS10Xv2-PRI ULFQ01001107.1:1-38665 (*fxr2* and *mpdu1*) |
| Squamata | *Ophiophagus hannah* | GCA_000516915.1 | scaffold166773.1:88-219 (*mpdu1*)  scaffold50123.1:173-766 (*fxr2*) |
| Squamata | *Pantherophis guttatus* | GCF_001185365.1 | scaffold UNIGE_PanGut_3.0 411:80681-83270 (*sox15*) |
| Squamata | *Pantherophis obsoletus* | GCA_012654085.1 | scaffold EOBS11 588823:11747-11971 (*sox15*) |
| Squamata | *Paroedura picta* | GCA_003118565.1 | scaffold EOBS11 588823: (*fxr2* and *mpdu1*) |
| Squamata | *Podarcis muralis* | GCF_004329235.1 | chr13:53785071-53791029 (*sox15*) |
| Squamata | *Pogona vitticeps* | GCF_900067755.1 | contig1956:907893-907783 (*sox15*) |
| Squamata | *Protobothrops flavoviridis* | GCA_003402635.1 | habu1_scaffold399928:708-5082 (*mpdu1*)  habu1_scaffold4258:326920-351898 (*fxr2*) |
| Squamata | *Protobothrops mucrosquamatus* | GCF_001527695.2 | scaffold3821:3941-4165 (*sox15*) |
| Squamata | *Pseudonaja textilis* | GCF_900518735.1 | scaffold EBS10Xv2-PRI ULFR01000301.1:1-221020 (*fxr2* and *mpdu1*) |
| Squamata | *Ptyas mucosa* | GCA_012654045.1 | scaffold Pmuc001 222:65233-65009 (*sox15*) |
| Squamata | *Python bivittatus* | GCF_000186305.1 | Scaffold8063:1-36286 (*fxr2* and *mpdu1*) |
| Squamata | *Salvator merianae* | GCA_003586115.2 | Scaffold_51:3120000-3205000 (*sox15*) |
| Squamata | *Thamnophis elegans* | GCF_009769535.1 | chr3:6120087-6120194 (*mpdu1*)  chr10:61067848-61102163 (*fxr2*) |
| Squamata | *Thamnophis sirtalis* | GCF_001077635.1 | Scaffold2529:77487-77711 (*sox15*) |
| Squamata | *Thermophis baileyi* | GCA_003457575.1 | scaffold753:183788-183564 (*sox15*) |
| Squamata | *Varanus komodoensis* | GCA_004798865.1 | scaffod87:1520000-1590000 (*fxr2* and *mpdu1*) |
| Squamata | *Vipera berus berus* | GCA_000800605.1 | scaffold_1333:121594-121818 (*sox15*) |
| Squamata | *Zootoca vivipara* | GCF_011800845.1 | LG13:46204000-46209975 (*sox15*) |
| Testudines | *Actinemys marmorata* | GCA_009430475.1 | Contig48071.1:124-348 (*sox15*) |
| Testudines | *Apalone spinifera* | GCA_000385615.1 | scaffold2152:498611-498760 (*sox15*) |
| Testudines | *Carettochelys insculpta* | GCA_007922185.1 | Contig15941.1:838-1062 (*sox15*) |
| Testudines | *Chelonia mydas* | GCF_000344595.1 | Not detected |
| Testudines | *Chelonoidis abingdonii* | GCF_003597395.1 | Contig217:24437-99038 (*fxr2* and *mpdu1*) |
| Testudines | *Chelydra serpentina* | GCA_007922165.1 | Scaffold105:16701641-16701727 (*mpdu1*)  Contig63869.1:2-2804 (*fxr2*) |
| Testudines | *Chrysemys picta* | GCA_011386835.1 | Scaffold707:78330-82324 (*sox15*) |
| Testudines | *Cuora amboinensis* | GCA_004028625.2 | Scaffold1301:361985-362071 (*mpdu1*)  Scaffold21288:11-1029 (*fxr2*) |
| Testudines | *Cuora mccordi* | GCA_003846335.1 | Contig18005.1:365-141 (*sox15*) |
| Testudines | *Dermatemys mawii* | GCA_007922305.1 | Contig3352.1:75-200 (*mpdu1*)  Scaffold23244:247-8127 (*fxr2*) |
| Testudines | *Dermochelys coriacea* | GCA_009764565.2 | chr28:1073176-1072952 (*sox15*) |
| Testudines | *Emydura subglobosa* | GCA_007922225.1 | Scaffold101:14494360-14494584 (*sox15*) |
| Testudines | *Gopherus agassizii* | GCA_002896415.1 | sacffold_19907:2333-2109 (*sox15*) |
| Testudines | *Gopherus evgoodei* | GCF_007399415.2 | scaffold_88_arrow_ctg1:168347-171689 (*sox15*) |
| Testudines | *Malaclemys terrapin terrapin* | GCA_001728815.2 | scaffold utg7180000011491_pilon_pilon:24716-24492 (*sox15*) |
| Testudines | *Mesoclemmys tuberculata* | GCA_007922155.1 | Scaffold89:49025559-49025335 (*sox15*) |
| Testudines | *Pelodiscus sinensis* | GCF_000230535.1 | scaffold348:95551-99067 (*sox15*) |
| Testudines | *Pelusios castaneus* | GCA_007922175.1 | Scaffold366:478506-478282 (*sox15*) |
| Testudines | *Platysternon megacephalum* | GCA_003942145.1 | scaf496:81396-81620 (*sox15*) |
| Testudines | *Podocnemis expansa* | GCA_007922195.1 | Scaffold232:223254-223478 (*sox15*) |
| Testudines | *Terrapene carolina triunguis* | GCA_002925995.2 | Scaffold433:503415-504587 (*sox15*) |
| Testudines | *Trachemys scripta elegans* | GCF_013100865.1 | chr24:29703-31950 (*sox15*) |
| Crocodilia | *Alligator mississippiensis* | GCA_000281125.4 | unplaced genomic scaffold NW_017708085.1:95728-98901 (*mpdu1*)  unplaced genomic scaffold NW_017710318.1:7-9152 (*fxr2*) |
| Crocodilia | *Alligator sinensis* | GCA_000455745.1 | scaffold1488_1: 297783- 319347 (*mpdu1* and *fxr2*) |
| Crocodilia | *Gavialis gangeticus* | GCA_001723915.1 | Not detected |
| Crocodilia | *Crocodylus porosus* | GCF_001723895.1 | scaffold SciaK46_447:107724-817990 (*mpdu1* and *fxr2*) |
| Eutheria | *Homo sapiens* | GCA_000001405.28 | chr17:7588178-7590094 (*SOX15*) |
| Eutheria | *Macaca fascicularis* | GCA_000364345.1 | chr16:7658083-7660924 (*Sox15*) |
| Eutheria | *Mus musculus* | GCA_000001635.9 | chr11:69545863-69547553 (*Sox15*) |
| Eutheria | *Oryctolagus cuniculus* | GCA_000003625.1 | chr19:11590792-11593157 (*Sox15*) |
| Eutheria | *Felis catus* | GCA_000181335.4 | chrE1:2488692-2491111 (*Sox15*) |
| Eutheria | *Canis lupus familiaris* | GCA_014441545.1 | chr5:32613127-32614971 (*Sox15*) |
| Eutheria | *Orcinus orca* | GCA_000331955.2 | Scaffold20:11303744-11311163 (*Sox15*) |
| Eutheria | *Bos taurus* | GCA_002263795.2 | chr19:27319235-27321042 (*Sox15*) |
| Eutheria | *Loxodonta africana* | GCA_000001905.1 | scaffold_47:11626097-11628386 (*Sox15*) |
| Monotremata | *Ornithorhynchus anatinus* | GCA_004115215.4 | chrX5:6856395-6860405 (*Sox15*) |
| Monotremata | *Tachyglossus aculeatus* | GCA_015852505.1 | chrY4:3157213-3163885 (*Sox15*) |
| Elasmobranchii | *Pristis pectinata* | GCA_009764475.2 | chr37:13017081-13019705 (*sox15*) |
| Elasmobranchii | *Chiloscyllium plagiosum* | GCA_004010195.1 | chr48:899095-901719 (*sox15*) |
| Holocephali | *Callorhinchus milii* | GCA_000165045.2 | Not detected |
| Actinopterygii | *Erpetoichthys calabaricus* | GCA_900747795.3 | chr3:279086489-279121772 (*sox19*) |
| Actinopterygii | *Acipenser ruthenus* | GCA_010645085.1 | chr52:3314348-3314598 (*sox19*) chr56:798961-799211 (*sox19*) |
| Actinopterygii | *Lepisosteus oculatus* | GCA_000242695.1 | LG2:63663123-63677381 (*sox19*) |
| Actinopterygii | *Scleropages formosus* | GCA_900964775.1 | chr25:9586134-9584612 (*sox19a*) chr13:28645904-28649560 (*sox19b*) |
| Actinopterygii | *Danio rerio* | GCA_000002035.4 | chr5:24201444-24199180 (*sox19a*) chr7:26497947-26494585 (*sox19b*) |
| Actinopterygii | *Denticeps clupeoides* | GCA_900700375.2 | chr6:24275380-24273645 (*sox19a*) chr1:37097183-37094100 (*sox19b*) |
| Actinopterygii | *Hippoglossus stenolepis* | GCA_013339905.1 | chr23:15733584-15727995 (*sox19*) |
| Actinopterygii | *Maylandia zebra* | GCA_000238955.5 | LG3:34818308-34813889 (*sox19*) |
| Actinopterygii | *Oryzias latipes* | GCA_002234675.1 | chr18:26496920-26493298 (*sox19*) |
| Actinopterygii | *Perca flavescens* | GCA_004354835.1 | chr19:28330982-28336195 |
| Actinopterygii | *Larimichthys crocea* | GCA_000972845.2 | chrXI:23569225-23563502 (*sox19*) |
| Actinopterygii | *Takifugu rubripes* | GCA_901000725.3 | chr8:10572467-10575460 (*sox19*) |
| Coelacanthiformes | *Latimeria chalumnae* | GCA_000225785.1 | scaffold01231:140867-160711 (*sox15*) |
| Gymnophiona | *Geotrypetes seraphini* | GCA_902459505.2 | chr16:33228174-33245113 (*sox15*) |
| Gymnophiona | *Rhinatrema bivittatum* | GCA_901001135.2 | chr16:19893621-19904668 (*sox15*) |

**Table S2. The presence (+) and absence (-) of *sox15*, *mpdu1*, and *fxr2* on reptile genomes used in Fig. 2a, b.**

| Phylogeny | Species | *sox15* | *mpdu1* | *fxr2* | Synteny | Status in Figure 2 |
| --- | --- | --- | --- | --- | --- | --- |
| Pleurodira | *Emydura subglobosa* | + | - | + | sox15-fxr2 | sox15 + |
| Pleurodira | *Mesoclemmys tuberculata* | + | + | + | mpdu1-sox15-fxr2 | sox15 + |
| Pleurodira | *Pelusios castaneus* | + | + | + | mpdu1-sox15-fxr2 | sox15 + |
| Pleurodira | *Podocnemis expansa* | + | + | + | mpdu1-sox15-fxr2 | sox15 + |
| Trionychoidea | *Carettochelys insculpta* | + | + | + | sox15 | sox15 + |
| Trionychoidea | *Apalone spinifera* | + | + | + | mpdu1-sox15-fxr2 | sox15 + |
| Trionychoidea | *Pelodiscus sinensis* | + | + | + | mpdu1-sox15-fxr2 | sox15 + |
| Chelonioidea | *Chelonia mydas* | - | - | + | None | unknown |
| Chelonioidea | *Dermochelys coriacea* | + | + | + | mpdu1-sox15-fxr2 | sox15 + |
| Chelydridae | *Chelydra serpentina* | - | - | + | None | unknown |
| Kinosternoidea | *Dermatemys mawii* | - | + | + | None | unknown |
| Testudinoidea | *Actinemys marmorata* | + | + | + | sox15-mpdu1 | sox15 + |
| Testudinoidea | *Chrysemys picta* | + | + | + | mpdu1-sox15-fxr2 | sox15 + |
| Testudinoidea | *Malaclemys terrapin terrapin* | + | + | + | mpdu1-sox15-fxr2 | sox15 + |
| Testudinoidea | *Terrapene carolina triunguis* | + | + | + | mpdu1-sox15-fxr2 | sox15 + |
| Testudinoidea | *Trachemys scripta elegans* | + | + | + | mpdu1-sox15-fxr2 | sox15 + |
| Testudinoidea | *Cuora amboinensis* | - | + | + | None | unknown |
| Testudinoidea | *Cuora mccordi* | + | + | + | None | sox15 + |
| Testudinoidea | *Platysternon megacephalum* | + | + | + | mpdu1-sox15-fxr2 | sox15 + |
| Testudinoidea | *Chelonoidis abingdonii* | - | + | + | mpdu1-fxr2 | sox15 - |
| Testudinoidea | *Gopherus agassizii* | + | + | + | mpdu1-sox15-fxr2 | sox15 + |
| Testudinoidea | *Gopherus evgoodei* | + | + | + | mpdu1-sox15-fxr2 | sox15 + |
| Gekkonidae | *Gekko japonicus* | - | + | + | mpdu1-fxr2 | sox15 - |
| Gekkonidae | *Paroedura picta* | - | + | + | mpdu1-fxr2 | sox15 - |
| Varanidae | *Varanus komodoensis* | - | + | + | mpdu1-fxr2 | sox15 - |
| Pythonidae | *Python bivittatus* | - | + | + | mpdu1-fxr2 | sox15 - |
| Elapidae | *Laticauda colubrina* | - | + | + | mpdu1-fxr2 | sox15 - |
| Elapidae | *Naja naja* | - | + | + | mpdu1-fxr2 | sox15 - |
| Elapidae | *Notechis scutatus* | - | + | + | mpdu1-fxr2 | sox15 - |
| Elapidae | *Pseudonaja textilis* | - | + | + | mpdu1-fxr2 | sox15 - |
| Elapidae | *Laticauda laticaudata* | - | + | + | mpdu1-fxr2 | sox15 - |
| Teiidae | *Salvator merianae* | + | + | + | mpdu1-sox15-fxr2 | sox15 + |
| Lacertidae | *Lacerta agilis* | + | + | + | mpdu1-sox15-fxr2 | sox15 + |
| Lacertidae | *Lacerta bilineata* | + | + | + | mpdu1-sox15-fxr2 | sox15 + |
| Lacertidae | *Lacerta viridis* | + | + | + | mpdu1-sox15-fxr2 | sox15 + |
| Lacertidae | *Podarcis muralis* | + | + | + | mpdu1-sox15-fxr2 | sox15 + |
| Lacertidae | *Zootoca vivipara* | + | + | + | mpdu1-sox15-fxr2 | sox15 + |
| Agamidae | *Pogona vitticeps* | + | + | + | mpdu1-sox15-fxr2 | sox15 + |
| Viperidae | *Crotalus horridus* | + | + | + | None | sox15 + |
| Viperidae | *Crotalus pyrrhus* | + | + | + | None | sox15 + |
| Viperidae | *Protobothrops mucrosquamatus* | + | + | + | mpdu1-sox15 | sox15 + |
| Viperidae | *Vipera berus berus* | + | + | + | mpdu1-sox15-fxr2 | sox15 + |
| Colubridae | *Thamnophis elegans* | + | + | + | mpdu1-sox15-fxr2 | sox15 + |
| Colubridae | *Pantherophis guttatus* | + | + | + | mpdu1-sox15 | sox15 + |
| Colubridae | *Pantherophis obsoletus* | + | + | + | mpdu1-sox15 | sox15 + |
| Colubridae | *Ptyas mucosa* | + | + | + | mpdu1-sox15-fxr2 | sox15 + |
| Colubridae | *Thamnophis sirtalis* | + | + | + | mpdu1-sox15-fxr2 | sox15 + |
| Colubridae | *Thermophis baileyi* | + | + | + | mpdu1-sox15-fxr2 | sox15 + |
| Dactyloidae | *Anolis carolinensis* | - | + | - | None | unknown |
| Viperidae | *Crotalus viridis viridis* | - | - | + | None | unknown |
| Viperidae | *Protobothrops flavoviridis* | - | + | + | None | unknown |
| Elapidae | *Emydocephalus ijimae* | - | + | + | None | unknown |
| Elapidae | *Hydrophis cyanocinctus* | - | + | + | None | unknown |
| Elapidae | *Hydrophis hardwickii* | - | + | + | None | unknown |
| Elapidae | *Hydrophis melanocephalus* | - | + | + | None | unknown |
| Elapidae | *Ophiophagus hannah* | - | - | - | None | unknown |
| Alligatoridae | *Alligator mississippiensis* | - | + | + | None | unknown |
| Alligatoridae | *Alligator sinensis* | - | + | + | mpdu1-fxr2 | sox15 - |
| Crocodylidae | *Crocodylus porosus* | - | + | + | mpdu1-fxr2 | sox15 - |
| Crocodylidae | *Gavialis gangeticus* | - | - | - | None | unknown |
| Aves | *Acanthisitta chloris* | - | - | - | None | sox15 - |
| Aves | *Accipiter gentilis* | - | + | - | None | sox15 - |
| Aves | *Accipiter nisus* | - | + | - | None | sox15 - |
| Aves | *Acridotheres javanicus* | - | - | - | None | sox15 - |
| Aves | *Acrocephalus arundinaceus* | - | + | - | None | sox15 - |
| Aves | *Aegithalos caudatus* | - | - | - | None | sox15 - |
| Aves | *Aegotheles bennettii* | - | - | - | None | sox15 - |
| Aves | *Agapornis roseicollis* | - | - | - | None | sox15 - |
| Aves | *Agelaius phoeniceus* | - | - | - | None | sox15 - |
| Aves | *Alauda arvensis* | - | - | - | None | sox15 - |
| Aves | *Alaudala cheleensis* | - | + | - | None | sox15 - |
| Aves | *Alca torda* | - | - | - | None | sox15 - |
| Aves | *Alcedo cyanopectus* | - | + | - | None | sox15 - |
| Aves | *Aleadryas rufinucha* | - | + | - | None | sox15 - |
| Aves | *Alectura lathami* | - | - | - | None | sox15 - |
| Aves | *Alopecoenas beccarii* | - | - | - | None | sox15 - |
| Aves | *Amazona aestiva* | - | - | - | None | sox15 - |
| Aves | *Amazona collaria* | - | - | - | None | sox15 - |
| Aves | *Amazona guildingii* | - | - | - | None | sox15 - |
| Aves | *Amazona vittata* | - | - | - | None | sox15 - |
| Aves | *Anas platyrhynchos* | - | - | - | None | sox15 - |
| Aves | *Anas zonorhyncha* | - | - | - | None | sox15 - |
| Aves | *Anhinga anhinga* | - | - | - | None | sox15 - |
| Aves | *Anhinga rufa* | - | - | - | None | sox15 - |
| Aves | *Anodorhynchus hyacinthinus* | - | + | - | None | sox15 - |
| Aves | *Anomalopteryx didiformis* | - | - | - | None | sox15 - |
| Aves | *Anser brachyrhynchus* | - | - | - | None | sox15 - |
| Aves | *Anser cygnoides* | - | - | - | None | sox15 - |
| Aves | *Anser indicus* | - | - | - | None | sox15 - |
| Aves | *Anseranas semipalmata* | - | - | - | None | sox15 - |
| Aves | *Anthochaera phrygia* | - | - | - | None | sox15 - |
| Aves | *Anthoscopus minutus* | - | + | - | None | sox15 - |
| Aves | *Antigone vipio* | - | - | - | None | sox15 - |
| Aves | *Antrostomus carolinensis* | - | - | - | None | sox15 - |
| Aves | *Apaloderma vittatum* | - | - | - | None | sox15 - |
| Aves | *Aphelocoma coerulescens* | - | - | - | None | sox15 - |
| Aves | *Aptenodytes forsteri* | - | - | - | None | sox15 - |
| Aves | *Aptenodytes patagonicus* | - | - | - | None | sox15 - |
| Aves | *Apteryx haastii* | - | - | - | None | sox15 - |
| Aves | *Apteryx mantelli* | - | - | - | None | sox15 - |
| Aves | *Apteryx owenii* | - | + | - | None | sox15 - |
| Aves | *Apteryx rowi* | - | - | - | None | sox15 - |
| Aves | *Aquila chrysaetos* | - | - | - | None | sox15 - |
| Aves | *Ara ararauna* | - | + | - | None | sox15 - |
| Aves | *Ara chloropterus* | - | + | - | None | sox15 - |
| Aves | *Ara glaucogularis* | - | - | - | None | sox15 - |
| Aves | *Ara macao* | - | - | - | None | sox15 - |
| Aves | *Ara militaris* | - | + | - | None | sox15 - |
| Aves | *Aramus guarauna* | - | - | - | None | sox15 - |
| Aves | *Aratinga solstitialis* | - | - | - | None | sox15 - |
| Aves | *Aratinga weddellii* | - | - | - | None | sox15 - |
| Aves | *Ardeotis arabs* | - | - | - | None | sox15 - |
| Aves | *Ardeotis kori* | - | - | - | None | sox15 - |
| Aves | *Arenaria interpres* | - | - | - | None | sox15 - |
| Aves | *Asarcornis scutulata* | - | - | - | None | sox15 - |
| Aves | *Athene cunicularia* | - | - | - | None | sox15 - |
| Aves | *Atlantisia rogersi* | - | - | - | None | sox15 - |
| Aves | *Atrichornis clamosus* | - | + | - | None | sox15 - |
| Aves | *Aythya fuligula* | - | - | - | None | sox15 - |
| Aves | *Balaeniceps rex* | - | - | - | None | sox15 - |
| Aves | *Balearica regulorum* | - | - | - | None | sox15 - |
| Aves | *Bambusicola thoracicus* | - | - | - | None | sox15 - |
| Aves | *Baryphthengus martii* | - | - | - | None | sox15 - |
| Aves | *Bombycilla garrulus* | - | + | - | None | sox15 - |
| Aves | *Brachypodius atriceps* | - | - | - | None | sox15 - |
| Aves | *Brachypteracias leptosomus* | - | - | - | None | sox15 - |
| Aves | *Branta canadensis* | - | - | - | None | sox15 - |
| Aves | *Bubo blakistoni* | - | + | - | None | sox15 - |
| Aves | *Bubo bubo* | - | - | - | None | sox15 - |
| Aves | *Bucco capensis* | - | - | - | None | sox15 - |
| Aves | *Buceros rhinoceros* | - | - | - | None | sox15 - |
| Aves | *Bucorvus abyssinicus* | - | - | - | None | sox15 - |
| Aves | *Buphagus erythrorhynchus* | - | - | - | None | sox15 - |
| Aves | *Burhinus bistriatus* | - | - | - | None | sox15 - |
| Aves | *Burhinus oedicnemus* | - | - | - | None | sox15 - |
| Aves | *Buteo japonicus* | - | - | - | None | sox15 - |
| Aves | *Cacatua leadbeateri* | - | + | - | None | sox15 - |
| Aves | *Cairina moschata domestica* | - | + | - | None | sox15 - |
| Aves | *Calcarius ornatus* | - | - | - | None | sox15 - |
| Aves | *Calidris pugnax* | - | - | - | None | sox15 - |
| Aves | *Calidris pygmaea* | - | - | - | None | sox15 - |
| Aves | *Callaeas wilsoni* | - | + | - | None | sox15 - |
| Aves | *Callipepla squamata* | - | + | - | None | sox15 - |
| Aves | *Callipepla squamata* | - | - | - | None | sox15 - |
| Aves | *Caloenas nicobarica* | - | - | - | None | sox15 - |
| Aves | *Calonectris borealis* | - | + | - | None | sox15 - |
| Aves | *Calypte anna* | - | - | - | None | sox15 - |
| Aves | *Calyptomena viridis* | - | - | - | None | sox15 - |
| Aves | *Camarhynchus parvulus* | - | + | - | None | sox15 - |
| Aves | *Campylorhamphus procurvoides* | - | - | - | None | sox15 - |
| Aves | *Cardinalis cardinalis* | - | - | - | None | sox15 - |
| Aves | *Cariama cristata* | - | - | - | None | sox15 - |
| Aves | *Casuarius casuarius* | - | - | - | None | sox15 - |
| Aves | *Cathartes aura* | - | - | - | None | sox15 - |
| Aves | *Catharus fuscescens* | - | + | - | None | sox15 - |
| Aves | *Catharus ustulatus* | - | + | - | None | sox15 - |
| Aves | *Centrocercus minimus* | - | + | - | None | sox15 - |
| Aves | *Centropus bengalensis* | - | - | - | None | sox15 - |
| Aves | *Centropus unirufus* | - | - | - | None | sox15 - |
| Aves | *Cephalopterus ornatus* | - | - | - | None | sox15 - |
| Aves | *Cepphus grylle* | - | - | - | None | sox15 - |
| Aves | *Cercotrichas coryphoeus* | - | - | - | None | sox15 - |
| Aves | *Certhia brachydactyla* | - | - | - | None | sox15 - |
| Aves | *Certhia familiaris* | - | - | - | None | sox15 - |
| Aves | *Cettia cetti* | - | + | - | None | sox15 - |
| Aves | *Ceuthmochares aereus* | - | - | - | None | sox15 - |
| Aves | *Chaetops frenatus* | - | + | - | None | sox15 - |
| Aves | *Chaetorhynchus papuensis* | - | - | - | None | sox15 - |
| Aves | *Chaetura pelagica* | - | - | - | None | sox15 - |
| Aves | *Charadrius alexandrinus* | - | - | - | None | sox15 - |
| Aves | *Charadrius vociferus* | - | - | - | None | sox15 - |
| Aves | *Chauna torquata* | - | - | - | None | sox15 - |
| Aves | *Chionis minor* | - | - | - | None | sox15 - |
| Aves | *Chiroxiphia lanceolata* | - | - | - | None | sox15 - |
| Aves | *Chlamydotis macqueenii* | - | - | - | None | sox15 - |
| Aves | *Chlamydotis undulata* | - | - | - | None | sox15 - |
| Aves | *Chloroceryle aenea* | - | - | - | None | sox15 - |
| Aves | *Chloropsis cyanopogon* | - | + | - | None | sox15 - |
| Aves | *Chloropsis hardwickii* | - | - | - | None | sox15 - |
| Aves | *Chordeiles acutipennis* | - | - | - | None | sox15 - |
| Aves | *Chroicocephalus maculipennis* | - | - | - | None | sox15 - |
| Aves | *Chrysolophus pictus* | - | - | - | None | sox15 - |
| Aves | *Chunga burmeisteri* | - | - | - | None | sox15 - |
| Aves | *Ciccaba nigrolineata* | - | - | - | None | sox15 - |
| Aves | *Cicinnurus regius* | - | + | - | None | sox15 - |
| Aves | *Ciconia boyciana* | - | + | - | None | sox15 - |
| Aves | *Ciconia maguari* | - | - | - | None | sox15 - |
| Aves | *Cinclus mexicanus* | - | + | - | None | sox15 - |
| Aves | *Circaetus pectoralis* | - | - | - | None | sox15 - |
| Aves | *Cisticola juncidis* | - | - | - | None | sox15 - |
| Aves | *Climacteris rufus* | - | + | - | None | sox15 - |
| Aves | *Cnemophilus loriae* | - | - | - | None | sox15 - |
| Aves | *Cochlearius cochlearius* | - | - | - | None | sox15 - |
| Aves | *Colinus virginianus* | - | - | - | None | sox15 - |
| Aves | *Colius striatus* | - | - | - | None | sox15 - |
| Aves | *Columba janthina* | - | - | - | None | sox15 - |
| Aves | *Columba livia* | - | - | - | None | sox15 - |
| Aves | *Columbina picui* | - | - | - | None | sox15 - |
| Aves | *Copsychus sechellarum* | - | - | - | None | sox15 - |
| Aves | *Corapipo altera* | - | - | - | None | sox15 - |
| Aves | *Corvus brachyrhynchos* | - | - | - | None | sox15 - |
| Aves | *Corvus cornix* | - | - | - | None | sox15 - |
| Aves | *Corvus hawaiiensis* | - | + | - | None | sox15 - |
| Aves | *Corvus macrorhynchos* | - | - | - | None | sox15 - |
| Aves | *Corvus monedula* | - | - | - | None | sox15 - |
| Aves | *Corvus moneduloides* | - | + | - | None | sox15 - |
| Aves | *Corythaeola cristata* | - | - | - | None | sox15 - |
| Aves | *Corythaixoides concolor* | - | - | - | None | sox15 - |
| Aves | *Coturnix japonica* | - | - | - | None | sox15 - |
| Aves | *Crotophaga sulcirostris* | - | - | - | None | sox15 - |
| Aves | *Crypturellus cinnamomeus* | - | + | - | None | sox15 - |
| Aves | *Crypturellus soui* | - | - | - | None | sox15 - |
| Aves | *Crypturellus undulatus* | - | - | - | None | sox15 - |
| Aves | *Cuculus canorus* | - | - | - | None | sox15 - |
| Aves | *Cyanistes caeruleus* | - | + | - | None | sox15 - |
| Aves | *Cyanoderma ruficeps* | - | + | - | None | sox15 - |
| Aves | *Cygnus atratus* | - | - | - | None | sox15 - |
| Aves | *Cygnus cygnus* | - | - | - | None | sox15 - |
| Aves | *Cygnus olor* | - | - | - | None | sox15 - |
| Aves | *Daphoenositta chrysoptera* | - | + | - | None | sox15 - |
| Aves | *Dasyornis broadbenti* | - | + | - | None | sox15 - |
| Aves | *Dendrocopos noguchii* | - | + | - | None | sox15 - |
| Aves | *Dicaeum eximium* | - | + | - | None | sox15 - |
| Aves | *Dicrurus megarhynchus* | - | + | - | None | sox15 - |
| Aves | *Diphyllodes magnificus* | - | + | - | None | sox15 - |
| Aves | *Donacobius atricapilla* | - | - | - | None | sox15 - |
| Aves | *Dromaius novaehollandiae* | - | - | - | None | sox15 - |
| Aves | *Dromas ardeola* | - | - | - | None | sox15 - |
| Aves | *Drymodes brunneopygia* | - | + | - | None | sox15 - |
| Aves | *Dryoscopus gambensis* | - | + | - | None | sox15 - |
| Aves | *Dyaphorophyia castanea* | - | - | - | None | sox15 - |
| Aves | *Eclectus roratus* | - | - | - | None | sox15 - |
| Aves | *Edolisoma coerulescens* | - | + | - | None | sox15 - |
| Aves | *Egretta garzetta* | - | - | - | None | sox15 - |
| Aves | *Emberiza fucata* | - | - | - | None | sox15 - |
| Aves | *Empidonax traillii* | - | - | - | None | sox15 - |
| Aves | *Eolophus roseicapillus* | - | - | - | None | sox15 - |
| Aves | *Eopsaltria australis* | - | + | - | None | sox15 - |
| Aves | *Eremophila alpestris* | - | - | - | None | sox15 - |
| Aves | *Eremophila alpestris peregrina* | - | + | - | None | sox15 - |
| Aves | *Erithacus rubecula* | - | - | - | None | sox15 - |
| Aves | *Erpornis zantholeuca* | - | + | - | None | sox15 - |
| Aves | *Erythrocercus mccallii* | - | + | - | None | sox15 - |
| Aves | *Erythrura gouldiae* | - | - | - | None | sox15 - |
| Aves | *Eubucco bourcierii* | - | - | - | None | sox15 - |
| Aves | *Eudromia elegans* | - | - | - | None | sox15 - |
| Aves | *Eudyptes chrysocome* | - | - | - | None | sox15 - |
| Aves | *Eudyptes chrysolophus* | - | - | - | None | sox15 - |
| Aves | *Eudyptes filholi* | - | - | - | None | sox15 - |
| Aves | *Eudyptes moseleyi* | - | - | - | None | sox15 - |
| Aves | *Eudyptes pachyrhynchus* | - | + | - | None | sox15 - |
| Aves | *Eudyptes robustus* | - | + | - | None | sox15 - |
| Aves | *Eudyptes schlegeli* | - | + | - | None | sox15 - |
| Aves | *Eudyptes sclateri* | - | - | - | None | sox15 - |
| Aves | *Eudyptula albosignata* | - | - | - | None | sox15 - |
| Aves | *Eudyptula minor* | - | - | - | None | sox15 - |
| Aves | *Eudyptula novaehollandiae* | - | - | - | None | sox15 - |
| Aves | *Eulacestoma nigropectus* | - | + | - | None | sox15 - |
| Aves | *Eupsittula pertinax* | - | + | - | None | sox15 - |
| Aves | *Eurypyga helias* | - | - | - | None | sox15 - |
| Aves | *Eurystomus gularis* | - | - | - | None | sox15 - |
| Aves | *Falco cherrug* | - | - | - | None | sox15 - |
| Aves | *Falco peregrinus* | - | - | - | None | sox15 - |
| Aves | *Falco rusticolus* | - | - | - | None | sox15 - |
| Aves | *Falco tinnunculus* | - | - | - | None | sox15 - |
| Aves | *Falcunculus frontatus* | - | + | - | None | sox15 - |
| Aves | *Ficedula albicollis* | - | - | - | None | sox15 - |
| Aves | *Formicarius rufipectus* | - | - | - | None | sox15 - |
| Aves | *Fratercula cirrhata* | - | - | - | None | sox15 - |
| Aves | *Fregata magnificens* | - | - | - | None | sox15 - |
| Aves | *Fregetta grallaria* | - | - | - | None | sox15 - |
| Aves | *Fringilla coelebs* | - | - | - | None | sox15 - |
| Aves | *Fulica atra* | - | - | - | None | sox15 - |
| Aves | *Fulmarus glacialis* | - | - | - | None | sox15 - |
| Aves | *Furnarius figulus* | - | - | - | None | sox15 - |
| Aves | *Galbula dea* | - | - | - | None | sox15 - |
| Aves | *Gallirallus okinawae* | - | - | - | None | sox15 - |
| Aves | *Gallus gallus* | - | - | - | None | sox15 - |
| Aves | *Gavia stellata* | - | - | - | None | sox15 - |
| Aves | *Geococcyx californianus* | - | - | - | None | sox15 - |
| Aves | *Geospiza fortis* | - | + | - | None | sox15 - |
| Aves | *Geothlypis trichas* | - | - | - | None | sox15 - |
| Aves | *Glareola pratincola* | - | - | - | None | sox15 - |
| Aves | *Glaucidium brasilianum* | - | - | - | None | sox15 - |
| Aves | *Grallaria varia* | - | - | - | None | sox15 - |
| Aves | *Grantiella picta* | - | + | - | None | sox15 - |
| Aves | *Grus americana* | - | - | - | None | sox15 - |
| Aves | *Grus japonensis* | - | - | - | None | sox15 - |
| Aves | *Grus monacha* | - | + | - | None | sox15 - |
| Aves | *Grus nigricollis* | - | - | - | None | sox15 - |
| Aves | *Guaruba guarouba* | - | - | - | None | sox15 - |
| Aves | *Gymnorhina tibicen* | - | - | - | None | sox15 - |
| Aves | *Halcyon senegalensis* | - | - | - | None | sox15 - |
| Aves | *Haliaeetus albicilla* | - | - | - | None | sox15 - |
| Aves | *Haliaeetus leucocephalus* | - | - | - | None | sox15 - |
| Aves | *Haliaeetus pelagicus* | - | + | - | None | sox15 - |
| Aves | *Heliornis fulica* | - | - | - | None | sox15 - |
| Aves | *Hemignathus virens* | - | + | - | None | sox15 - |
| Aves | *Hemignathus wilsoni* | - | + | - | None | sox15 - |
| Aves | *Hemiprocne comata* | - | - | - | None | sox15 - |
| Aves | *Herpetotheres cachinnans* | - | - | - | None | sox15 - |
| Aves | *Heteronetta atricapilla* | - | - | - | None | sox15 - |
| Aves | *Himantopus himantopus* | - | - | - | None | sox15 - |
| Aves | *Hippolais icterina* | - | + | - | None | sox15 - |
| Aves | *Hirundo rustica* | - | - | - | None | sox15 - |
| Aves | *Horornis vulcanius* | - | - | - | None | sox15 - |
| Aves | *Hydrobates tethys* | - | - | - | None | sox15 - |
| Aves | *Hylia prasina* | - | - | - | None | sox15 - |
| Aves | *Hypocryptadius cinnamomeus* | - | + | - | None | sox15 - |
| Aves | *Ibidorhyncha struthersii* | - | - | - | None | sox15 - |
| Aves | *Ifrita kowaldi* | - | + | - | None | sox15 - |
| Aves | *Illadopsis cleaveri* | - | - | - | None | sox15 - |
| Aves | *Indicator maculatus* | - | + | - | None | sox15 - |
| Aves | *Irena cyanogastra* | - | + | - | None | sox15 - |
| Aves | *Jacana jacana* | - | - | - | None | sox15 - |
| Aves | *Junco hyemalis* | - | + | - | None | sox15 - |
| Aves | *Lagopus muta japonica* | - | + | - | None | sox15 - |
| Aves | *Lamprotornis superbus* | - | - | - | None | sox15 - |
| Aves | *Lanius ludovicianus* | - | + | - | None | sox15 - |
| Aves | *Larus smithsonianus* | - | - | - | None | sox15 - |
| Aves | *Leiothrix lutea* | - | - | - | None | sox15 - |
| Aves | *Lepidothrix coronata* | - | - | - | None | sox15 - |
| Aves | *Leptocoma aspasia* | - | - | - | None | sox15 - |
| Aves | *Leptosomus discolor* | - | - | - | None | sox15 - |
| Aves | *Leucopsar rothschildi* | - | - | - | None | sox15 - |
| Aves | *Lichenostomus cassidix* | - | + | - | None | sox15 - |
| Aves | *Limosa lapponica* | - | - | - | None | sox15 - |
| Aves | *Locustella ochotensis* | - | - | - | None | sox15 - |
| Aves | *Lonchura striata domestica* | - | + | - | None | sox15 - |
| Aves | *Lophotis ruficrista* | - | - | - | None | sox15 - |
| Aves | *Lorius garrulus* | - | + | - | None | sox15 - |
| Aves | *Loxia curvirostra* | - | + | - | None | sox15 - |
| Aves | *Loxia leucoptera* | - | + | - | None | sox15 - |
| Aves | *Lycocorax pyrrhopterus* | - | - | - | None | sox15 - |
| Aves | *Lyrurus tetrix* | - | - | - | None | sox15 - |
| Aves | *Machaerirhynchus nigripectus* | - | - | - | None | sox15 - |
| Aves | *Malurus cyaneus* | - | - | - | None | sox15 - |
| Aves | *Malurus elegans* | - | + | - | None | sox15 - |
| Aves | *Manacus vitellinus* | - | - | - | None | sox15 - |
| Aves | *Megadyptes antipodes* | - | - | - | None | sox15 - |
| Aves | *Melanerpes aurifrons* | - | + | - | None | sox15 - |
| Aves | *Melanocharis versteri* | - | - | - | None | sox15 - |
| Aves | *Meleagris gallopavo* | - | - | - | None | sox15 - |
| Aves | *Melopsittacus undulatus* | - | + | - | None | sox15 - |
| Aves | *Melospiza melodia* | - | + | - | None | sox15 - |
| Aves | *Menura novaehollandiae* | - | - | - | None | sox15 - |
| Aves | *Merops nubicus* | - | - | - | None | sox15 - |
| Aves | *Mesembrinibis cayennensis* | - | - | - | None | sox15 - |
| Aves | *Mesitornis unicolor* | - | - | - | None | sox15 - |
| Aves | *Mionectes macconnelli* | - | - | - | None | sox15 - |
| Aves | *Mixornis gularis* | - | - | - | None | sox15 - |
| Aves | *Mohoua ochrocephala* | - | - | - | None | sox15 - |
| Aves | *Molothrus ater* | - | - | - | None | sox15 - |
| Aves | *Motacilla alba* | - | - | - | None | sox15 - |
| Aves | *Myiagra hebetior* | - | - | - | None | sox15 - |
| Aves | *Myiopsitta monachus* | - | + | - | None | sox15 - |
| Aves | *Mystacornis crossleyi* | - | - | - | None | sox15 - |
| Aves | *Nannopterum auritus* | - | + | - | None | sox15 - |
| Aves | *Nannopterum brasilianus* | - | + | - | None | sox15 - |
| Aves | *Nannopterum harrisi* | - | + | - | None | sox15 - |
| Aves | *Neodrepanis coruscans* | - | - | - | None | sox15 - |
| Aves | *Neopelma chrysocephalum* | - | - | - | None | sox15 - |
| Aves | *Neopipo cinnamomea* | - | - | - | None | sox15 - |
| Aves | *Nesospiza acunhae* | - | + | - | None | sox15 - |
| Aves | *Nestor notabilis* | - | + | - | None | sox15 - |
| Aves | *Nettapus auritus* | - | - | - | None | sox15 - |
| Aves | *Nicator chloris* | - | + | - | None | sox15 - |
| Aves | *Nipponia nippon* | - | - | - | None | sox15 - |
| Aves | *Nisaetus nipalensis* | - | + | - | None | sox15 - |
| Aves | *Nothocercus julius* | - | - | - | None | sox15 - |
| Aves | *Nothocercus nigrocapillus* | - | - | - | None | sox15 - |
| Aves | *Nothoprocta ornata* | - | - | - | None | sox15 - |
| Aves | *Nothoprocta pentlandii* | - | - | - | None | sox15 - |
| Aves | *Nothoprocta perdicaria* | - | - | - | None | sox15 - |
| Aves | *Notiomystis cincta* | - | + | - | None | sox15 - |
| Aves | *Numida meleagris* | - | - | - | None | sox15 - |
| Aves | *Nyctibius bracteatus* | - | - | - | None | sox15 - |
| Aves | *Nyctibius grandis* | - | - | - | None | sox15 - |
| Aves | *Nycticryphes semicollaris* | - | - | - | None | sox15 - |
| Aves | *Nyctiprogne leucopyga* | - | - | - | None | sox15 - |
| Aves | *Nymphicus hollandicus* | - | + | - | None | sox15 - |
| Aves | *Oceanites oceanicus* | - | - | - | None | sox15 - |
| Aves | *Odontophorus gujanensis* | - | - | - | None | sox15 - |
| Aves | *Oenanthe oenanthe* | - | + | - | None | sox15 - |
| Aves | *Onychorhynchus coronatus* | - | + | - | None | sox15 - |
| Aves | *Opisthocomus hoazin* | - | - | - | None | sox15 - |
| Aves | *Oreocharis arfaki* | - | + | - | None | sox15 - |
| Aves | *Oreotrochilus melanogaster* | - | - | - | None | sox15 - |
| Aves | *Origma solitaria* | - | + | - | None | sox15 - |
| Aves | *Oriolus oriolus* | - | - | - | None | sox15 - |
| Aves | *Orthonyx spaldingii* | - | + | - | None | sox15 - |
| Aves | *Otus sunia* | - | - | - | None | sox15 - |
| Aves | *Oxylabes madagascariensis* | - | - | - | None | sox15 - |
| Aves | *Oxyruncus cristatus* | - | - | - | None | sox15 - |
| Aves | *Oxyura jamaicensis* | - | - | - | None | sox15 - |
| Aves | *Pachycephala philippinensis* | - | - | - | None | sox15 - |
| Aves | *Pachyramphus minor* | - | - | - | None | sox15 - |
| Aves | *Pandion haliaetus* | - | - | - | None | sox15 - |
| Aves | *Panurus biarmicus* | - | - | - | None | sox15 - |
| Aves | *Paradisaea raggiana* | - | + | - | None | sox15 - |
| Aves | *Paradisaea rubra* | - | + | - | None | sox15 - |
| Aves | *Paradoxornis webbianus* | - | - | - | None | sox15 - |
| Aves | *Pardalotus punctatus* | - | - | - | None | sox15 - |
| Aves | *Parotia lawesii* | - | + | - | None | sox15 - |
| Aves | *Parus major* | - | - | - | None | sox15 - |
| Aves | *Passer domesticus* | - | + | - | None | sox15 - |
| Aves | *Passer montanus* | - | + | - | None | sox15 - |
| Aves | *Passerina amoena* | - | - | - | None | sox15 - |
| Aves | *Patagioenas fasciata monilis* | - | + | - | None | sox15 - |
| Aves | *Pavo cristatus* | - | + | - | None | sox15 - |
| Aves | *Pedionomus torquatus* | - | - | - | None | sox15 - |
| Aves | *Pelecanoides urinatrix* | - | - | - | None | sox15 - |
| Aves | *Pelecanus crispus* | - | - | - | None | sox15 - |
| Aves | *Penelope pileata* | - | - | - | None | sox15 - |
| Aves | *Peucedramus taeniatus* | - | + | - | None | sox15 - |
| Aves | *Phaethon lepturus* | - | - | - | None | sox15 - |
| Aves | *Phaetusa simplex* | - | - | - | None | sox15 - |
| Aves | *Phainopepla nitens* | - | + | - | None | sox15 - |
| Aves | *Phalacrocorax carbo* | - | - | - | None | sox15 - |
| Aves | *Phalacrocorax pelagicus* | - | + | - | None | sox15 - |
| Aves | *Phasianus colchicus* | - | + | - | None | sox15 - |
| Aves | *Pheucticus melanocephalus* | - | - | - | None | sox15 - |
| Aves | *Phoebastria albatrus* | - | - | - | None | sox15 - |
| Aves | *Phoenicopterus ruber* | - | - | - | None | sox15 - |
| Aves | *Phylloscopus plumbeitarsus* | - | + | - | None | sox15 - |
| Aves | *Phylloscopus trochiloides viridanus* | - | + | - | None | sox15 - |
| Aves | *Phylloscopus trochilus acredula* | - | + | - | None | sox15 - |
| Aves | *Piaya cayana* | - | - | - | None | sox15 - |
| Aves | *Picathartes gymnocephalus* | - | + | - | None | sox15 - |
| Aves | *Picoides pubescens* | - | - | - | None | sox15 - |
| Aves | *Pionus senilis* | - | + | - | None | sox15 - |
| Aves | *Pipra filicauda* | - | + | - | None | sox15 - |
| Aves | *Piprites chloris* | - | - | - | None | sox15 - |
| Aves | *Pitta sordida* | - | - | - | None | sox15 - |
| Aves | *Ploceus nigricollis* | - | + | - | None | sox15 - |
| Aves | *Pluvianellus socialis* | - | - | - | None | sox15 - |
| Aves | *Podargus strigoides* | - | - | - | None | sox15 - |
| Aves | *Podiceps cristatus* | - | - | - | None | sox15 - |
| Aves | *Podilymbus podiceps* | - | - | - | None | sox15 - |
| Aves | *Poecile atricapillus* | - | - | - | None | sox15 - |
| Aves | *Pogoniulus pusillus* | - | + | - | None | sox15 - |
| Aves | *Polioptila caerulea* | - | - | - | None | sox15 - |
| Aves | *Pomatorhinus ruficollis* | - | - | - | None | sox15 - |
| Aves | *Pomatostomus ruficeps* | - | + | - | None | sox15 - |
| Aves | *Probosciger aterrimus* | - | - | - | None | sox15 - |
| Aves | *Promerops cafer* | - | - | - | None | sox15 - |
| Aves | *Prunella fulvescens* | - | + | - | None | sox15 - |
| Aves | *Prunella himalayana* | - | - | - | None | sox15 - |
| Aves | *Pseudopodoces humilis* | - | + | - | None | sox15 - |
| Aves | *Pseudorectes ferrugineus* | - | + | - | None | sox15 - |
| Aves | *Psilopogon haemacephalus* | - | - | - | None | sox15 - |
| Aves | *Psittacula krameri* | - | - | - | None | sox15 - |
| Aves | *Psittacus erithacus* | - | + | - | None | sox15 - |
| Aves | *Psittacus timneh* | - | - | - | None | sox15 - |
| Aves | *Psitteuteles goldiei* | - | + | - | None | sox15 - |
| Aves | *Psophia crepitans* | - | - | - | None | sox15 - |
| Aves | *Pterocles burchelli* | - | - | - | None | sox15 - |
| Aves | *Pterocles gutturalis* | - | - | - | None | sox15 - |
| Aves | *Pterocles orientalis* | - | - | - | None | sox15 - |
| Aves | *Pterocnemia pennata* | - | + | - | None | sox15 - |
| Aves | *Pteruthius melanotis* | - | - | - | None | sox15 - |
| Aves | *Ptilonorhynchus violaceus* | - | - | - | None | sox15 - |
| Aves | *Ptilorrhoa leucosticta* | - | - | - | None | sox15 - |
| Aves | *Pycnonotus jocosus* | - | - | - | None | sox15 - |
| Aves | *Pygoscelis adeliae* | - | - | - | None | sox15 - |
| Aves | *Pygoscelis antarcticus* | - | - | - | None | sox15 - |
| Aves | *Pygoscelis papua* | - | - | - | None | sox15 - |
| Aves | *Pyrrhura frontalis* | - | + | - | None | sox15 - |
| Aves | *Pyrrhura griseipectus* | - | - | - | None | sox15 - |
| Aves | *Pyrrhura lepida* | - | - | - | None | sox15 - |
| Aves | *Pyrrhura molinae* | - | - | - | None | sox15 - |
| Aves | *Pyrrhura perlata* | - | + | - | None | sox15 - |
| Aves | *Quiscalus mexicanus* | - | + | - | None | sox15 - |
| Aves | *Ramphastos sulfuratus* | - | - | - | None | sox15 - |
| Aves | *Recurvirostra avosetta* | - | - | - | None | sox15 - |
| Aves | *Regulus satrapa* | - | - | - | None | sox15 - |
| Aves | *Rhabdornis inornatus* | - | + | - | None | sox15 - |
| Aves | *Rhadina sibilatrix* | - | - | - | None | sox15 - |
| Aves | *Rhagologus leucostigma* | - | + | - | None | sox15 - |
| Aves | *Rhea americana* | - | - | - | None | sox15 - |
| Aves | *Rhegmatorhina hoffmannsi* | - | - | - | None | sox15 - |
| Aves | *Rhinopomastus cyanomelas* | - | + | - | None | sox15 - |
| Aves | *Rhinoptilus africanus* | - | - | - | None | sox15 - |
| Aves | *Rhipidura dahli* | - | + | - | None | sox15 - |
| Aves | *Rhodinocichla rosea* | - | - | - | None | sox15 - |
| Aves | *Rhynochetos jubatus* | - | - | - | None | sox15 - |
| Aves | *Rissa tridactyla* | - | - | - | None | sox15 - |
| Aves | *Rostratula benghalensis* | - | - | - | None | sox15 - |
| Aves | *Rynchops niger* | - | - | - | None | sox15 - |
| Aves | *Sagittarius serpentarius* | - | - | - | None | sox15 - |
| Aves | *Sakesphorus luctuosus* | - | - | - | None | sox15 - |
| Aves | *Sapayoa aenigma* | - | - | - | None | sox15 - |
| Aves | *Saxicola maurus maurus* | - | + | - | None | sox15 - |
| Aves | *Sclerurus mexicanus* | - | - | - | None | sox15 - |
| Aves | *Scolopax mira* | - | + | - | None | sox15 - |
| Aves | *Scopus umbretta* | - | - | - | None | sox15 - |
| Aves | *Scytalopus superciliaris* | - | - | - | None | sox15 - |
| Aves | *Semnornis frantzii* | - | + | - | None | sox15 - |
| Aves | *Serilophus lunatus* | - | - | - | None | sox15 - |
| Aves | *Serinus canaria* | - | - | - | None | sox15 - |
| Aves | *Setophaga coronata* | - | - | - | None | sox15 - |
| Aves | *Setophaga kirtlandii* | - | - | - | None | sox15 - |
| Aves | *Sialia sialis* | - | - | - | None | sox15 - |
| Aves | *Sitta europaea* | - | + | - | None | sox15 - |
| Aves | *Smithornis capensis* | - | - | - | None | sox15 - |
| Aves | *Spelaeornis formosus* | - | - | - | None | sox15 - |
| Aves | *Spheniscus demersus* | - | - | - | None | sox15 - |
| Aves | *Spheniscus humboldti* | - | - | - | None | sox15 - |
| Aves | *Spheniscus magellanicus* | - | - | - | None | sox15 - |
| Aves | *Spheniscus mendiculus* | - | - | - | None | sox15 - |
| Aves | *Spizaetus tyrannus* | - | - | - | None | sox15 - |
| Aves | *Spizella passerina* | - | + | - | None | sox15 - |
| Aves | *Sporophila hypoxantha* | - | + | - | None | sox15 - |
| Aves | *Steatornis caripensis* | - | - | - | None | sox15 - |
| Aves | *Stercorarius parasiticus* | - | - | - | None | sox15 - |
| Aves | *Sterna hirundo* | - | - | - | None | sox15 - |
| Aves | *Sterrhoptilus dennistouni* | - | + | - | None | sox15 - |
| Aves | *Stictonetta naevosa* | - | - | - | None | sox15 - |
| Aves | *Streptopelia turtur* | - | - | - | None | sox15 - |
| Aves | *Strigops habroptila* | - | + | - | None | sox15 - |
| Aves | *Strix occidentalis* | - | - | - | None | sox15 - |
| Aves | *Struthidea cinerea* | - | + | - | None | sox15 - |
| Aves | *Struthio camelus* | - | - | - | None | sox15 - |
| Aves | *Sturnus vulgaris* | - | - | - | None | sox15 - |
| Aves | *Sula dactylatra* | - | - | - | None | sox15 - |
| Aves | *Sylvia atricapilla* | - | - | - | None | sox15 - |
| Aves | *Sylvia borin* | - | - | - | None | sox15 - |
| Aves | *Sylvietta virens* | - | - | - | None | sox15 - |
| Aves | *Syrmaticus mikado* | - | + | - | None | sox15 - |
| Aves | *Syrrhaptes paradoxus* | - | - | - | None | sox15 - |
| Aves | *Tachuris rubrigastra* | - | - | - | None | sox15 - |
| Aves | *Tachycineta bicolor* | - | + | - | None | sox15 - |
| Aves | *Taeniopygia guttata* | - | - | - | None | sox15 - |
| Aves | *Tauraco erythrolophus* | - | - | - | None | sox15 - |
| Aves | *Tetrax tetrax* | - | - | - | None | sox15 - |
| Aves | *Thalassarche chlororhynchos* | - | - | - | None | sox15 - |
| Aves | *Thinocorus orbignyianus* | - | - | - | None | sox15 - |
| Aves | *Thryothorus ludovicianus* | - | - | - | None | sox15 - |
| Aves | *Tichodroma muraria* | - | + | - | None | sox15 - |
| Aves | *Tinamus guttatus* | - | - | - | None | sox15 - |
| Aves | *Todus mexicanus* | - | - | - | None | sox15 - |
| Aves | *Toxostoma redivivum* | - | + | - | None | sox15 - |
| Aves | *Tricholaema leucomelas* | - | + | - | None | sox15 - |
| Aves | *Trogon melanurus* | - | - | - | None | sox15 - |
| Aves | *Turdus rufiventris* | - | + | - | None | sox15 - |
| Aves | *Turnix velox* | - | - | - | None | sox15 - |
| Aves | *Tympanuchus cupido pinnatus* | - | + | - | None | sox15 - |
| Aves | *Tyrannus savana* | - | - | - | None | sox15 - |
| Aves | *Upupa epops* | - | - | - | None | sox15 - |
| Aves | *Uria aalge* | - | - | - | None | sox15 - |
| Aves | *Uria lomvia* | - | - | - | None | sox15 - |
| Aves | *Urocolius indicus* | - | - | - | None | sox15 - |
| Aves | *Urocynchramus pylzowi* | - | - | - | None | sox15 - |
| Aves | *Vidua chalybeata* | - | + | - | None | sox15 - |
| Aves | *Vidua macroura* | - | + | - | None | sox15 - |
| Aves | *Vireo altiloquus* | - | + | - | None | sox15 - |
| Aves | *Xiphorhynchus elegans* | - | - | - | None | sox15 - |
| Aves | *Zapornia atra* | - | - | - | None | sox15 - |
| Aves | *Zonotrichia albicollis* | - | - | - | None | sox15 - |
| Aves | *Zosterops borbonicus* | - | + | - | None | sox15 - |
| Aves | *Zosterops hypoxanthus* | - | + | - | None | sox15 - |
| Aves | *Zosterops lateralis* | - | - | - | None | sox15 - |
| Aves | *Zosterops pallidus* | - | + | - | None | sox15 - |

**Table S3. Genbank numbers and range of the genes used in the analyses**

| Species | Gene | Genbank | Range |
| --- | --- | --- | --- |
| *Amblyraja radiata* | *sox1* | XM_033023155.1 | 512-1537 |
| *Callorhinchus milii* | *sox1* | XM_007891764.1 | 108-1160 |
| *Chiloscyllium plagiosum* | *sox1* | CM012961.1 | 12899087-12901711 |
| *Chiloscyllium punctatum* | *sox1* | BEZZ01000638.1 | 417489-418532 |
| *Erpetoichthys calabaricus* | *sox1* | XM_028801232.1 | 208-1248 |
| *Gallus gallus* | *sox1* | NM_204333.1 | 209-1330 |
| *Geotrypetes seraphini* | *sox1* | XM_033950250.1 | 1-1152 |
| *Homo sapiens* | *sox1* | NM_005986.3 | 511-1686 |
| *Hydrolagus affinis* | *sox1* | JAAILG010004180.1 | 13345-15969 |
| *Lepisosteus oculatus* | *sox1* | XM_015364217.1 | 406-1437 |
| *Maylandia zebra* | *sox1* | XM_004573625.3 | 322-1380 |
| *Podarcis muralis* | *sox1* | XM_028728115.1 | 784-1962 |
| *Pristis pectinata* | *sox1* | CM019865.1 | 87868199-87869923 |
| *Scyliorhinus torazame* | *sox1* | BFAA01079915.1 | 319-1161 |
| *Andrias davidianus* | *sox15* | KJ623265.1 | 271-1128 |
| *Bos taurus* | *sox15* | NM_001192075.2 | 239-940 |
| *Canis lupus* | *sox15* | XM_003434613.4 | 431-1132 |
| *Chiloscyllium plagiosum* | *sox15* | CM013003.1 | 899095-901719 |
| *Chiloscyllium punctatum* | *sox15* | BEZZ01008499.1 | 3-830 |
| *Chrysemys picta bellii* | *sox15* | XM_005313716.3 | 173-790 |
| *Dasypus novemcinctus* | *sox15* | XM_004468741.3 | 425-1126 |
| *Geotrypetes seraphini* | *sox15* | XM_033925163.1 | 188-1078 |
| *Gopherus evgoodei* | *sox15* | XM_030548210.1 | 185-817 |
| *Homo sapiens* | *sox15* | NM_006942.2 | 419-1120 |
| *Lacerta agilis* | *sox15* | XM_033169758.1 | 7-810 |
| *Latimeria chalumnae* | *sox15* | NW_005820241.1 | 160414-159828,141203-140948 |
| *Loxodonta africana* | *sox15* | XM_003416759.2 | 766-1443 |
| *Microcaecilia unicolor* | *sox15* | XM_030186896.1 | 190-1080 |
| *Mus musculus* | *sox15* | NM_009235.2 | 337-1032 |
| *Pantherophis guttatus* | *sox15* | XM_034407244.1 | 67-804 |
| *Pelodiscus sinensis* | *sox15* | XM_014576967.2 | 802-1419 |
| *Phascolarctos cinereus* | *sox15* | XM_020966486.1 | 468-1118 |
| *Podarcis muralis* | *sox15* | XM_028703808.1 | 66-857 |
| *Pristis pectinata* | *sox15* | CM019891.1 | 13017081-13019705 |
| *Pyxicephalus adspersus* | *sox15* | CM016417.1 | 1996676-1996850,1997840-1999089 |
| *Rana catesbeiana* | *sox15* | KV934738.1 | 1174-2525,4128-4302 |
| *Rhinatrema bivittatum* | *sox15* | XM_029581823.1 | 37-930 |
| *Trachemys scripta* | *sox15* | XM_034756827.1 | 1-621 |
| *Vombatus ursinus* | *sox15* | XM_027857251.1 | 1502-2188 |
| *Xenopus laevis* | *sox15* | NM_001087732.1 | 132-722 |
| *Xenopus tropicalis* | *sox15* | NM_001126574.1 | 55-654 |
| *Zootoca vivipara* | *sox15* | XM_035137001.1 | 1-804 |
| *Erpetoichthys calabaricus* | *sox19* | XM_028798433.1 | 490-1383 |
| *Esox lucius* | *sox19* | XM_010899168.3 | 439-1338 |
| *Gadus morhua* | *sox19* | XM_030338203.1 | 458-1459 |
| *Lepisosteus oculatus* | *sox19* | XM_006627561.2 | 512-1423 |
| *Maylandia zebra* | *sox19* | XM_004575969.5 | 519-1442 |
| *Nothobranchius furzeri* | *sox19* | XM_015957776.1 | 1021-1947 |
| *Oryzias latipes* | *sox19* | XM_023966042.1 | 302-1210 |
| *Paralichthys olivaceus* | *sox19* | XM_020100199.1 | 434-1354 |
| *Parambassis ranga* | *sox19* | XM_028429300.1 | 299-1219 |
| *Carassius auratus* | *sox19a* | XM_026256716.1 | 303-1193 |
| *Chanos chanos* | *sox19a* | XM_030792735.1 | 1-894 |
| *Danio rerio* | *sox19a* | NM_130908.2 | 268-1161 |
| *Electrophorus electricus* | *sox19a* | XM_026998430.2 | 332-1234 |
| *Sinocyclocheilus anshuiensis* | *sox19a* | XM_016485152.1 | 262-1152 |
| *Carassius auratus* | *sox19b* | XM_026267054.1 | 311-1189 |
| *Chanos chanos* | *sox19b* | XM_030780317.1 | 1-888 |
| *Danio rerio* | *sox19b* | NM_131702.1 | 259-1140 |
| *Electrophorus electricus* | *sox19b* | XM_027015559.2 | 322-1203 |
| *Sinocyclocheilus anshuiensis* | *sox19b* | XM_016507136.1 | 298-1170 |
| *Danio rerio* | *sox1a* | NM_001002483.2 | 320-1330 |
| *Maylandia zebra* | *sox1a* | XM_004551260.2 | 331-1194 |
| *Oryzias latipes* | *sox1a* | NM_001164865.1 | 1-969 |
| *Danio rerio* | *sox1b* | NM_001037662.1 | 236-1258 |
| *Oryzias latipes* | *sox1b* | XM_023966307.1 | 220-1251 |
| *Amblyraja radiata* | *sox2* | XM_033032159.1 | 369-1091 |
| *Callorhinchus milii* | *sox2* | XM_007907767.1 | 77-1012 |
| *Chiloscyllium plagiosum* | *sox2* | CM012968.1 | 50331561-50334185 |
| *Chiloscyllium punctatum* | *sox2* | BEZZ01000024.1 | 5715351-5716271 |
| *Danio rerio* | *sox2* | NM_213118.1 | 224-1171 |
| *Erpetoichthys calabaricus* | *sox2* | XM_028794611.1 | 306-1253 |
| *Gallus gallus* | *sox2* | NM_205188.2 | 74-1012 |
| *Geotrypetes seraphini* | *sox2* | XM_033957568.1 | 288-1229 |
| *Homo sapiens* | *sox2* | NM_003106.4 | 437-1390 |
| *Hydrolagus affinis* | *sox2* | JAAILG010087923.1 | 1-1711 |
| *Lepisosteus oculatus* | *sox2* | XM_015360888.1 | 496-1443 |
| *Maylandia zebra* | *sox2* | XM_004562078.2 | 244-1206 |
| *Oryzias latipes* | *sox2* | NM_001278881.1 | 1-969 |
| *Podarcis muralis* | *sox2* | XM_028731643.1 | 699-1673 |
| *Pristis pectinata* | *sox2* | CM019860.1 | 91272862-91275486 |
| *Scyliorhinus torazame* | *sox2* | BFAA01003007.1 | 203443-204375 |
| *Amblyraja radiata* | *sox3* | XM_033030296.1 | 307-1227 |
| *Callorhinchus milii* | *sox3* | XM_007892868.1 | 27-983 |
| *Chiloscyllium plagiosum* | *sox3* | CM012970.1 | 77429639-77432263 |
| *Chiloscyllium punctatum* | *sox3* | BEZZ01000333.1 | 1812549-1813827 |
| *Danio rerio* | *sox3* | NM_001001811.2 | 190-1092 |
| *Erpetoichthys calabaricus* | *sox3* | XM_028815815.1 | 379-1299 |
| *Gallus gallus* | *sox3* | NM_204195.1 | 191-1141 |
| *Geotrypetes seraphini* | *sox3* | XM_033947002.1 | 46-957 |
| *Homo sapiens* | *sox3* | NM_005634.3 | 10-1350 |
| *Hydrolagus affinis* | *sox3* | JAAILG010039076.1 | 3-2152 |
| *Lepisosteus oculatus* | *sox3* | XM_006632889.2 | 239-1156 |
| *Maylandia zebra* | *sox3* | XM_014412645.3 | 341-1243 |
| *Oryzias latipes* | *sox3* | NM_001104764.1 | 192-1094 |
| *Podarcis muralis* | *sox3* | XM_028715451.1 | 159-1157 |
| *Pristis pectinata* | *sox3* | CM019862.1 | 99818616-99821240 |
| *Scyliorhinus torazame* | *sox3* | BFAA01003357.1 | 155304-156209 |
| *Petromyzon marinus* | *soxB1* | XM_032977917.1 | 491-1852 |
| *Saccoglossus kowalevskii* | *soxB1* | NM_001164905.1 | 129-1079 |
| *Strongylocentrotus purpuratus* | *soxB1* | NM_214474.1 | 35-1069 |
| *Branchiostoma floridae* | *soxB1a* | XM_035812966.1 | 109-846 |
| *Branchiostoma floridae* | *soxB1b* | XM_035812932.1 | 94-1026 |
| *Branchiostoma floridae* | *soxB1c* | XM_035836878.1 | 194-1162 |
| *Dermochelys coriacea* | *sox15* | XM_038386231.1 | 36-662 |
| *Terrapene carolina triunguis* | *sox15* | XM_029913060.1 | 118-690 |
| *Pelodiscus sinensis* | *sox15* | XM_014576967.2 | 802-1419 |
| *Tachyglossus aculeatus* | *Sox15* | XM_038768226.1 | 1-903 |
| *Ornithorhynchus anatinus* | *Sox15* | XM_039910884.1 | 1-816 |

**Table S4. Likelihood ratio test for ω values in Fig. 4**

| Null model | | | Alternative model | | | Likelihood ratio test* | | |
| --- | --- | --- | --- | --- | --- | --- | --- | --- |
| model | np | lnL | model | np | lnL | Δnp | 2ΔlnL | p value |
| ω*_1_* = ω*_2_* = ω*_3_* = ω*_15_*, ω_r.b._ | 205 | -7130.78 | ω*_1_*_,_ ω*_2_*, ω*_3_*, ω*_15_*, ω_r.b._ | 208 | -7116.75 | 3 | 28.07 | 3.6×10^-6^ |
| ω*_1_* = ω*_2_*, ω*_3_*, ω*_15_*, ω_r.b._ | 207 | -7118.18 | ω*_1_*_,_ ω*_2_*, ω*_3_*, ω*_15_*, ω_r.b._ | 208 | -7116.75 | 1 | 2.86 | 0.091 |
| ω*_1_* = ω*_3_*, ω*_2_*, ω*_15_*, ω_r.b._ | 207 | -7116.82 | ω*_1_*_,_ ω*_2_*, ω*_3_*, ω*_15_*, ω_r.b._ | 208 | -7116.75 | 1 | 0.14 | 0.71 |
| ω*_1_* = ω*_15_*, ω*_2_*, ω*_3_*, ω_r.b._ | 207 | -7122.38 | ω*_1_*_,_ ω*_2_*, ω*_3_*, ω*_15_*, ω_r.b._ | 208 | -7116.75 | 1 | 11.27 | 7.9×10^-4^ |
| ω*_1_*, ω*_2_* = ω*_3_*, ω*_15_*, ω_r.b._ | 207 | -7117.59 | ω*_1_*_,_ ω*_2_*, ω*_3_*, ω*_15_*, ω_r.b._ | 208 | -7116.75 | 1 | 1.69 | 0.20 |
| ω*_1_*, ω*_2_* = ω*_15_*, ω*_3_*, ω_r.b._ | 207 | -7126.01 | ω*_1_*_,_ ω*_2_*, ω*_3_*, ω*_15_*, ω_r.b._ | 208 | -7116.75 | 1 | 18.52 | 1.7×10^-5^ |
| ω*_1_*, ω*_2_*, ω*_3_* = ω*_15_*, ω_r.b._ | 207 | -7122.83 | ω*_1_*_,_ ω*_2_*, ω*_3_*, ω*_15_*, ω_r.b._ | 208 | -7116.75 | 1 | 12.18 | 4.9×10^-4^ |
| ω_Cho_ = ω_Act_ = ω_Sar_, ω_r.b._ | 205 | -7122.68 | ω_Cho_, ω_Act_, ω_Sar_, ω_r.b._ | 207 | -7090.36 | 2 | 64.63 | 9.3×10^-15^ |
| ω_Cho_ = ω_Act_, ω_Sar_, ω_r.b._ | 206 | -7096.14 | ω_Cho_, ω_Act_, ω_Sar_, ω_r.b._ | 207 | -7090.36 | 1 | 11.55 | 6.8×10^-4^ |
| ω_Cho_ = ω_Sar_, ω_Act_, ω_r.b._ | 206 | -7090.41 | ω_Cho_, ω_Act_, ω_Sar_, ω_r.b._ | 207 | -7090.36 | 1 | 0.09 | 0.76 |
| ω_Cho_, ω_Act_ = ω_Sar_, ω_r.b._ | 206 | -7121.51 | ω_Cho_, ω_Act_, ω_Sar_, ω_r.b._ | 207 | -7090.36 | 1 | 62.29 | 3.0×10^-15^ |
| ω_Sar1_ = ω_Sar2_ = ω_Sar3_, ω_r.b._ | 205 | -7098.79 | ω_Sar1_, ω_Sar2_, ω_Sar3_, ω_r.b._ | 207 | -7091.75 | 2 | 14.09 | 8.8×10^-4^ |
| ω_Sar1_ = ω_Sar2_, ω_Sar3_, ω_r.b._ | 206 | -7092.24 | ω_Sar1_, ω_Sar2_, ω_Sar3_, ω_r.b._ | 207 | -7091.75 | 1 | 0.99 | 0.32 |
| ω_Sar1_ = ω_Sar3_, ω_Sar2_, ω_r.b._ | 206 | -7097.57 | ω_Sar1_, ω_Sar2_, ω_Sar3_, ω_r.b._ | 207 | -7091.75 | 1 | 11.64 | 6.5×10^-4^ |
| ω_Sar1_, ω_Sar2_ = ω_Sar3_, ω_r.b._ | 206 | -7094.56 | ω_Sar1_, ω_Sar2_, ω_Sar3_, ω_r.b._ | 207 | -7091.75 | 1 | 5.62 | 0.018 |

*p values were calculated using χ^2^ tests for each 2ΔlnL. Δnp is the degree of freedom for each test. np, number of parameters; lnL, log likelihood; ω_r.b._, ω value of remaining branches for each model

**
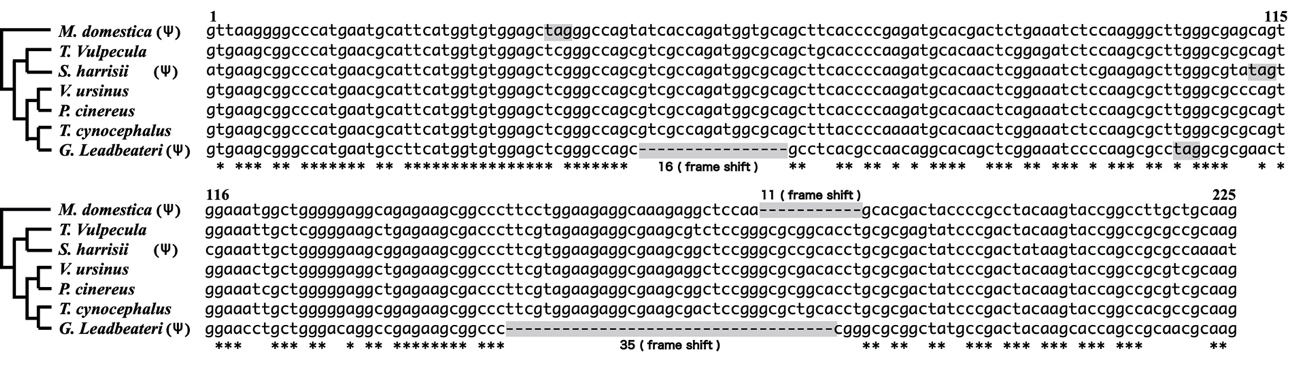
**

**Figure S1. Multiple alignment of the HMG box-encoding nucleotide sequences of marsupial pseudogenized and non-pseudogenized.**

***sox15*** Independent pseudogenization of *sox15* during marsupial speciation. (ψ) denotes the pseudogenization of *sox15* in the three species. Nucleotide sequences in the HMG box-coding region consisting of 225 nucleotides are shown in the alignment. In-frame stop codons and deletions with frameshift mutations are highlighted by gray boxes. Identical nucleotides among the seven species are indicated by asterisks in the alignment.

**
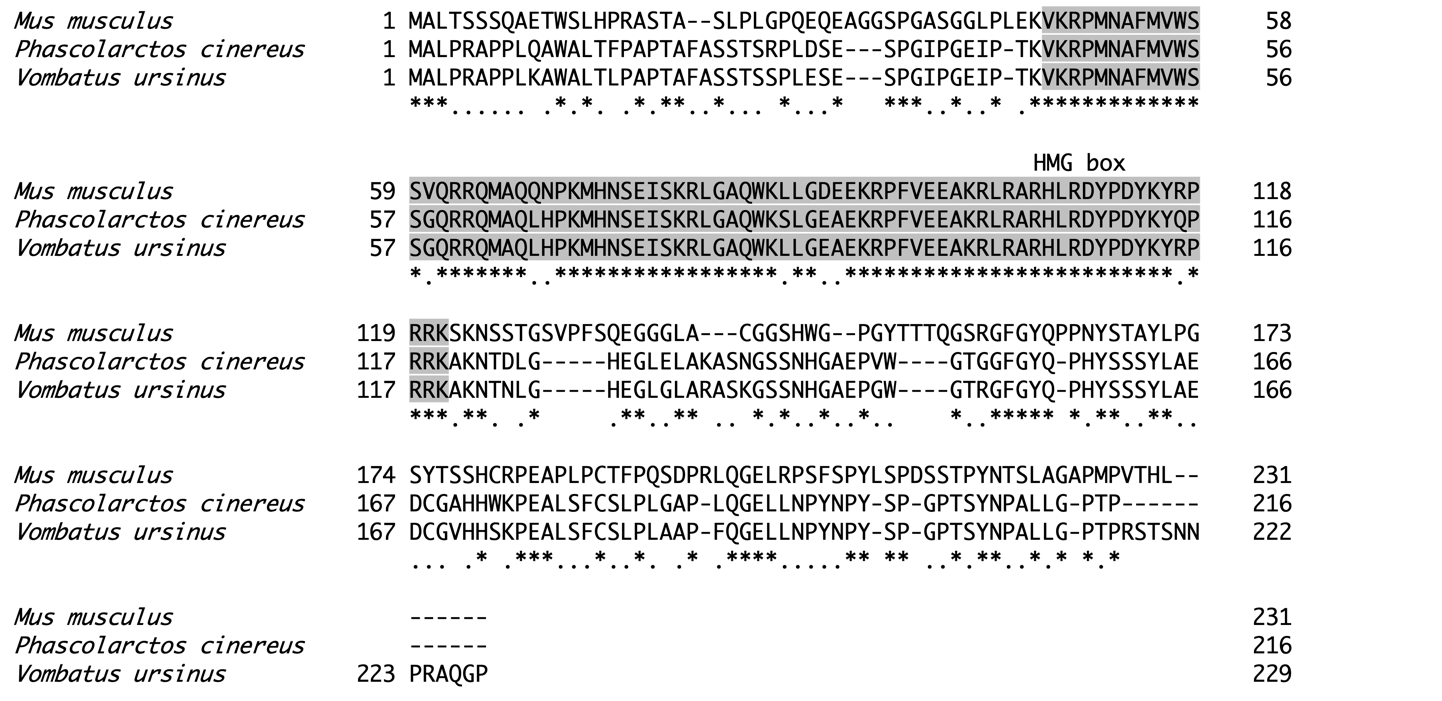
**

**Figure S2. Predicted amino acid sequences of marsupial SOX15 from koala (*Phascolarctos cinereus*) and wombat (*Vombatus ursinus*).**

Amino acid sequences of SOX15 from the two marsupial species and eutherian mouse *Mus musculus* were aligned by MAFFT version 7.427 and compared by genetics MAC ver 18. Asterisks indicate identical amino acids between the three proteins. SOX15 from the two marsupials had open reading frames consisting of 216 and 229 amino acid residues, which showed no evidence of pseudogenization.


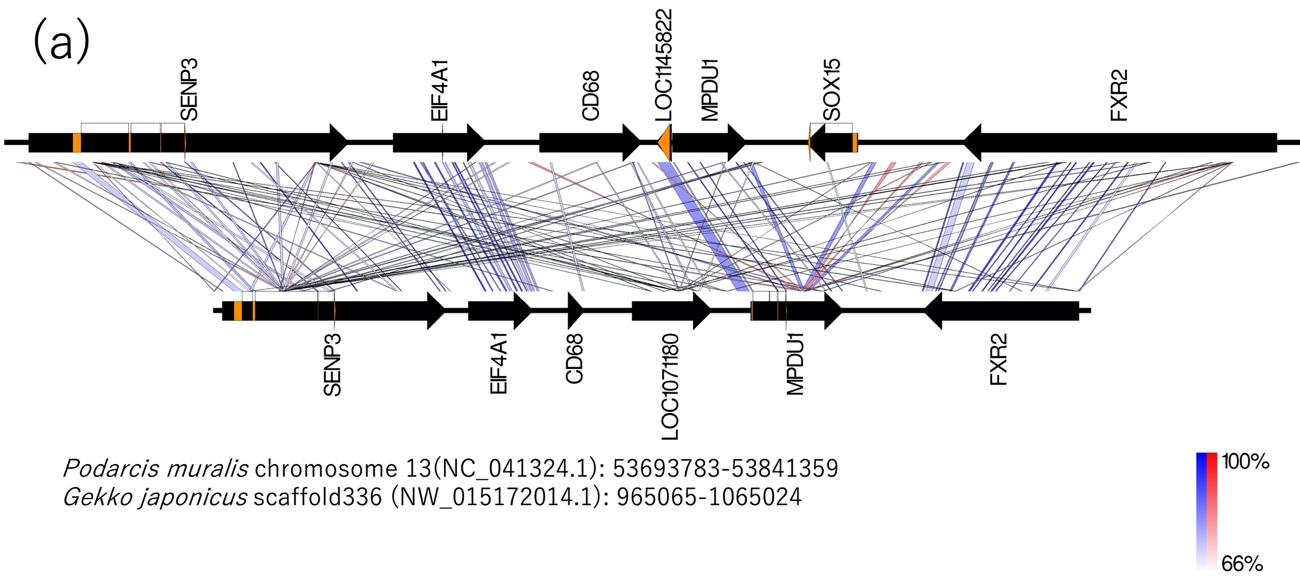

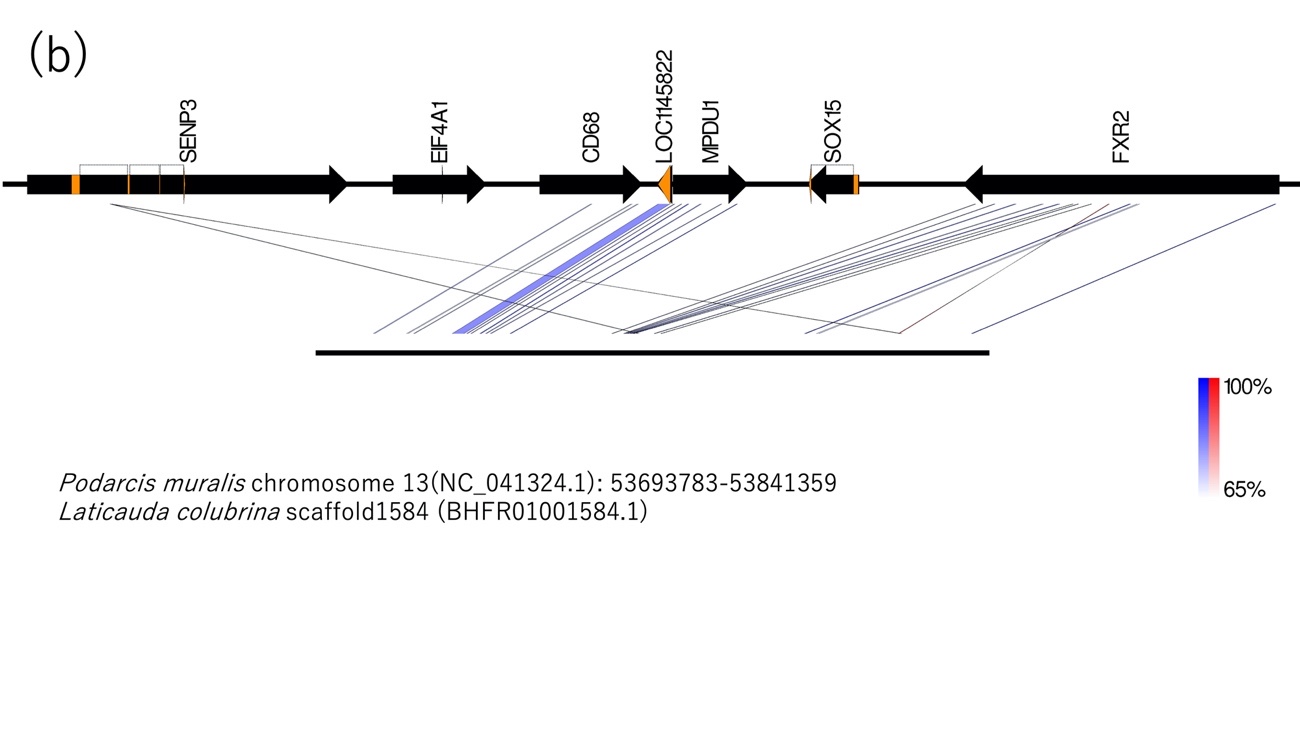

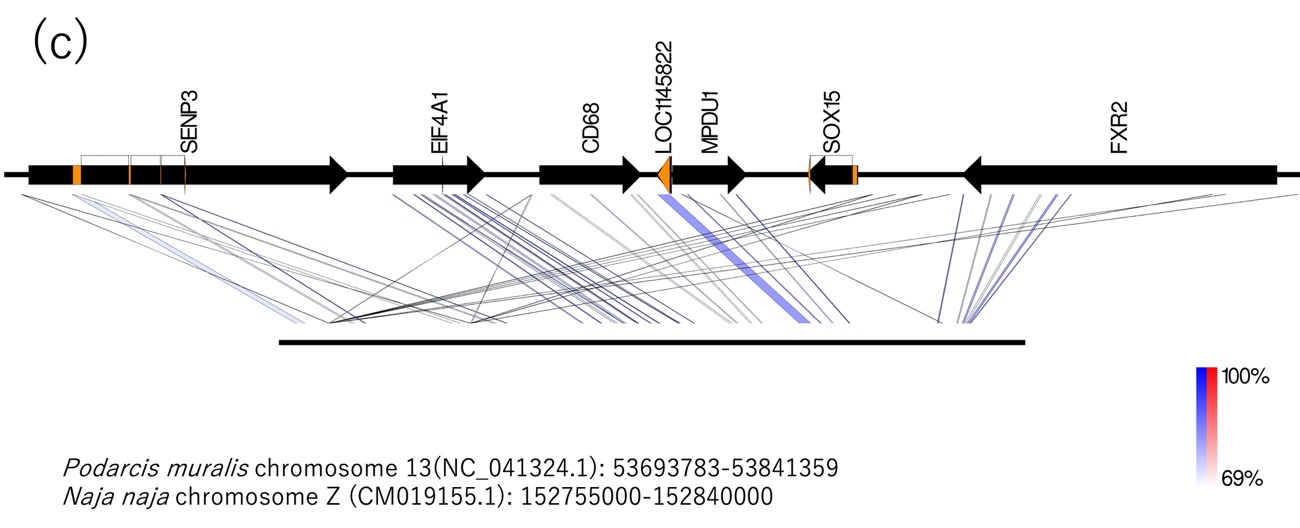

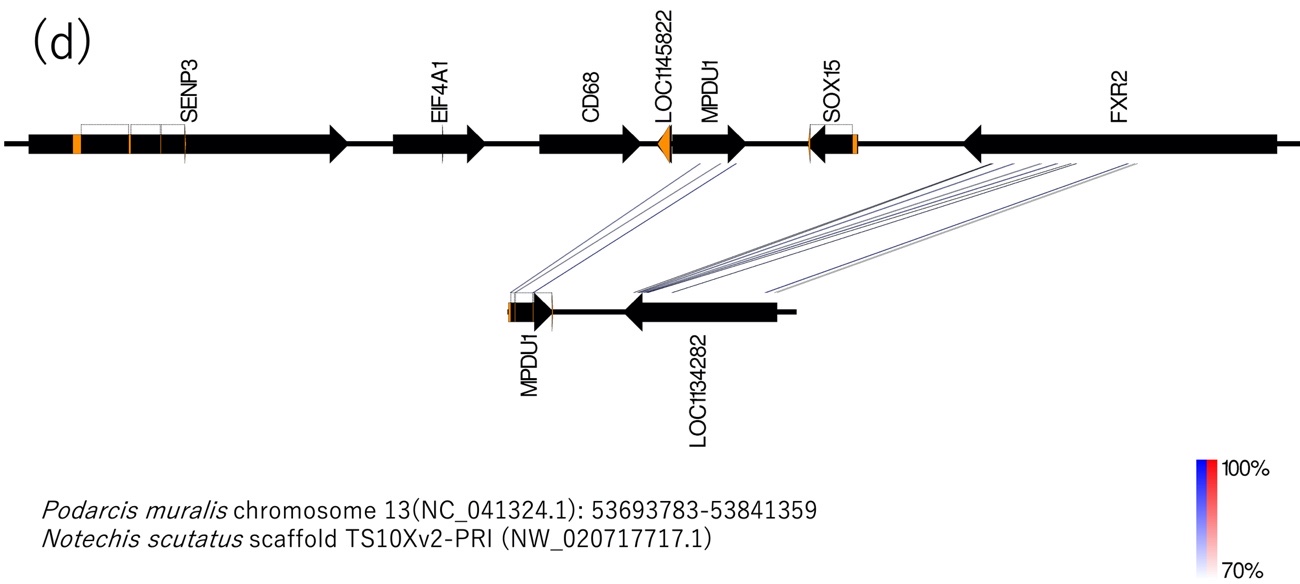

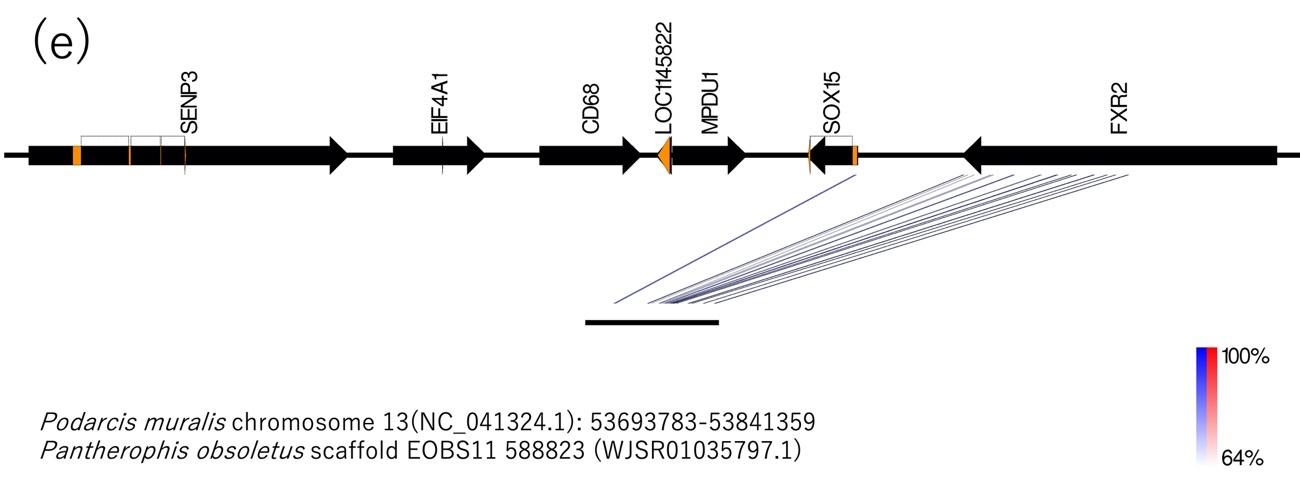

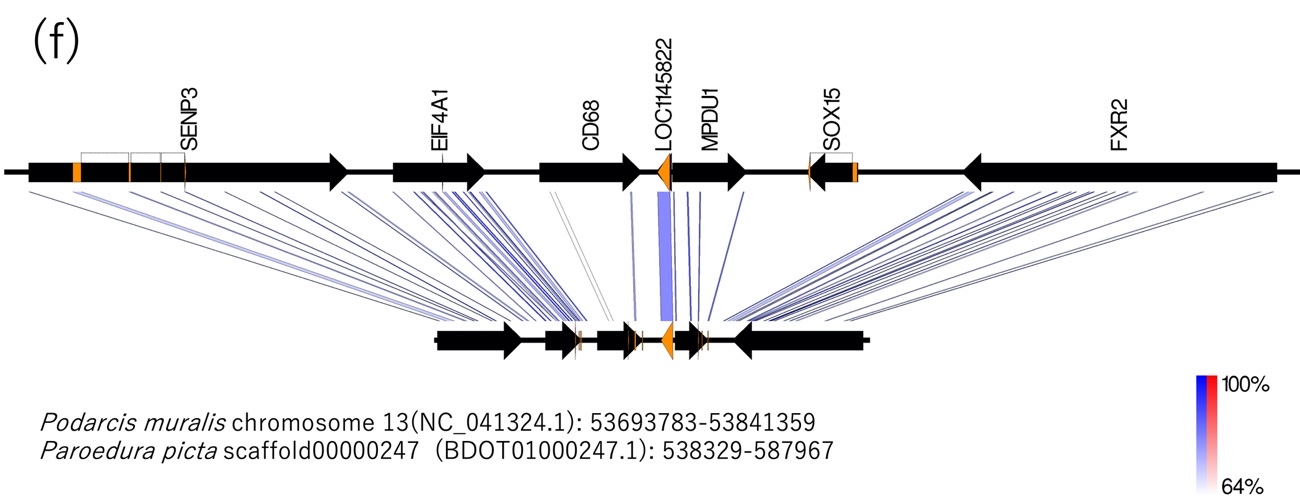

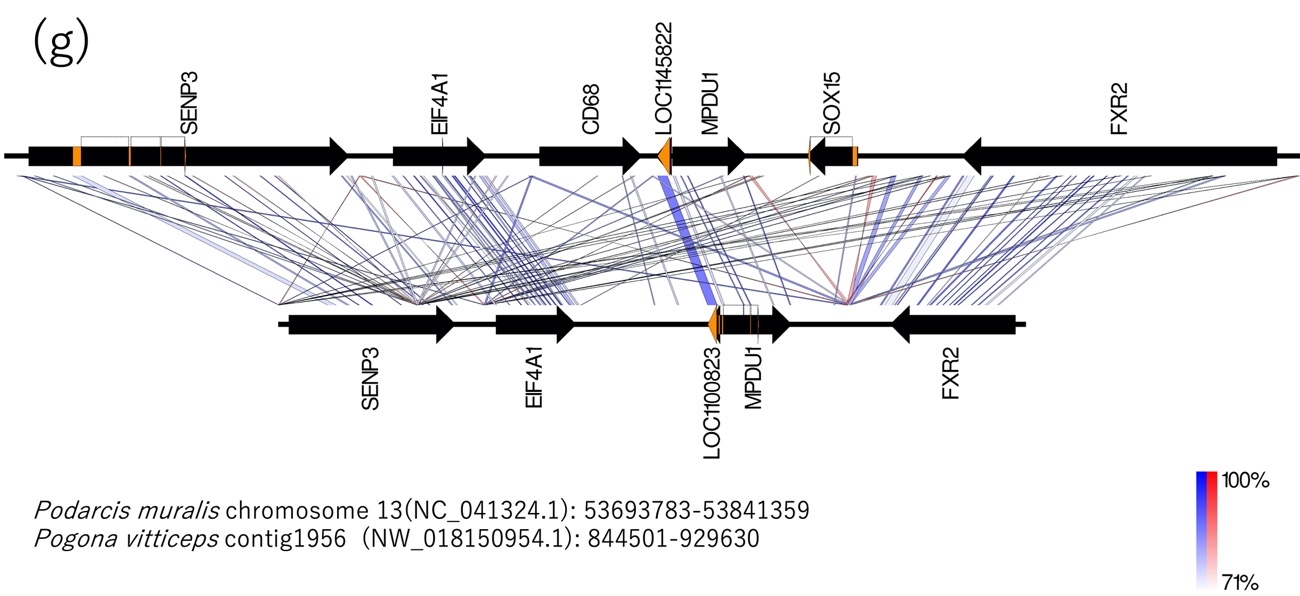

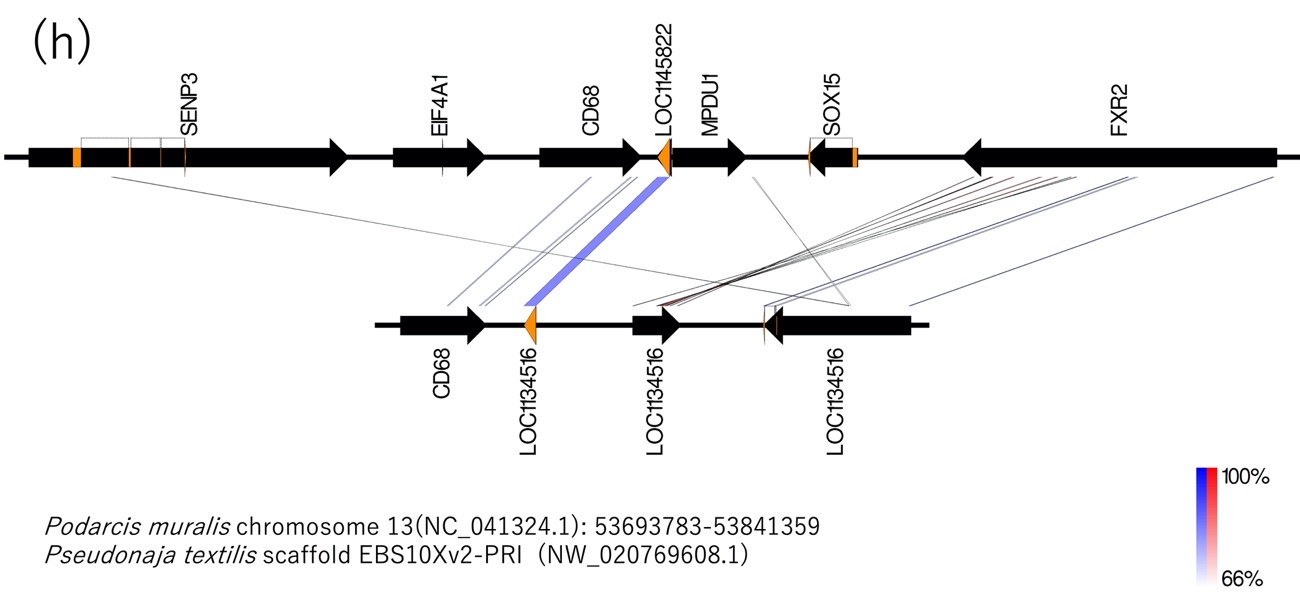

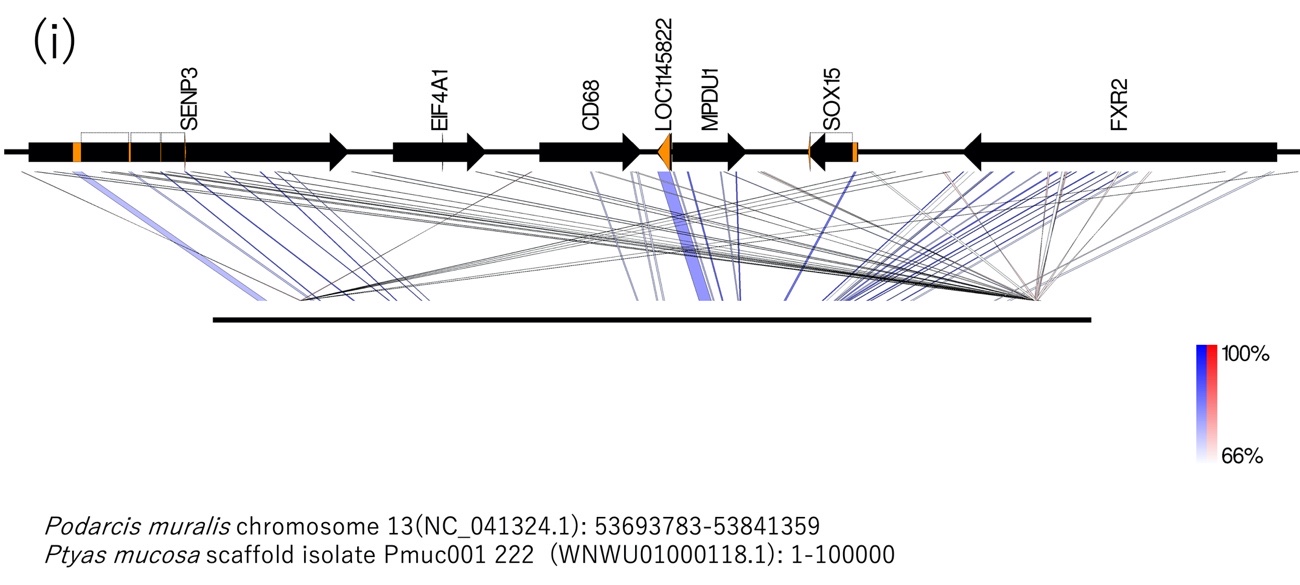

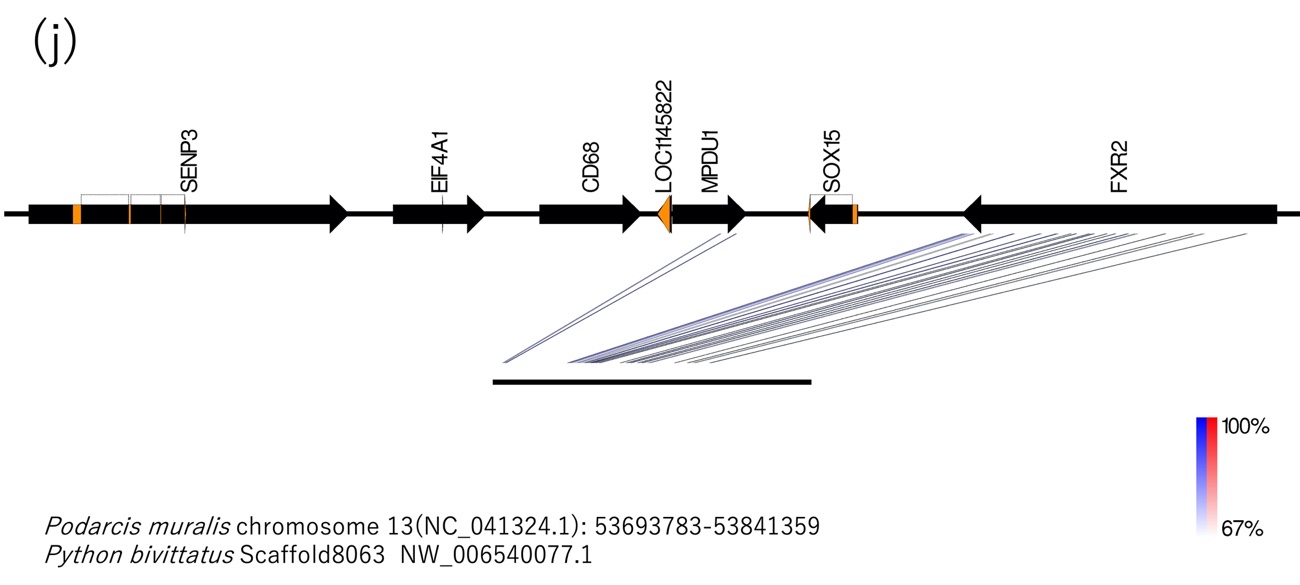

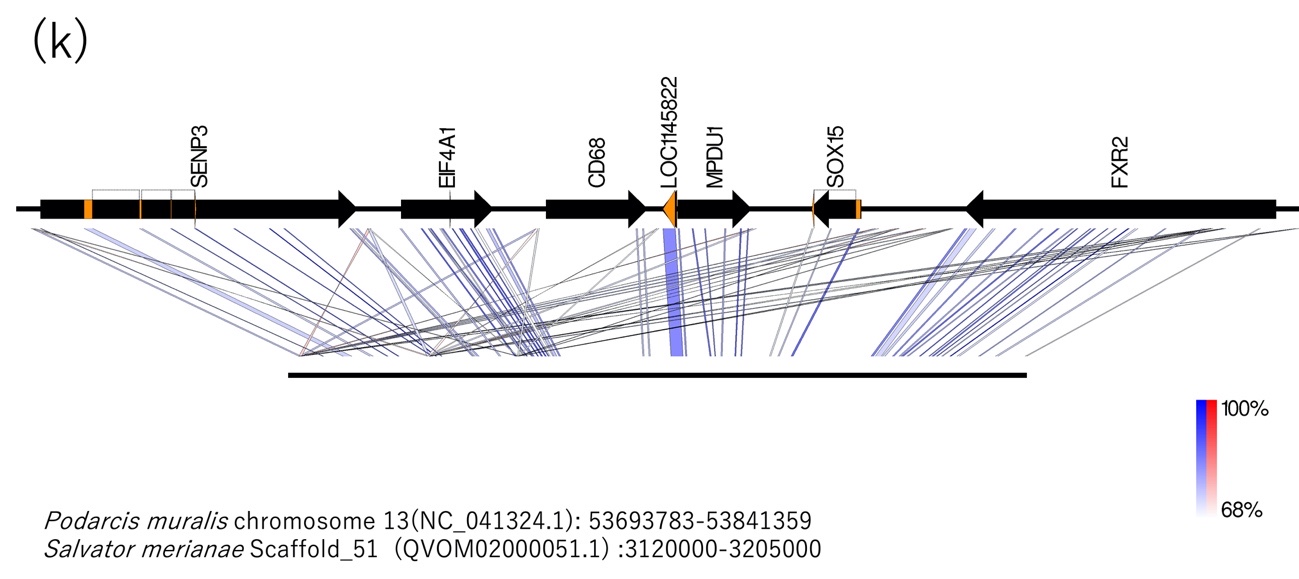

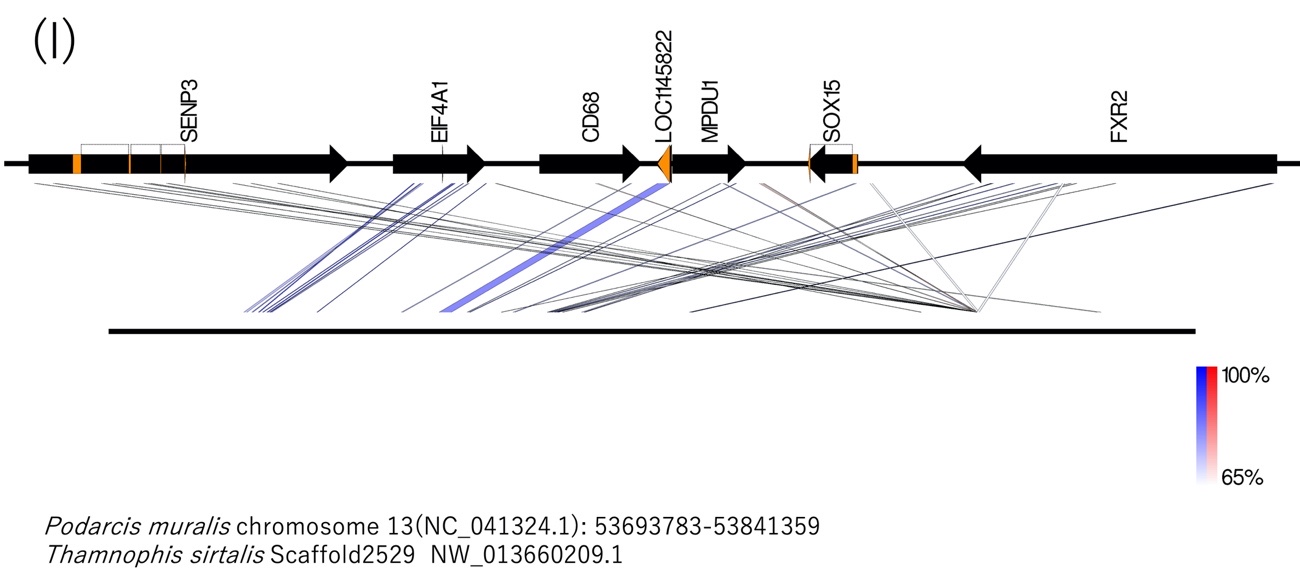

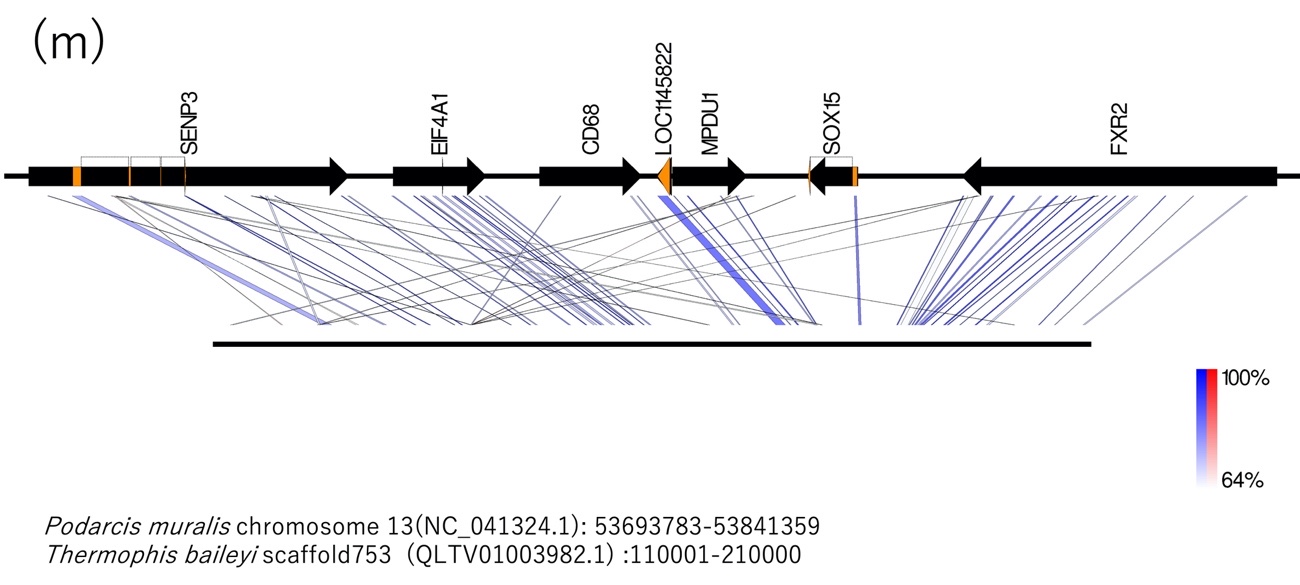

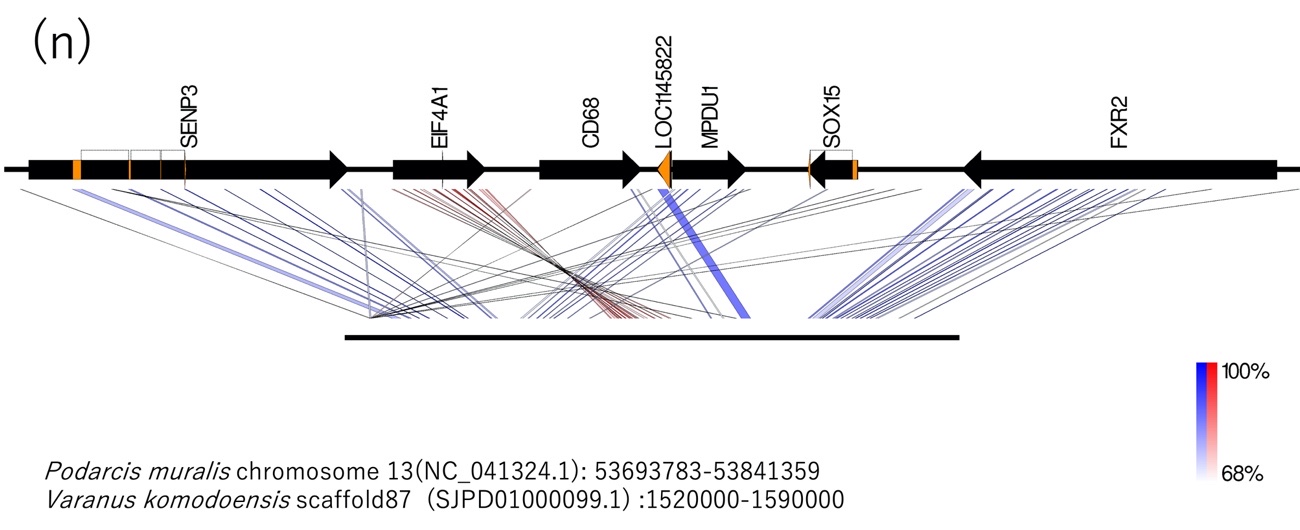

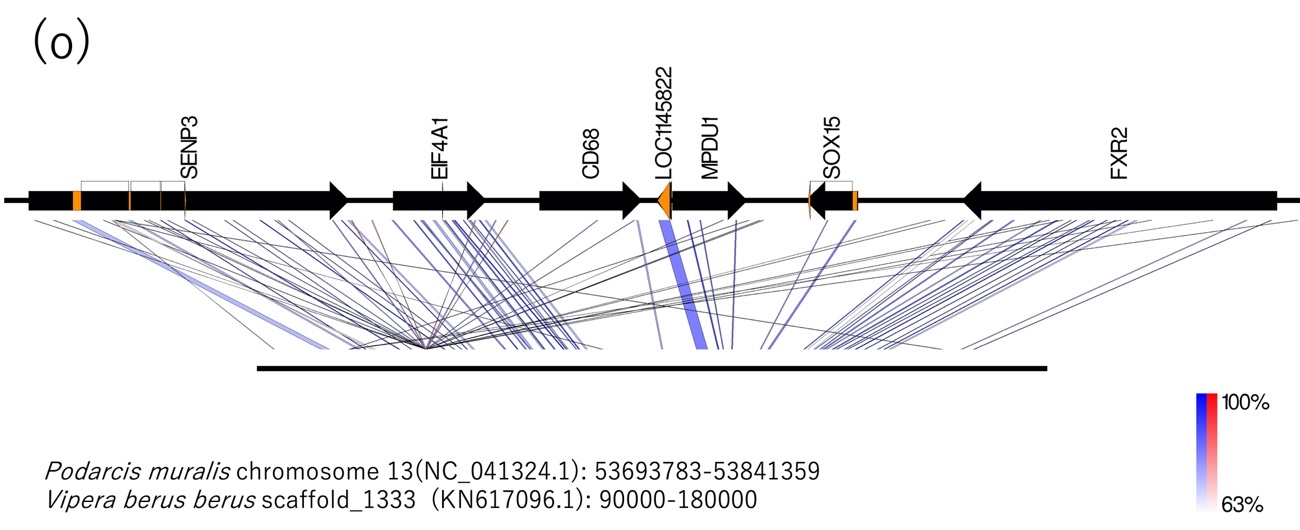

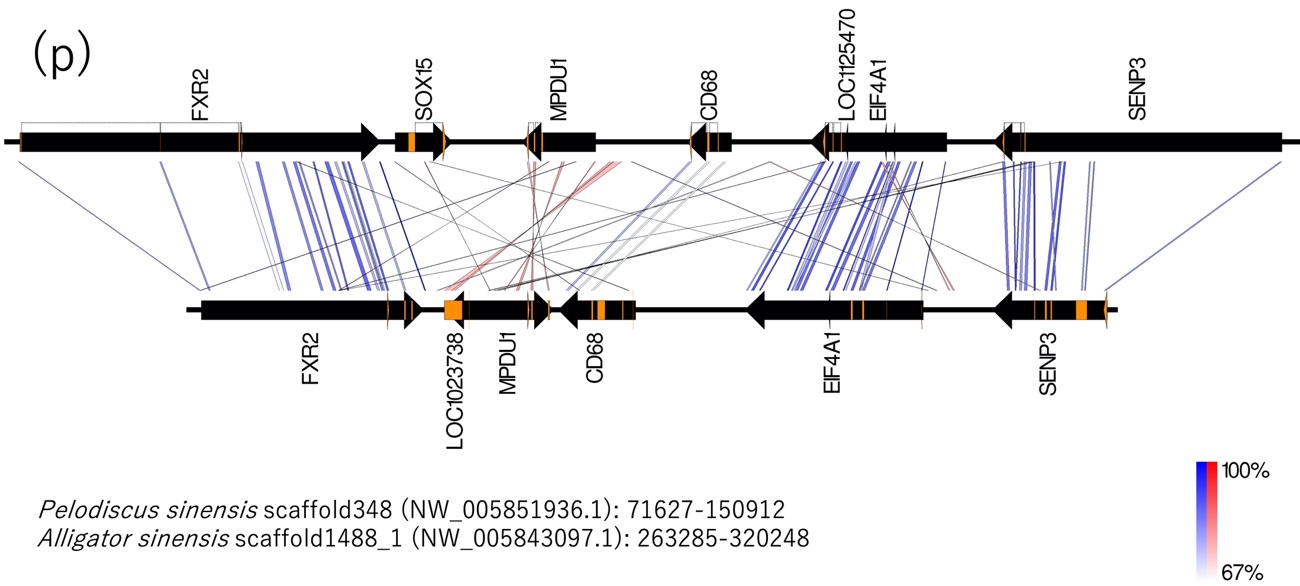

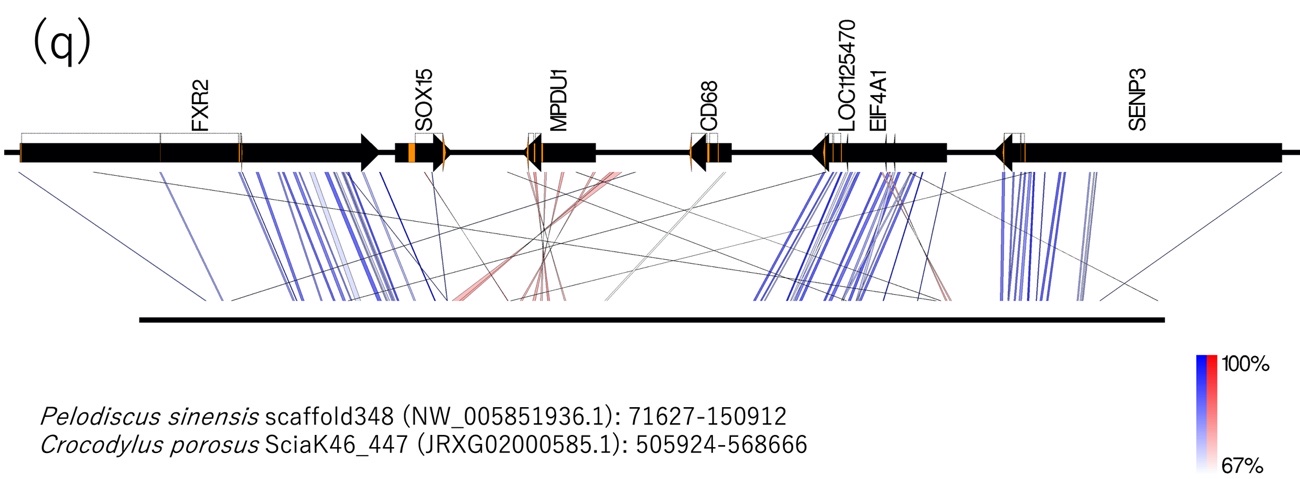

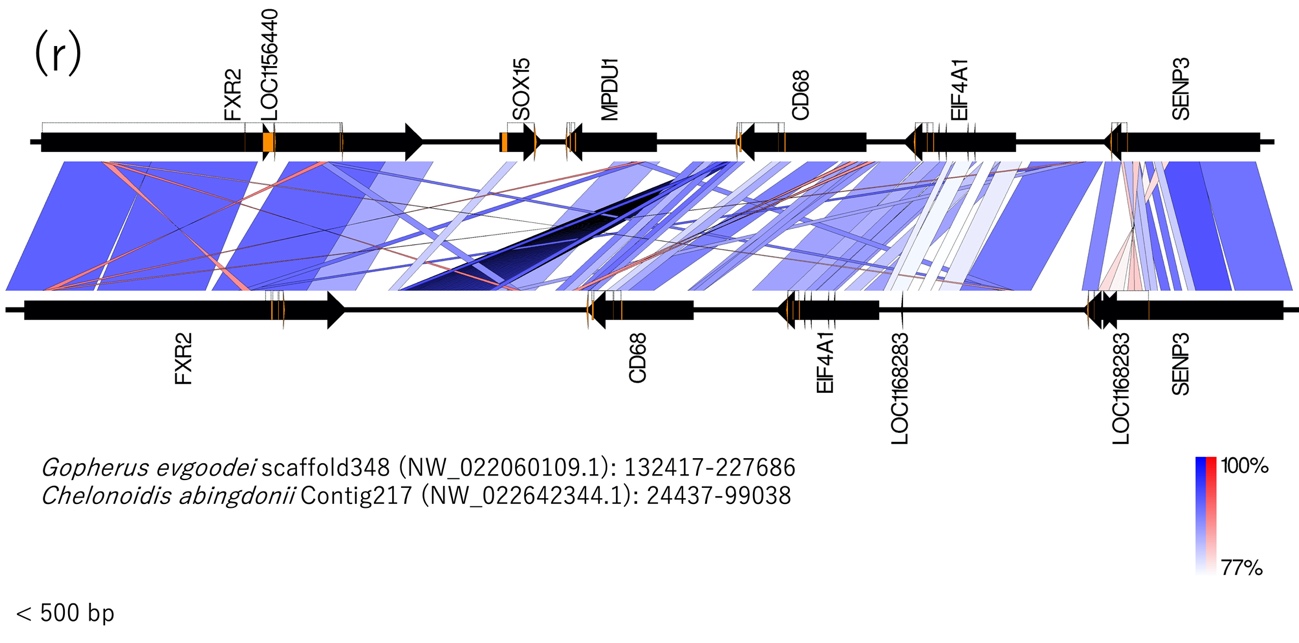

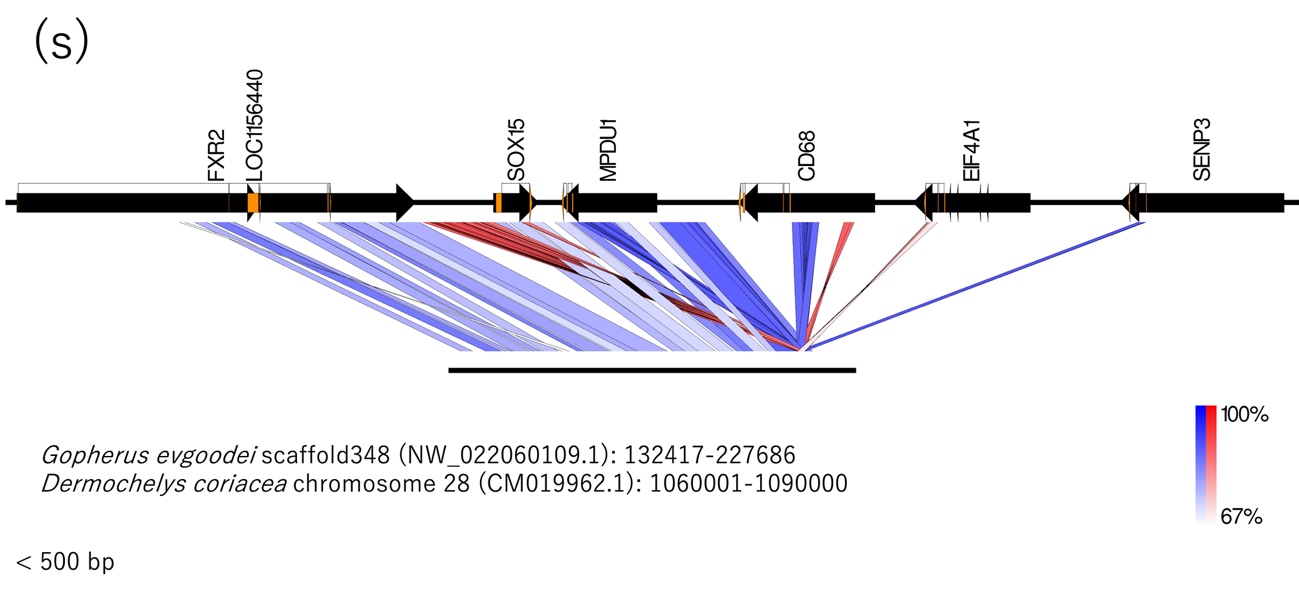

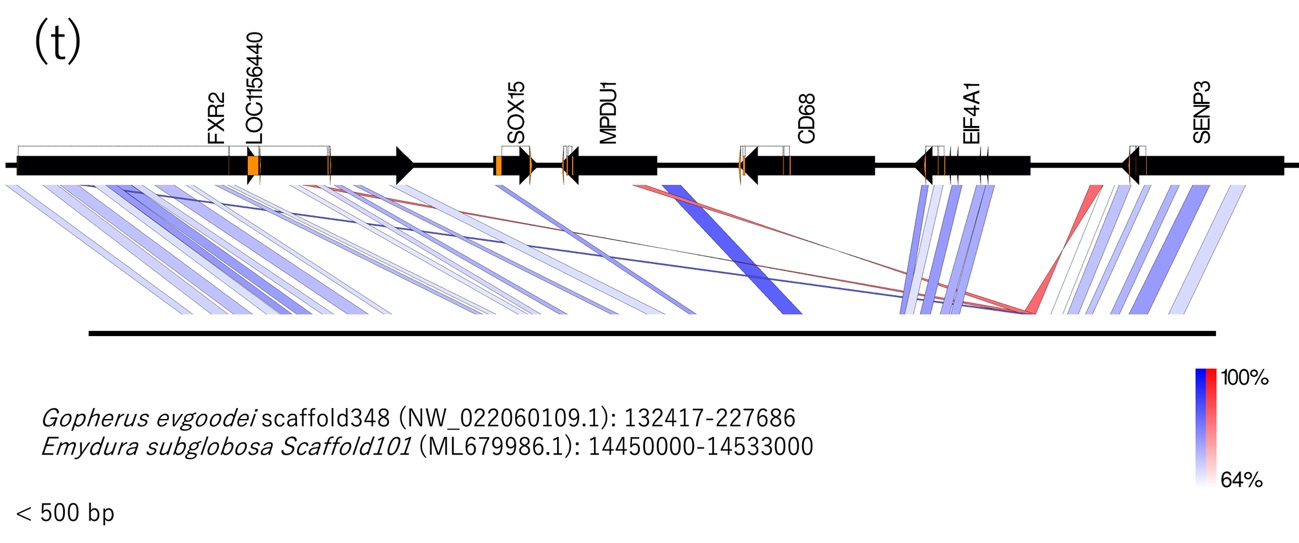

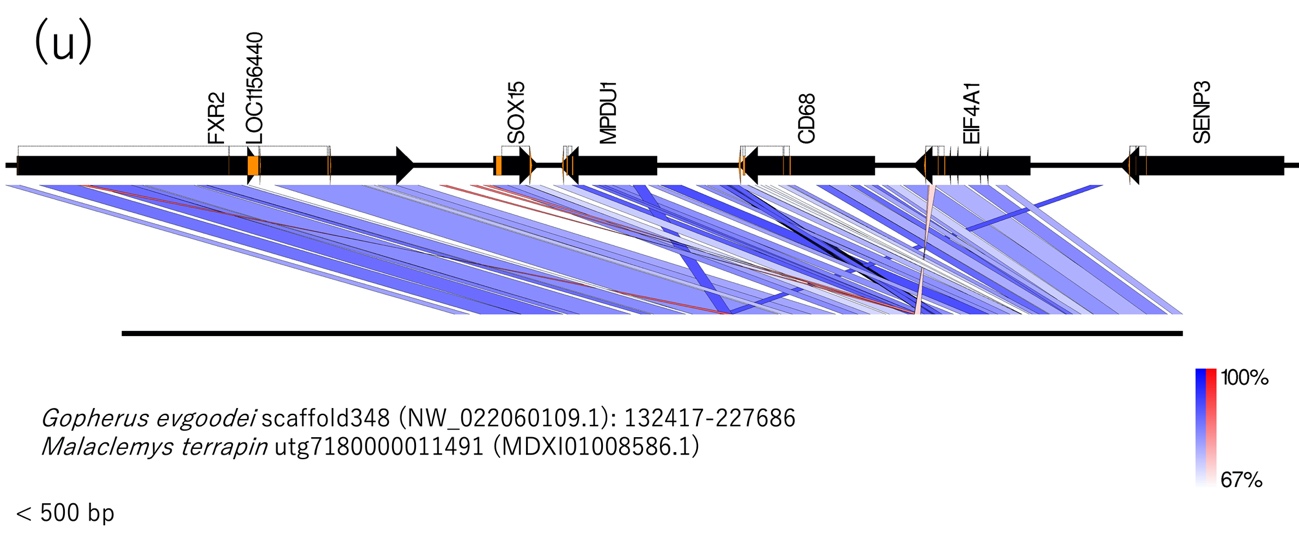

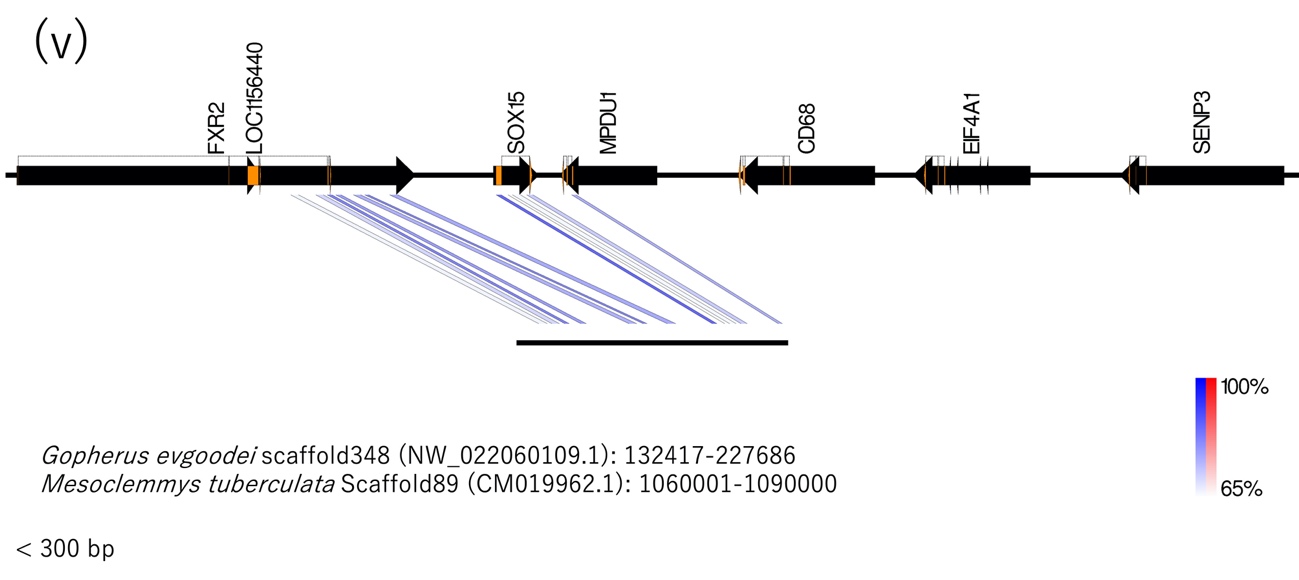

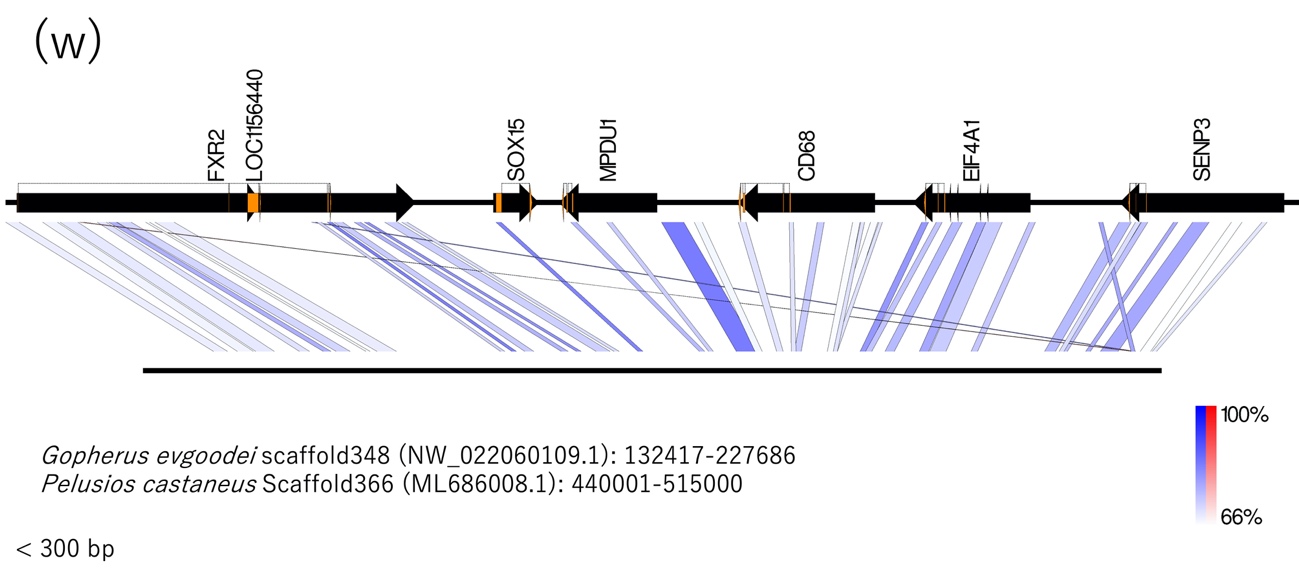

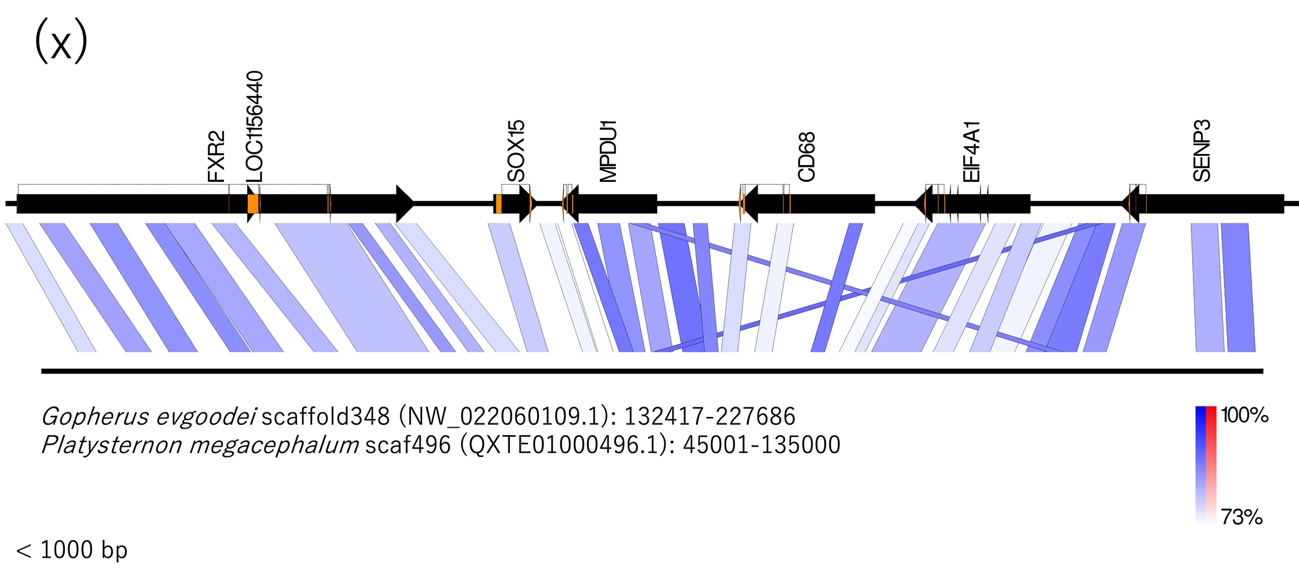

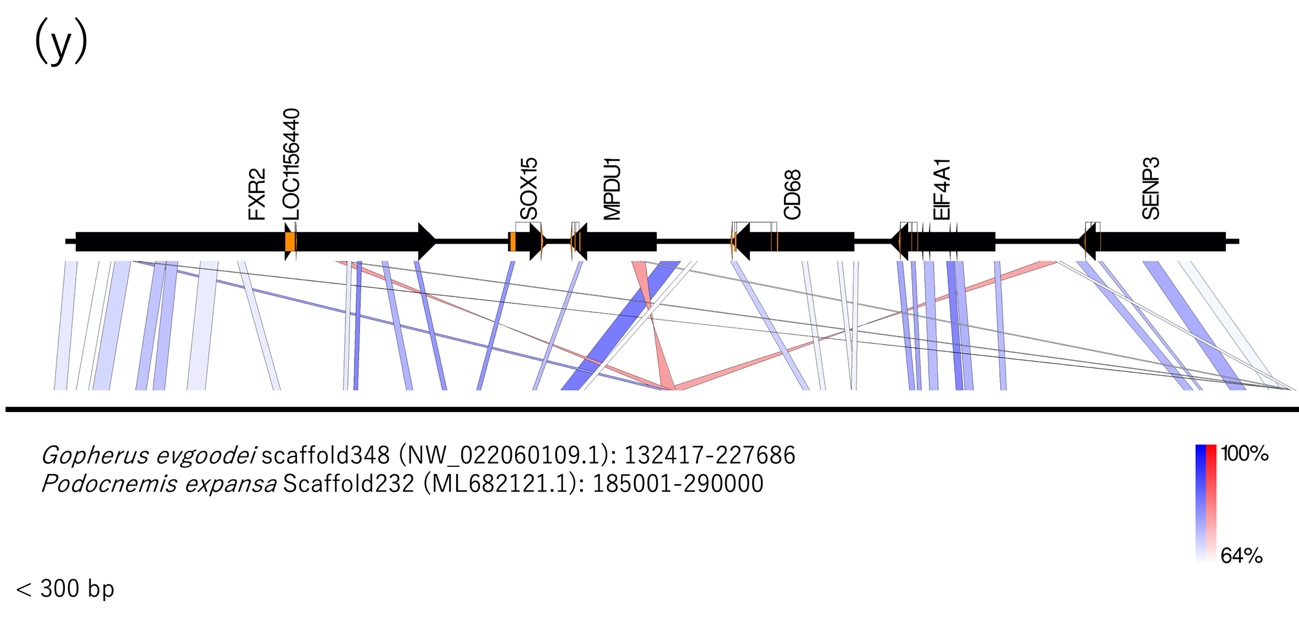


**Figure S3. Easyfig analysis for the locus encoding *fxr2* and *mpdu1* genes in reptiles.**

**(a-o)** Loci encoding *fxr2* and *mpdu1* in Squamata were compared to those of *Podarcis muralis* by blastn in default options. **(p, q)** Loci encoding *fxr2* and *mpdu1* in Crocodilia were compared to those of *Pelodiscus sinensis* by blastn in default options. **(r-y)** Loci encoding *fxr2* and *mpdu1* in Testudines were compared to that of *Pelodiscus sinensis* by blastn in default options without the hit length limited to **(v, w, y)** 300, **(r-u)** 500, and **(x)** 1000 or more. The blastn results were visualized by Eagyfig 2.2.2 with blue and red lines, which indicate the forward and reverse blastn hits. Color scale indicates identity percentage of the hits. Black arrows show regions and directions of genes. Orange boxes show exon regions of the gene.


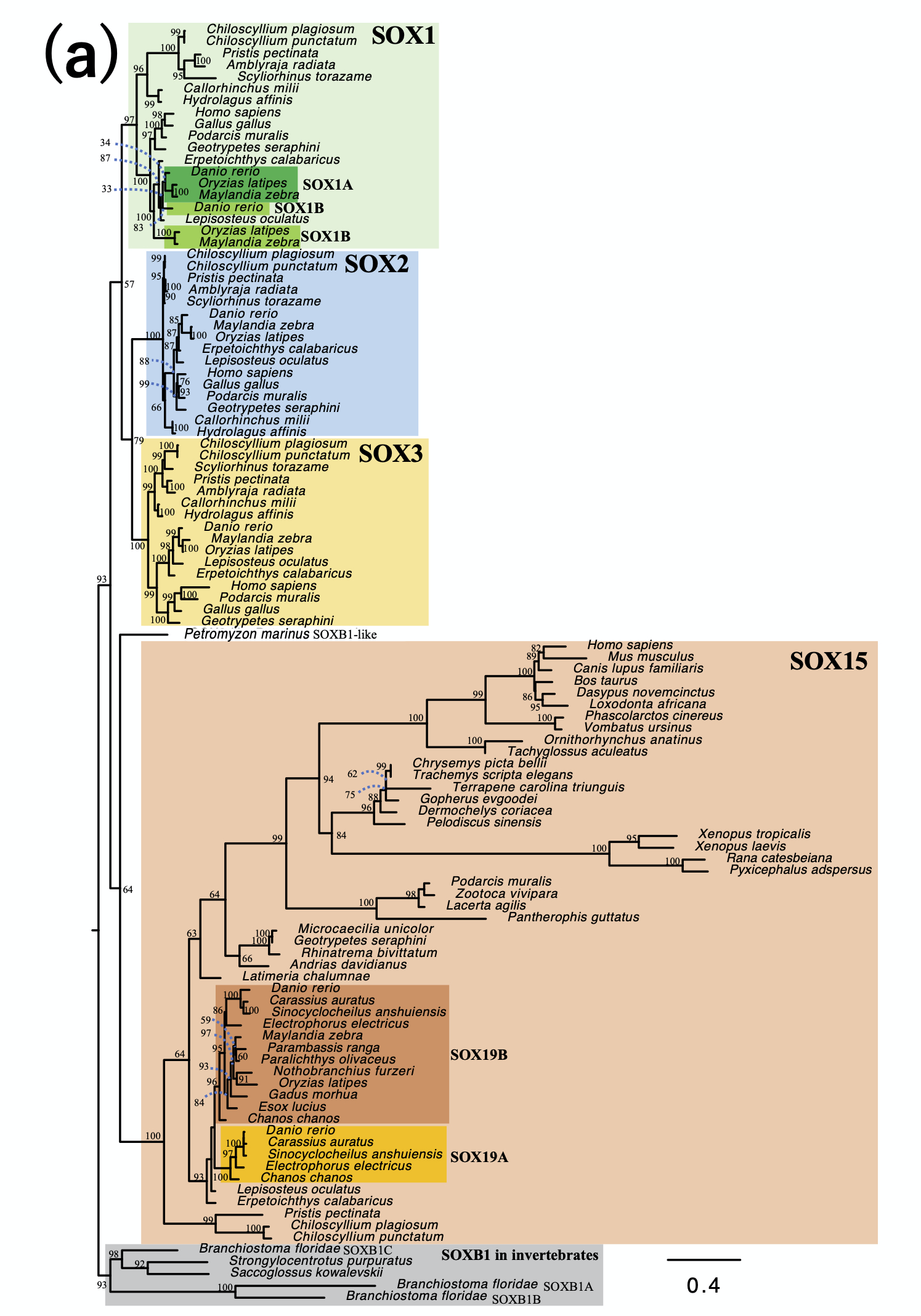


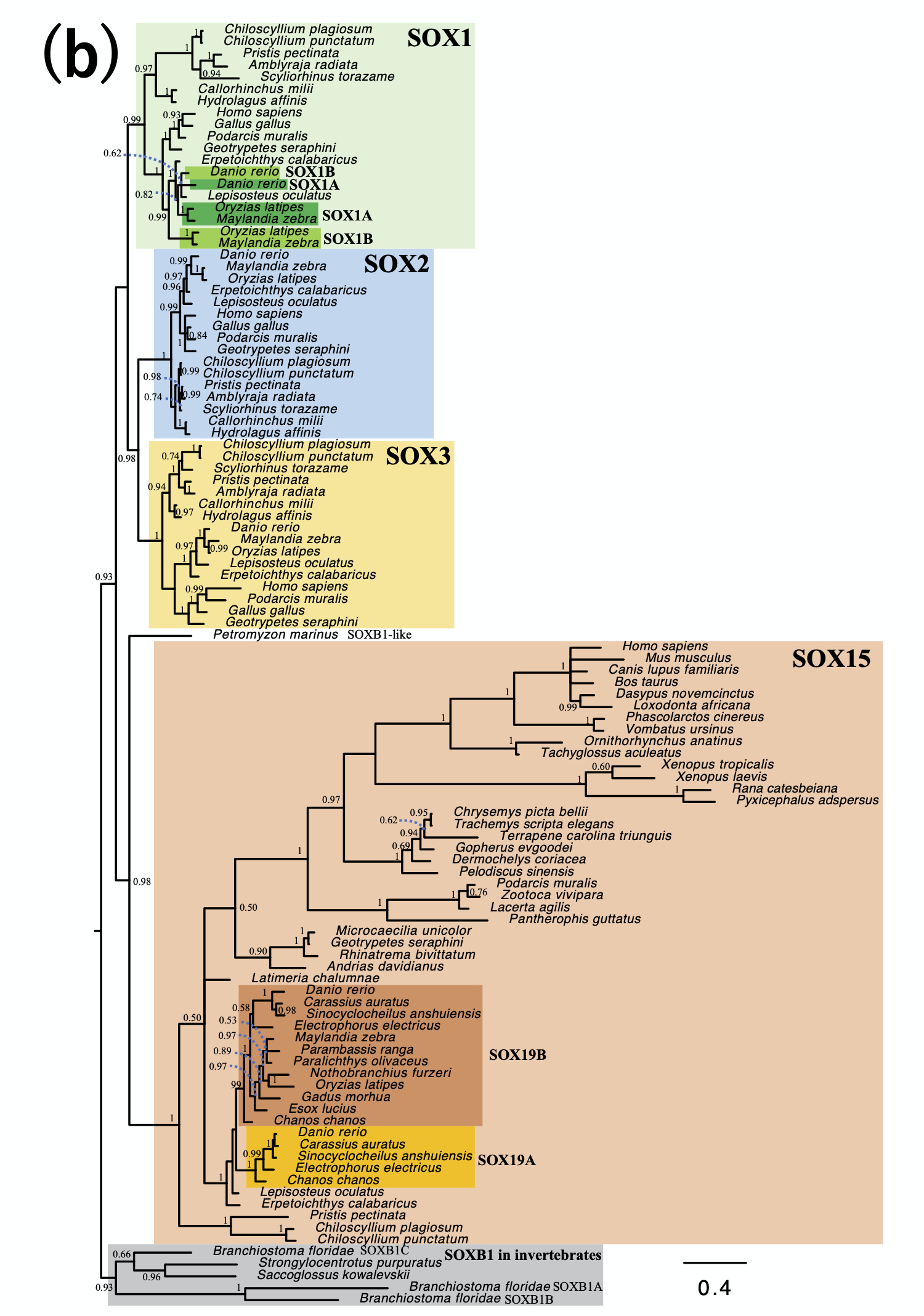


**Figure S4. Phylogenetic relationships of vertebrate *soxB1*/*G* ohnologous proteins. (a)** Maximum likelihood and **(b)** Bayesian phylogenetic trees were shown. A total of 108 aa sequences containing 273 sites were used for this tree inference. The JTT + F + I + Γ4 model was selected as the best-fit model in this dataset and used for the inference. The invertebrate SOXB1 clade was rooted. Values of **(a)** the 1000 times ultrafast bootstrap test and **(b)** the Bayesian posterior probability are shown at each node. Only the 90 ≦ bootstrap values and 0.90 ≦ posterior probabilities were shown. The scale bars indicate aa substitutions per site.

**
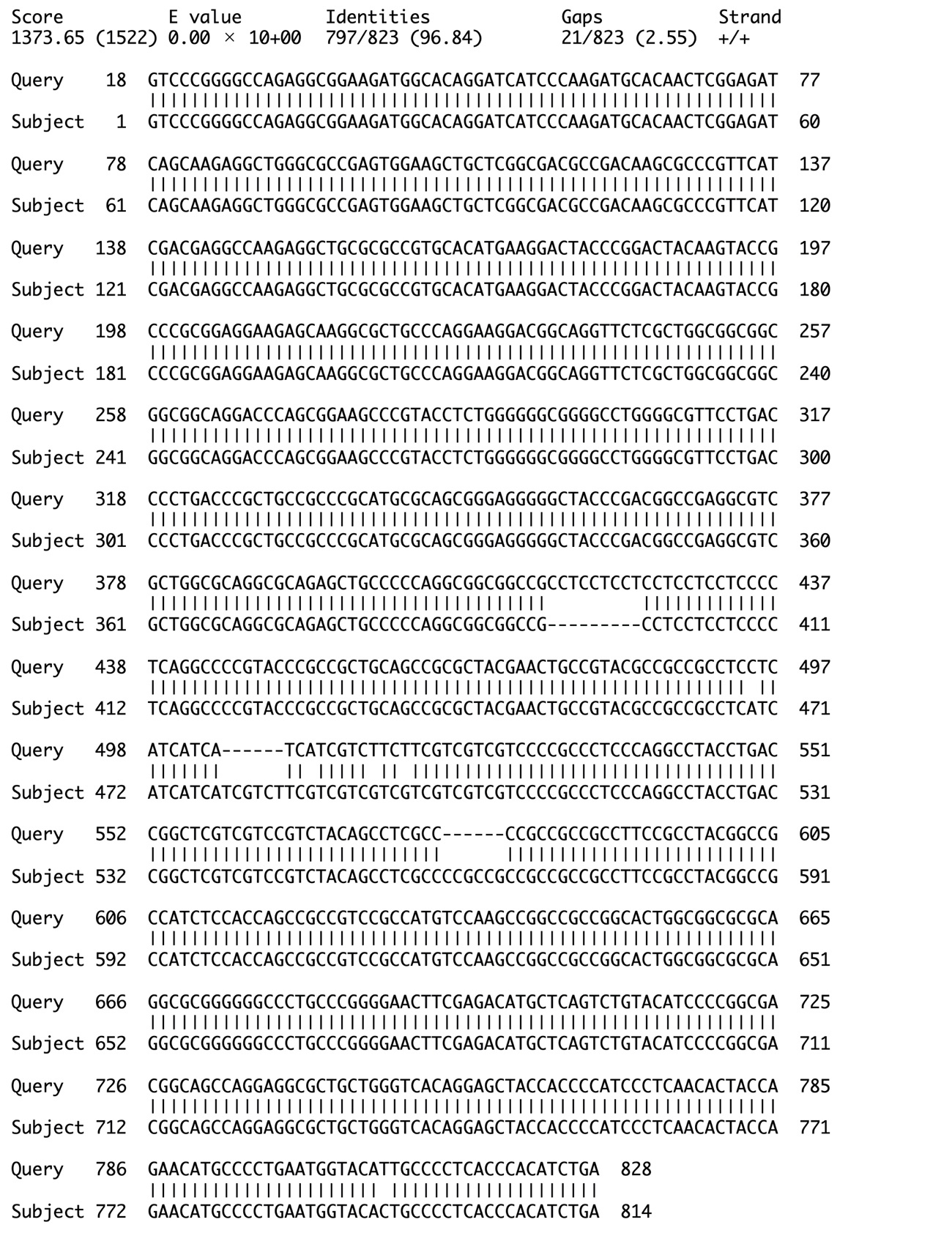
**

**Figure S5. An intron-less structure of *Chiloscyllium punctatum* ortholog of *sox15* by blastn hit.**

Annotated open reading frame of *C. punctatum* ortholog of *sox15* (BEZZ01008499.1:3-830) was used for a blastn search as a query for a database “Chiloscyllium punctatum transcript contigs” (<https://transcriptome.riken.jp/squalomix/blast/)>. “Query” was the query sequence used. “Subject” was a top hit contig “PE_TRINITY_DN183021_c7_g3_i2”.

**
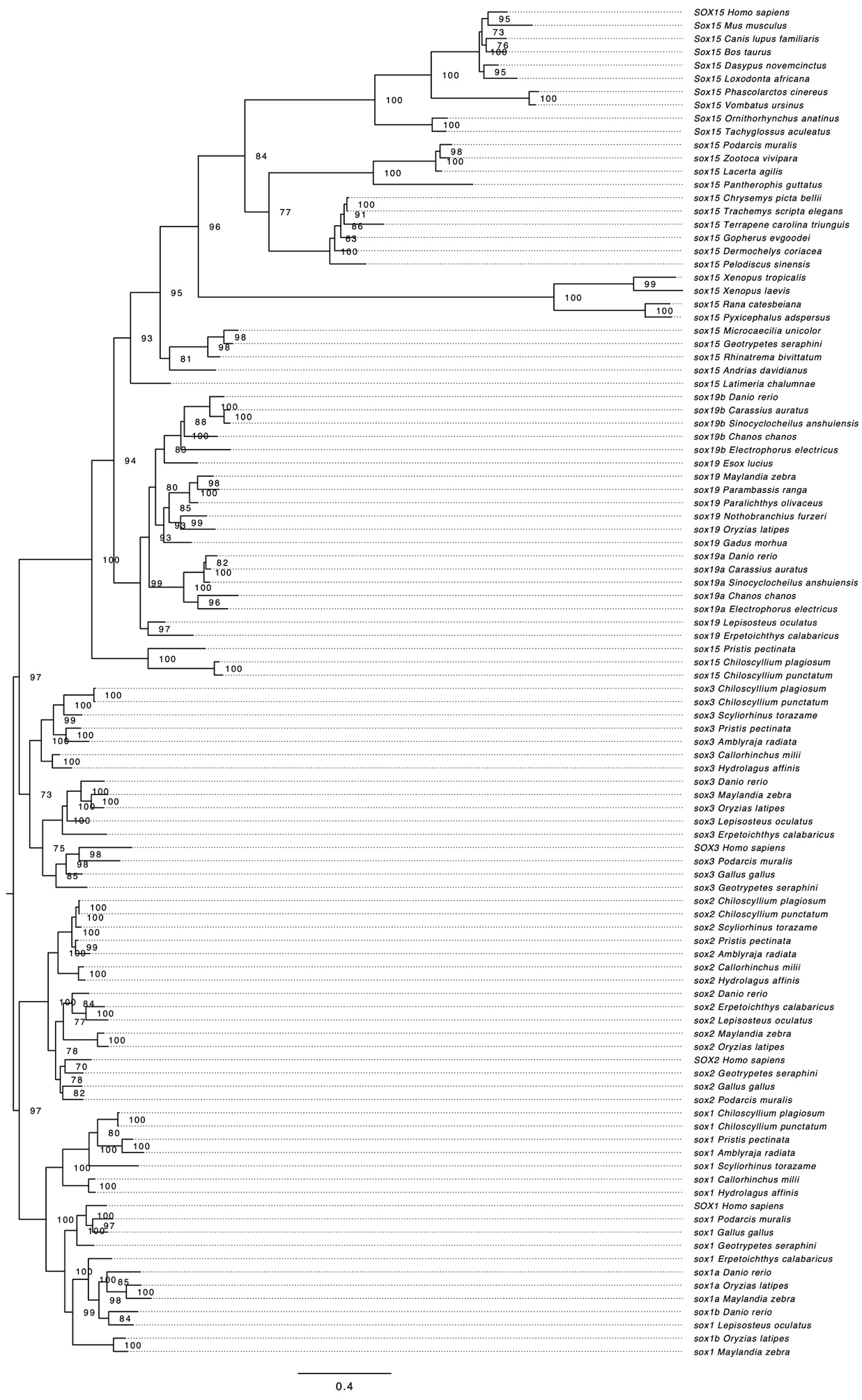
**

**Figure S6. Maximum likelihood tree of nucleotide sequences of gnathostomata *soxB1/G* ohnologs.**

A total of 102 sequences with 930 nucleotide sites were used for this tree inference. The GTR + F + R5 model was selected as the best-fit model in this dataset and used for this tree inference. Ultrafast bootstrap values 1000 times are shown for each node.
